# Repurposed Drugs That Block the Gonococcus-Complement Receptor 3 Interaction Can Prevent and Cure Gonococcal Infection of Primary Human Cervical Epithelial Cells

**DOI:** 10.1128/mBio.03046-19

**Published:** 2020-03-03

**Authors:** Jessica Poole, Christopher J. Day, Thomas Haselhorst, Freda E.-C. Jen, Victor J. Torres, Jennifer L. Edwards, Michael P. Jennings

**Affiliations:** a Institute for Glycomics, Griffith University, Southport, Queensland, Australia; b Department of Microbiology, New York University School of Medicine, New York, New York, USA; c The Center for Microbial Pathogenesis, The Abigail Wexner Research Institute at Nationwide Children’s Hospital and The Department of Pediatrics, The Ohio State University, Columbus, Ohio, USA; University of Mississippi Medical Center

**Keywords:** CD11b I-domain, CR3, complement receptor 3, Mac-1, *Neisseria gonorrhoeae*, adherence, glycosylation, gonococcal cervicitis, gonococci, multidrug resistance, pilin, repurposed drug

## Abstract

In the absence of a vaccine, multidrug-resistant Neisseria gonorrhoeae has emerged as a major human health threat, and new approaches to treat gonorrhea are urgently needed. N. gonorrhoeae pili are posttranslationally modified by a glycan that terminates in a galactose. The terminal galactose is critical for initial contact with the human cervical mucosa via an interaction with the I-domain of complement receptor 3 (CR3). We have now identified the I-domain galactose-binding epitope and characterized its galactose-specific lectin activity. Using surface plasmon resonance and cellular infection assays, we found that a peptide mimic of this galactose-binding region competitively inhibited the N. gonorrhoeae-CR3 interaction. A compound library was screened for potential drugs that could similarly prohibit the N. gonorrhoeae-CR3 interaction and be repurposed as novel host-targeted therapeutics for multidrug-resistant gonococcal infections in women. Two drugs, methyldopa and carbamazepine, prevented and cured cervical cell infection by multidrug-resistant gonococci by blocking the gonococcal-CR3 I-domain interaction.

## Abstract (precis)

Novel therapies that avert the problem of Neisseria gonorrhoeae with acquired antibiotic resistance are urgently needed. Gonococcal infection of the human cervix is initiated by an interaction between a galactose modification made to its surface appendages, pili, and the I-domain region of (host) complement receptor 3 (CR3). By targeting this crucial gonococcal–I-domain interaction, it may be possible to prevent cervical infection in females. To this end, we identified the I-domain galactose-binding epitope of CR3 and characterized its galactose lectin activity. Moreover, we identified two drugs, carbamazepine and methyldopa, as effective host-targeted therapies for gonorrhea treatment. At doses below those currently used for their respective existing indications, both carbamazepine and methyldopa were more effective than ceftriaxone in curing cervical infection *ex vivo*. This host-targeted approach would not be subject to N. gonorrhoeae drug resistance mechanisms. Thus, our data suggest a long-term solution to the growing problem of multidrug-resistant N. gonorrhoeae infections.

## INTRODUCTION

Neisseria gonorrhoeae (the gonococcus) is an exclusive human pathogen that causes the sexually transmitted infection, gonorrhea. Infections caused by N. gonorrhoeae continue to be a global intractable problem (1). Asymptomatic cervicitis generates a carrier-like state in up to 80% of N. gonorrhoeae*-*infected women (2–6) and is the primary cause for the global prevalence of N. gonorrhoeae and its disease sequelae (7). The absence of a gonococcal vaccine together with the continuing emergence of antibiotic-resistant and untreatable strains indicate that N. gonorrhoeae poses an “urgent” public health threat (8).

**FIG 1:**
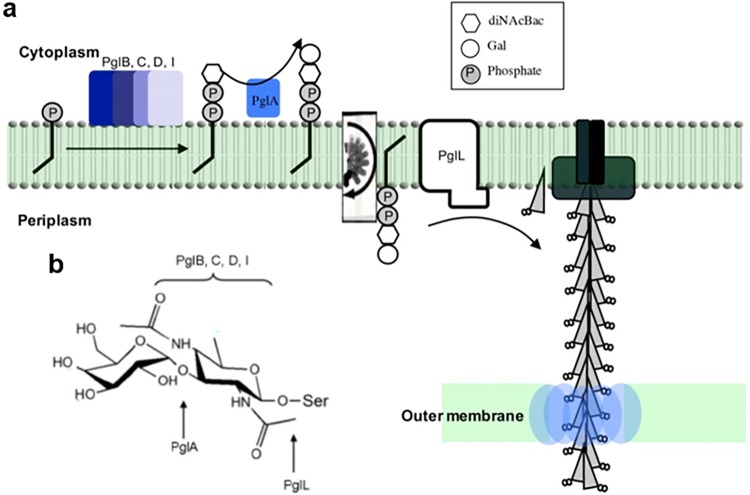
The pilin-linked disaccharide and biosynthetic pathway. Strain MS11 pilin glycosylation and pilin-linked glycan structures. (a) Pilin-linked glycan biosynthesis begins at the gonococcal cytoplasmic membrane. The pilin glycosyltransferase, PglA, transfers a galactose to a lipid-linked basal sugar, diNAcBac. The Gal(α1-3)diNAcBac lipid structure is “flipped” to the periplasm by PglF and then attached to the structural pilus subunit, PilE (at Ser63), by PglL. (b) The Gal(α1-3)diNAcBac disaccharide structure expressed on MS11 pilin when the phase-variable gene *pglA* expression is on.

N. gonorrhoeae express type IV pili (fimbriae), which are crucial to interactions with epithelial cells (9–11). These pili are polymers with variable phase and antigenic expression composed of thousands of pilin subunits encoded by the *pilE* gene (12). Pilin can be posttranslationally modified with an *O*-linked monosaccharide, *N*,*N*′-diacetylbacillosamine (diNAcBac), or a disaccharide, Gal(α1-3)diNAcBac (Fig. 1) (13–15). Pilin glycosylation results from the activity of pilin glycosyltransferase (*pgl*) gene products. For example, PglD is essential for diNAcBac biosynthesis, whereas the glycosyltransferase PglA adds the terminal galactose to Gal(α1-3)diNAcBac (16). Expression of *pglA* is phase variable (high frequency, reversible on/off switching of gene expression) (Fig. 1) (17–19). Thereby, the pilin-linked glycan can be either a disaccharide or a monosaccharide depending on *pglA* expression status (i.e., on or off).

Complement receptor 3 (CR3; also known as integrin α_M_β_2_, CD11b/CD18, and Mac-1) is an innate immune pattern recognition receptor. Expression of CR3 has historically been limited to cells of monocytic lineage; however, CR3 is also expressed on the apical surface of the human cervix (20). The alpha subunit of CR3, CD11b, contains an approximately 200-amino-acid insertion, known as the I-domain (see Fig. S1 in the supplemental material). The CR3 I-domain is the primary binding site for iC3b and many other protein ligands (21). Several important human pathogens (e.g., *Streptococcus*, *Mycobacterium*, *Toxoplasma*, and *Staphylococci*) use CR3 as a mechanism to promote disease (22–31). In this regard, CR3 is critical to N. gonorrhoeae infection of human cervical epithelial cells, both *in vivo* (20) and *ex vivo* (20, 32).

10.1128/mBio.03046-19.1 FIG S1 Complement receptor 3 I-domain sequence. Complement receptor 3 is a heterodimer composed of an alpha subunit, CD11b, and a beta subunit, CD18. CD11b contains an ∼200-amino-acid insertion domain, or I-domain, and a lectin domain. The human I-domain of CD11b (accession NP_000623.2, amino acids Gly127 to Ala325) is 77% similar to the mouse I-domain (accession NM_001082960.1, amino acids Gly127 to Ala334). *, identical amino acids; •, similar amino acids. The G2 peptide region is boxed in red. Download FIG S1, PDF file, 0.08 MB. Copyright © 2020 Poole et al. 2020 Poole et al. This content is distributed under the terms of the Creative Commons Attribution 4.0 International license.

We previously reported the fundamental discovery that the N. gonorrhoeae*-*CR3 interaction occurs solely through the I-domain (32) and is mediated by the pilin-linked glycan (33). Infection of primary human cervical epithelial (Pex) cells requires pili with Gal(α1-3)diNAcBac. Bacteria expressing a diNAcBac monosaccharide do not survive Pex cell infection (33). Given the important role of CR3 in numerous human infections, we sought to define the kinetics and specificity of this novel lectin function for the human CR3 I-domain and apply this information to the development of a novel strategy to prevent and cure gonococcal infections in women.

## RESULTS

### The gonococcal pilin glycan interacts with the human I-domain.

Jennings et al. showed that a direct interaction occurs between the N. gonorrhoeae pilin-linked glycan and the CR3 I-domain (33). This was a major finding, as previous literature ascribed CR3 carbohydrate binding to a separate lectin domain (see Fig. S1 in the supplemental material). To further define the pilin-linked glycan-CR3 I-domain interaction, we performed surface plasmon resonance (SPR). Gonococcal pilin with diNAcBac or Gal(α1-3)diNAcBac (Fig. 1) were flowed over immobilized recombinant (r)I-domain or rCR3 protein (Table 1). A high-affinity interaction was observed between N. gonorrhoeae MS11 wild-type pili (disaccharide glycan) and both human rI-domain (dissociation constant [K_D_], 349 nM) and rCR3 protein (K_D_, 907 nM). This high-affinity interaction was dependent upon the terminal galactose of Gal(α1-3)diNAcBac, as no interaction was detected for MS11 *pglA* pili, which have diNAcBac, in the concentration range tested (Table 1; Fig. S2). MS11 wild-type- and *pglA* mutant-derived pili did not bind to mouse rI-domain in a glycan-dependent manner (Table 1; Fig. S2).

**TABLE 1:** Surface plasmon resonance analysis of the gonococcal pilin-CR3 interaction^*a*^

Strain	Glycan	K_D_ (nM)		
		rI-domain		Human CR3
		Mouse	Human	
MS11 wild type	Gal(α1-3) diNAcBac	NCDI	349 ± 45	907 ± 43.8
MS11 *pgl*A	diNAcBac	NCDI	NCDI	NCDI

a The interaction between N. gonorrhoeae MS11 pilin modified with either a disaccharide or a monosaccharide and mouse rI-domain, human rI-domain, and human rCR3 was characterized by SPR analysis. Results listed are the means ± standard errors from three replicate experiments. No concentration-dependent interaction (NCDI; no binding of ≤2 μM glycan to immobilized protein) was observed with either of the gonococcal pilins and the mouse rI-domain. The human rI-domain and rCR3 showed NCDI with MS11 *pglA* pilin. A high-affinity interaction (nanomolar range) was only observed between the disaccharide modified pilin and both the human rI-domain and rCR3.

10.1128/mBio.03046-19.2 FIG S2 Blank- and reference-subtracted SPR sensorgrams for results in Tables 1 and 2. These sensorgrams are single replicates of triplicate data. (a) Five dilutions (0.125 to 2 μM) of *pglA* (blue) or wild-type (WT; red) pilin were flowed over immobilized human rI-domain. The sensorgrams show a negative response with every pglA pilin injection, whereas positive response units (RU) and an RU build are seen with WT pilin injections. (b) Five dilutions (0.125 to 2 μM) of *pglA* or WT pilin were flowed over a flow cell to which rCR3 was immobilized. (c) Five dilutions (0.125 to 2 μM) of *pglA* or WT pilin were flowed over mouse rI-domain immobilized to a flow cell. Note that although an RU build (shown by the black arrows) was detected for pili in the upper concentration ranges shown in panel C, recombinant pili could not be maintained in solution at these higher concentrations; therefore, it was not possible to approach saturation. As a result of this issue and with the negative RU response observed during pilin injections (shown by green arrows), we could not determine a K_D_ for recombinant pilin with the mouse rI-domain. (d) Nine dilutions (0.0039 to 1 μM) of α1-3,β1-4,α1-3 galactotetraose over immobilized I-domain. (e) Nine dilutions (0.0039 to 1 μM) of linear β-2 trisaccharide over immobilized I-domain. (f) Nine dilutions (0.0039 to 1 μM) of P1 antigen over immobilized I-domain. (g) Nine dilutions (0.0039 to 1 μM) of α1-3 galactobiose over immobilized I-domain. (h) Nine dilutions (0.0039 to 1 μM) of lactose over immobilized I-domain. (i) Nine dilutions (0.0039 to 1 μM) of α-lactose over immobilized I-domain. (j) Nine dilutions (0.0039 to 1 μM) of methyl-α-d-galactopyranoside over immobilized I-domain. (k) Eight dilutions (0.0078 to 1 μM) of sucrose (negative control) over immobilized I-domain. The RUs are due to bulk shift. There is no build in RU after injections as with the other carbohydrates. (l) Nine dilutions (0.0039 to 1 μM) of α1-3,β1-4,α1-3 galactotetraose over immobilized rCR3. (m) Nine dilutions (0.0039 to 1 μM) of linear β-2 trisaccharide over immobilized rCR3. (n) Nine dilutions (0.0039 to 1 μM) of P1 antigen over immobilized rCR3. (o) Nine dilutions (0.0039 to 1 μM) of α1-3 galactobiose over immobilized rCR3. (p) Nine dilutions (0.0039 to 1 μM) of lactose over immobilized rCR3. (q) Nine dilutions (0.0039 to 1 μM) of α-lactose over immobilized rCR3. (r) Nine dilutions (0.0039 to 1 μM) of methyl-α-d-galactopyranoside over immobilized rCR3. (s) Eight dilutions (0.0078 to 1 μM) of sucrose (negative control) over immobilized rCR3. The RUs are due to bulk shift. There is no build in RU after injections as with the other carbohydrates. (t) Five dilutions (0.0625 to 1 μM) of α1-3 galactobiose over immobilized biotin-AhxG2-streptavidin complex. (u) Five dilutions (0.0625 to 1 μM) of methyl-α-d-galactopyranoside over immobilized biotin-AhxG2-streptavidin complex. (v) Five dilutions (0.0625 to 1 μM) of sucrose (negative control) over immobilized biotin-AhxG2-streptavidin complex. The RUs are due to bulk shift. Download FIG S2, PDF file, 0.7 MB. Copyright © 2020 Poole et al. 2020 Poole et al. This content is distributed under the terms of the Creative Commons Attribution 4.0 International license.

### The human I-domain has high-affinity galactose-specific lectin activity.

SPR analyses showed an interaction between the pilin-linked glycan and the I-domain and suggested this interaction required the terminal α-galactose. To confirm the galactose–I-domain interaction in the absence of pilin protein components, we again performed SPR. Synthetic glycans with a terminal α1-3 galactose, similar to the pilin disaccharide, were flowed across immobilized human rI-domain and rCR3 (Table 2; Fig. S2). These structures bound with high affinity to the rI-domain (dissociation constant [K_D_], 229 to 320 nM) and rCR3 (K_D_, 26.2 to 68.7 nM). We then investigated the effects of linkage and anomeric configuration on galactose recognition. Synthetic glycans with a terminal α1-4 galactose bound with similar affinity to α1-3 galactose: rI-domain (K_D_, ∼202 nM) and rCR3 (K_D_, 48.3 to 79.8 nM). Synthetic glycans linked with a terminal β1-3 or β1-4 galactose also bound with high affinity (Table 2). Methyl-α-d-galactose monosaccharide was used to confirm that the observed glycan–I-domain interaction occurred through the terminal galactose versus an underlying alternative sugar. Sucrose, which has a terminal glucose, did not bind to rI-domain or rCR3. Taken together, these data indicated that the I-domain has high-affinity galactose lectin activity that is independent of anomeric configuration and linkage.

**TABLE 2:**
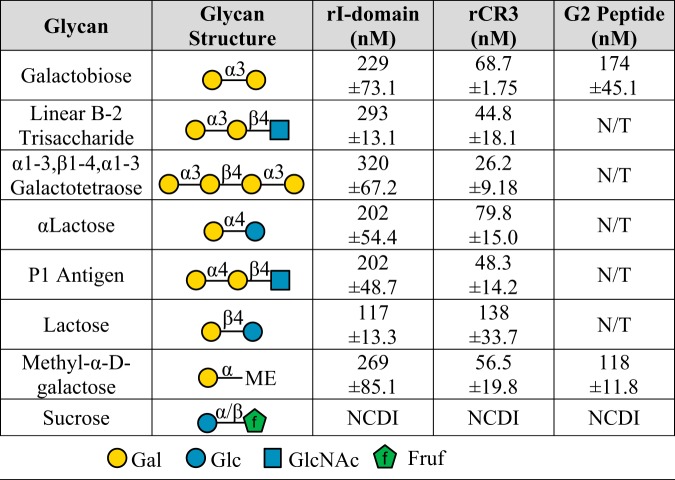
Surface plasmon resonance analysis of carbohydrates similar to the pilin disaccharide^*a*^

a To determine the galactose specificity of the interaction between the I-domain and the gonococcal pilin-linked disaccharide, SPR was performed using carbohydrates with terminal α- or β-galactose in various linkages. Results shown are the means ± standard errors from three replicate experiments (N/T; not tested). These data showed that the human I-domain can form a high-affinity interaction with various terminal galactose structures. The G2 peptide is a biotin-Ahx-labeled peptide (Ahx is a six-carbon spacer). Sucrose was used as a negative control and showed no concentration-dependent interaction (NCDI; no interaction at any concentration between 0.0078 μM and 2 μM).

### Mapping the I-domain galactose-specific lectin activity.

Our data suggested that the I-domain has high-affinity lectin activity. Herein, we took the strategy of mapping the galactose-binding epitope using a tiled array of peptides. Forty overlapping 15-amino-acid peptides were synthesized that covered the entire 208-amino-acid sequence of the human I-domain (amino acids Gly127 to Ala325 of CD11b, accession number NP_000623.2) (Fig. S1 and Table S1). These peptides were used in SPR assays to assess their ability to block galactose from binding to rI-domain and/or rCR3. Galactobiose (Galα1-3Gal) was chosen as the ligand because of its high affinity for human rI-domain and rCR3 (Table 2) as well as its similarity to the pilin disaccharide. Peptide-galactobiose mixtures (molar ratio of 25:1) or peptide only (control) were flowed over immobilized human rI-domain and rCR3. This screen revealed one peptide, named G2, that was able to block galactobiose binding to rI-domain and rCR3 (see Fig. S3). The G2 peptide region has the amino acid sequence RIHFTFKEFQNNPNP, which corresponds to amino acids Arg197 to Pro211 of human CD11b (Fig. S1).

10.1128/mBio.03046-19.3 FIG S3 I-domain peptide library competition SPR screen sensorgrams with the carbohydrate galactobiose (1-3Gal) and I-domain flow cell. (a) I-domain library peptide A1 only. (b) I-domain library peptide A1+1-3Gal. (c) I-domain library peptide A2 only. (d) I-domain library peptide A2+1-3Gal. (e) I-domain library peptide A3 only. (f) I-domain library peptide A3+1-3Gal. (g) I-domain library peptide A4 only. (h) I-domain library peptide A4+1-3Gal. (i) I-domain library peptide A5 only. (j) I-domain library peptide A5+1-3Gal. (k) I-domain library peptide B1 only. (l) I-domain library peptide B1+1-3Gal. (m) I-domain library peptide B2 only. (n) I-domain library peptide B2+1-3Gal. (o) I-domain library peptide B3 only. (p) I-domain library peptide B3+1-3Gal. (q) I-domain library peptide B4 only. (r) I-domain library peptide B4+1-3Gal. (s) I-domain library peptide B5 only. (t) I-domain library peptide B5+1-3Gal. (u) I-domain library peptide C1 only. (v) I-domain library peptide C1+1-3Gal. (w) I-domain library peptide C2 only. (x) I-domain library peptide C2+1-3Gal. (y) I-domain library peptide C3 only. (z) I-domain library peptide C3+1-3Gal. (aa) I-domain library peptide C4 only. (ab) I-domain library peptide C4+1-3Gal. (ac) I-domain library peptide C5 only. (ad) I-domain library peptide C5+1-3Gal. (ae) I-domain library peptide D1 only. (af) I-domain library peptide D1+1-3Gal. (ag) I-domain library peptide D2 only. (ah) I-domain library peptide D2+1-3Gal. (ai) I-domain library peptide D3 only. (aj) I-domain library peptide D3+1-3Gal. (ak) I-domain library peptide D4 only. (al) I-domain library peptide D4+1-3Gal. (am) I-domain library peptide D5 only. (an) I-domain library peptide D5+1-3Gal. (ao) I-domain library peptide E1 only. (ap) I-domain library peptide E1+1-3Gal. (aq) I-domain library peptide E2 only. (ar) I-domain library peptide E2+1-3Gal. (as) I-domain library peptide E3 only. (at) I-domain library peptide E3+1-3Gal. (au) I-domain library peptide E4 only. (av) I-domain library peptide E4+1-3Gal. (aw) I-domain library peptide E5 only. (ax) I-domain library peptide E5+1-3Gal. (ay) I-domain library peptide F1 only. (az) I-domain library peptide F1+1-3Gal. (ba) I-domain library peptide F2 only. (bb) I-domain library peptide F2+1-3Gal. (bc) I-domain library peptide F3 only. (bd) I-domain library peptide F3+1-3Gal. (be) I-domain library peptide F4 only. (bf) I-domain library peptide F4+1-3Gal. (bg) I-domain library peptide F5 only. (bh) I-domain library peptide F5+1-3Gal. (bi) I-domain library peptide G1 only. (bj) I-domain library peptide G1+1-3Gal. (bk) I-domain library peptide G2 only. (bl) I-domain library peptide G2+1-3Gal. (bm) I-domain library peptide G3 only. (bn) I-domain library peptide G3+1-3Gal. (bo) I-domain library peptide G4 only. (bp) I-domain library peptide G4+1-3Gal. (bq) I-domain library peptide G5 only. (br) I-domain library peptide G5+1-3Gal. (bs) I-domain library peptide H1 only. (bt) I-domain library peptide H1+1-3Gal. (bu) I-domain library peptide H2 only. (bv) I-domain library peptide H2+1-3Gal. (bw) I-domain library peptide H3 only. (bx) I-domain library peptide H3+1-3Gal. (by) I-domain library peptide H4 only. (bz) I-domain library peptide H4+1-3Gal. (ca) I-domain library peptide H5 only. (cb) I-domain library peptide H5+1-3Gal. Download FIG S3, PDF file, 0.6 MB. Copyright © 2020 Poole et al. 2020 Poole et al. This content is distributed under the terms of the Creative Commons Attribution 4.0 International license.

10.1128/mBio.03046-19.7 TABLE S1 Peptide list. Peptides from the I-domain peptide library screen, the biotin-labelled G2 peptide, and two control peptides are listed. For the biotinylated G2 peptide, Ahx is a six-carbon spacer. Control P1 and P2 are peptides from an unrelated gonococcal protein called AniA. They have similar solubility to G2 peptide and were included as a non-I-domain peptide control. Download Table S1, DOCX file, 0.01 MB. Copyright © 2020 Poole et al. 2020 Poole et al. This content is distributed under the terms of the Creative Commons Attribution 4.0 International license.

Having identified the G2 region as a galactose-binding site, we performed SPR to test whether the G2 peptide exhibited high-affinity galactose-binding activity. Synthetic glycans, galactobiose or methyl-α-d-galactose, were flowed over immobilized biotinylated G2 peptide (biotin-AhxG2) complexed with streptavidin. The reference cell contained streptavidin complexed with biotin. Both galactobiose and methyl-α-d-galactose interacted with the G2 peptide with high affinity (Table 2; Fig. S2); the K_D_ values obtained were similar to values obtained for the rI-domain. As observed for the rI-domain and rCR3, the G2 peptide had no concentration-dependent interaction with sucrose. Thus, these data indicated that high-affinity galactose lectin activity occurs through the G2 peptide region of the CR3 I-domain and, further, that the G2 peptide can recapitulate this lectin activity.

### Modeling of the G2 peptide region.

**FIG 2:**
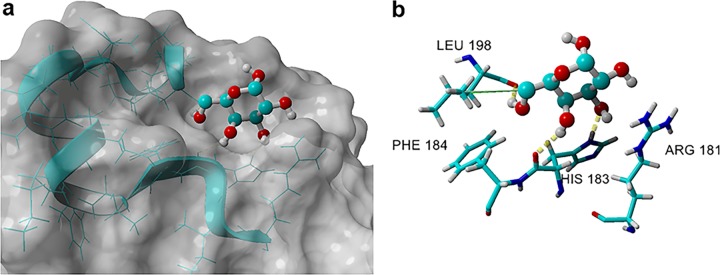
Galactose-α-OH in the preferred cluster, A1, when docked into the G2 region of the human I-domain. (a) The G2 region is shown as the blue ribbon. (b) Amino acids within the G2 region of the I-domain that engage in the major interactions are marked.

The above data showed that the G2 peptide can recapitulate the galactose-specific lectin activity of the CR3 I-domain. Using the G2 region (amino acids 181 to 196 in the X-ray crystal, Protein Data Bank identifier [ID] 1MF7 [34]) as a starting point, we conducted molecular docking studies using galactose to identify ligand-receptor pairs. We found four distinct clusters, A1, B1, C1, and D1, after 999 dockings of potentially bound galactose-α-OH conformations in the G2-region (see Fig. S4). The most preferred cluster, A1, had the highest binding energy (3.955 kcal/mol) (35) and the highest percentage (70%) of populated ligand conformations (Fig. S4). Galactose-α-OH bound to the main A1 cluster within the G2 peptide region (Fig. 2a) and engaged His183 through two hydrogen bonds (OH-3 and OH-4) (Fig. 2b). Leu198 also interacts with galactose-α-OH in the form of a hydrogen bond with OH-6 plus a hydrophobic interaction with the CH_2_ group at C-6. Galactose-α-OH is further flanked and stabilized by Phe184 and Arg181. Overall, cluster A1 represents a galactose-binding pocket.

10.1128/mBio.03046-19.4 FIG S4 AutoDock Vina experiment of galactose-α-OH. Molecular docking identified four possible galactose-binding sites within the area of the G2 peptide. (a) AutoDock Vina experiment of galactose-α-OH into the G2 region (marked in red) of the human CR3 I-domain results in 4 distinct clusters: A1, B1, C1, and D1, with a ligand population of 70%, 10%, 15%, and 5%, respectively. (b) A total of 999 runs were docked. PDB structure 1MF7 was used for docking studies using AutoDock Vina implemented in the YASARA software suit. More-positive energies indicate stronger binding. Percentages of the populated docked structures are also shown, indicating that cluster A1, which includes the G2 peptide region, is the preferred binding galactose-α-OH conformation. Download FIG S4, PDF file, 0.05 MB. Copyright © 2020 Poole et al. 2020 Poole et al. This content is distributed under the terms of the Creative Commons Attribution 4.0 International license.

### Gonococcal adherence to primary cervical cells can be blocked by peptide G2.

**FIG 3:**
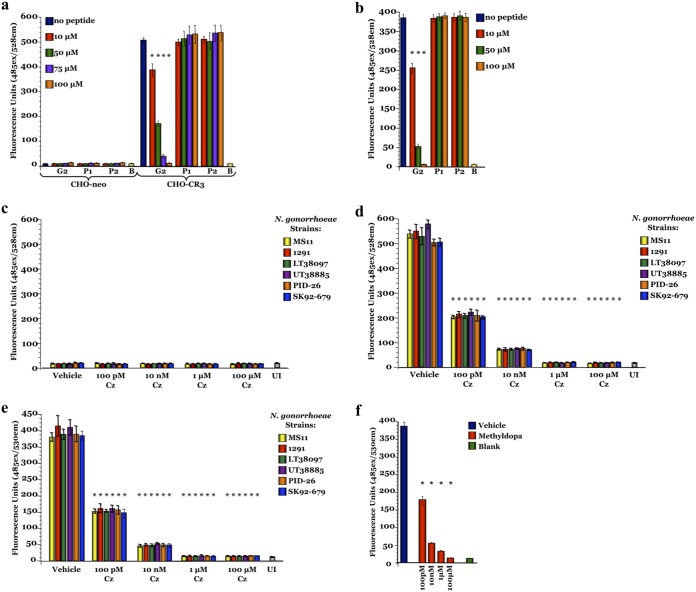
Blocking the I-domain prohibits N. gonorrhoeae adherence to CR3-expressing host cells. Fluorometric adherence assays were performed as described in the text using CHO-neo, CHO-CR3, or Pex cells. Fluorescence (*y* axis), indicative of adherence, was recorded following a 1-h infection with GFP-expressing N. gonorrhoeae strains MS11 *gfp*, 1291 *gfp*, UT38097 *gfp*, LT38885 *gfp*, PID-26 *gfp*, or SK92-679 *gfp*. A dose-dependent reduction in N. gonorrhoeae adherence occurred when I-domain blocking (G2) but not control (P1 and P2 nonblocking) peptides were included in the infection assay using CR3-expressing CHO-CR3 (a) and Pex (b) cells, but not CHO-neo (a) cells, which do not express CR3. Carbamazepine (Cz) (c to e) and methyldopa (f) also blocked N. gonorrhoeae host cell adherence. A dose-dependent decrease in gonococcal adherence to CHO-CR3 (d) and Pex (e) cells was observed in the presence of carbamazepine. (c) However, carbamazepine had no effect on N. gonorrhoeae adherence to CHO-neo cells, in which only background levels of adherence occurred. (f) Methyldopa similarly resulted in a dose-dependent decrease in N. gonorrhoeae adherence to Pex cells. Each assay was performed in triplicates on 3 separate occasions. Data are presented as the means and variances of the average values obtained for each assay. UI, uninfected cells; B, blank; *, *P* ≤ 0.0001 versus uninfected cells, blank wells, and/or infections performed in the absence of any peptide or drug (all comparisons).

Analysis of cervical biopsy specimens from women with active gonococcal infections showed that more than 92% of N. gonorrhoeae cells are associated with the female uterine cervix via an interaction with CR3 (20). The N. gonorrhoeae-CR3 interaction occurs solely through the I-domain, requires gonococcal pilus (32), and is mediated by the pilin-linked glycan (33). Therefore, to examine the galactose–I-domain interaction in a biological system, we performed fluorometric adherence assays (Fig. 3). Pex cells, as well as CR3-expressing (CHO-CR3) and -nonexpressing (CHO-neo) Chinese hamster ovary (CHO) cells, were seeded onto microtiter plates, and gonococcal adherence was quantitated fluorometrically. MS11 *gfp* adherence to Pex and CHO-CR3 cells decreased in the presence of I-domain blocking (G2) but not control (P1 and P2 nonblocking) peptides (Fig. 3a and b). The decreased gonococcal adherence to Pex and CHO-CR3 cells was not significantly (*P* ≥ 0.45 and *P* ≥ 0.67, respectively) different from that to uninfected cells with the use of 100 μM G2 peptide. Only background fluorescence was recorded for assays performed using CHO-neo cells or uninfected cells and for wells devoid of Pex or CHO cells incubated with MS11 *gfp*. Thus, these data confirmed the lectin function of the CR3 I-domain, and they highlight the importance of the pilin Gal(α1-3)diNAcBac glycan-CR3 I-domain interaction to cervical infection.

### Repurposed drugs that block the pilin glycan–I-domain interaction prevent and cure cervical infection.

Having demonstrated that gonococcal adherence to Pex cells could be inhibited by the G2 peptide, we concluded this interaction is a novel and promising target for therapeutic drugs. A library of 3,141 drugs, small molecules, nutraceuticals, and dyes was screened for binding to the human I-domain by SPR. Initial screening of the compounds at 1 μM identified 30 possible targets (see Fig. S5a and b). These 30 compounds were rescreened for binding affinity and for their ability to block N. gonorrhoeae MS11 wild-type pilin from binding to human rI-domain in direct competition SPR experiments. Only six compounds bound with high affinity to rI-domain and completely blocked the interaction of N. gonorrhoeae MS11 pilin with rCR3 or rI-domain. Two of these blocking compounds met our criteria for current use and safety in humans, carbamazepine (K_D_ = 2.12 nM ± 0.24) and methyldopa (K_D_ = 1.01 nM ± 0.09), which were further evaluated for their potential utility in treating N. gonorrhoeae cervical infection (Fig. S5c and d).

10.1128/mBio.03046-19.5 FIG S5 Identification of carbamazepine and methyldopa in SPR. (a) Carbamazepine identification in SPR screen. Highlighted spot is carbamazepine (CA sample SN00838842; molecular weight, 236 g/mol). (b) Methyldopa identification in SPR screen. Highlighted spot is methyldopa. (c) Single-cycle kinetics curve of carbamazepine against the rI-domain of CD11b surface plasmon resonance assay. Maximum concentration was 10 nM with a 1:2 dilution down to 0.625 nM. (d) Single-cycle kinetics curve of methyldopa against the rI-domain of CD11b surface plasmon resonance assay. Maximum concentration was 10 nM with a 1:2 dilution down to 0.625 nM. Download FIG S5, PDF file, 0.2 MB. Copyright © 2020 Poole et al. 2020 Poole et al. This content is distributed under the terms of the Creative Commons Attribution 4.0 International license.

The ability of carbamazepine and methyldopa to block N. gonorrhoeae adherence to CHO-neo, CHO-CR3, and Pex cells was evaluated using a fluorometric adherence assay (Fig. 3; see also Fig. S6a). Gonococcal adherence to CHO-CR3 and Pex cells, but not to CHO-neo cells, decreased in a dose-dependent manner with the use of either drug, which is consistent with a CR3-dependent mechanism of action.

10.1128/mBio.03046-19.6 FIG S6 The effect of repurposed drugs on N. gonorrhoeae. (a) Fluorometric adherence assays performed using CHO-neo and CHO-CR3 cells show that a CR3- and dose-dependent reduction in N. gonorrhoeae strain MS11 *gfp* adherence occurred in the presence of methyldopa (Md). Fluorescence (*y* axis), indicative of adherence, was recorded following a 1-h infection. Carbamazepine (b) and methyldopa (c) treatments resulted in significant reductions in the numbers of viable gonococci at 24 h posttreatment, which were further reduced by 48 h. Both drugs were more effective than ceftriaxone (Cfx), the current recommended therapy, in treating established Pex cell infections with the noted low-passage-number gonococcal isolates. (d) Well diffusion assays showed that methyldopa had no effect on the N. gonorrhoeae strains tested in the absence of human cells. Each assay was performed in triplicates on 3 separate occasions. Data are presented as the means and variances of the average values obtained for each assay. *, *P* ≤ 0.0001 versus vehicle control; Cip, ciprofloxacin. Download FIG S6, PDF file, 0.2 MB. Copyright © 2020 Poole et al. 2020 Poole et al. This content is distributed under the terms of the Creative Commons Attribution 4.0 International license.

**FIG 4:**
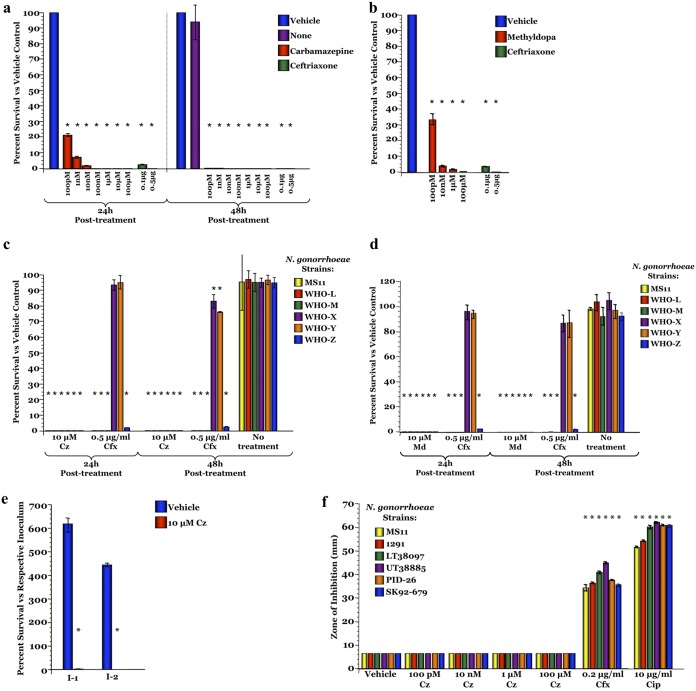
Carbamazepine and methyldopa can cure N. gonorrhoeae infection of human cervical cells. To determine the potential utility of carbamazepine (Cz) and methyldopa (Md) in treating gonococcal cervicitis, Pex cells were infected with the noted strain of N. gonorrhoeae and cure assays were performed. Carbamazepine (a) and methyldopa (b) treatment resulted in dose-dependent reductions in N. gonorrhoeae strain MS11 survival at 24 h posttreatment, which was further reduced by 48 h (b). Carbamazepine (c) and methyldopa (d) were also effective against a panel of multidrug-resistant N. gonorrhoeae isolates. In this regard, both drugs were more effective than ceftriaxone (Cfx), the current recommended therapy, in treating multidrug-resistant gonococci. (e) Bacteria harvested from a 24-h infection (I-1) did not develop carbamazepine-resistance, in that 100% killing occurred when these bacteria were used in a second sequential infection (I-2) and treated with carbamazepine. (f) Well diffusion assays showed that carbamazepine had no effect on the N. gonorrhoeae strains tested in the absence of human cells. Each assay was performed in triplicates on 3 separate occasions. Data are presented as the means and variances of the average values obtained for each assay. *, *P* ≤ 0.0001 versus vehicle control; Cip, ciprofloxacin.

To assess the ability of either drug to cure an established gonococcal infection, Pex cells were challenged with the noted gonococcal strains for 90 min and then treated with carbamazepine, methyldopa, or ceftriaxone. In these treatment assays, less than 99.95% of viable gonococci remained after a 24-h treatment with ≥10 μM carbamazepine or ≥10 μM methyldopa for all strains tested, including multidrug-resistant gonococci (Fig. 4a to d and S6b and c). Killing was mediated by the host cell, as neither carbamazepine nor methyldopa had an effect on gonococcal viability in the absence of Pex cells (Fig. 4f and S6d). To investigate whether the small percentage of those bacteria that survived carbamazepine treatment had developed treatment resistance, sequential infection assays were conducted. Viable bacteria from the colony count plates of a treatment assay were harvested and then used to inoculate new Pex cell monolayers. These sequential assays revealed that the survivor population of bacteria was no more resistant to carbamazepine treatment than the initial inocula; 100% of this survivor population was killed during the second infection (Fig. 4e).

## DISCUSSION

Until recently, carbohydrate-binding activity involving CR3 was attributed to its lectin domain, located within the C-terminal region of CD11b (see Fig. S1 in the supplemental material) (36, 37). The lectin domain functions as a binding site for a wide variety of exogenous polysaccharides, lacks a C-type lectin consensus sequence, and is cation dependent (36, 38, 39) The I-domain, located within the N-terminal region of CD11b, is also reported to have glycan-binding ability, with previous studies demonstrating I-domain lectin activity for glycosylated pili of Neisseria gonorrhoeae strains 1291 and MS11 (33).

Gonococcal pili are phase-variably modified with a monosaccharide or a disaccharide (13, 16). In the previous study (33), pili isolated from wild-type (pilin-linked disaccharide) and *pglA* mutant (pilin-linked monosaccharide) bacteria were shown to bind the CR3 I-domain. Using SPR, we found that a high-affinity (K_D_, 349 nM) interaction with the I-domain occurred for pili with a Gal(α1-3)diNAcBac disaccharide, whereas no concentration-dependent interaction (defined herein as no binding of ≤ 2 μM glycan to rI-domain or rCR3) was recorded for pili isolated from MS11 *pglA* (diNAcBac monosaccharide). These data suggest that, whereas gonococcal pili with both a di- and monosaccharide are capable of binding to the CR3 I-domain, a high-affinity pilin glycan-CR3 I-domain interaction requires the terminal galactose. That is, the diNAcBac present on *pglA* pilin, by itself, was not sufficient to allow a high-affinity interaction with the CR3 I-domain.

The galactose–I-domain interaction observed for wild-type gonococcal pili was not limited to α-galactose. SPR analyses showed that glycans with both α- and β-linked structures formed high-affinity (K_D_ values in the low to mid nanomolar range) interactions with human rI-domain and rCR3. Notably, this galactose-binding activity was limited to the human CR3 I-domain; mouse I-domain was unable to bind any of the glycans tested. Thus, although murine and human I-domain share 77% similarity at the amino acid level, the functional galactose-specific lectin activity that we observed may be limited to humans or higher primates. In this regard, the production (in response to normal flora) of natural anti-galactose antibodies is also limited to higher primates (40). It is accepted that engagement of the CR3 I-domain alone does not trigger a proinflammatory response and, thereby, is thought to confer a survival advantage to invasive organisms that use this mechanism as a means to promote infection. Although speculative, galactose-binding activity of the human, but not the murine, I-domain might suggest that CR3, and specifically, the CR3 I-domain, has evolved to bind and phagocytose resident microorganisms that display galactose on their surfaces without stimulating a proinflammatory response. Consistent with this idea is our finding that 5 of the 15 amino acids in the identified G2 peptide, the galactose-binding region (Fig. S1), of the human I-domain are different in the same region of the murine I-domain.

N. gonorrhoeae is a highly human-adapted pathogen known to subvert numerous host cell functions to ensure successful infection and its continued survival. Moreover, the transformability and genetic plasticity of N. gonorrhoeae have resulted in the rapid emergence of multidrug-resistant and “untreatable” gonococcal strains. We set out to obtain a greater understanding of the molecular mechanism (specificity, affinity, and location) that governs the interaction between gonococcal pilin and the human CR3 I-domain, which is critical to infection of the female cervix. A further goal was to determine whether we could target the gonococcus-CR3 interaction as a potential novel approach to the growing problem of untreatable gonorrhea. To this end, we identified a peptide receptor mimic, G2, which replicates the high-affinity terminal galactose binding activity observed for I-domain and CR3. Both CHO-CR3 and Pex cells were tested, and with both cell types, the G2 peptide displayed dose-dependent blocking. The ability of the G2 peptide to block gonococcal adhesion in these models validates prophylactic and therapeutic strategies targeting CR3 as a novel approach to combat this multidrug-resistant pathogen.

Repurposing existing drugs is the most rapid path to clinical intervention. Carbamazepine and methyldopa are widely prescribed orally administered drugs and are available in mucosal secretions and in serum (∼17 μM for carbamazepine [41, 42] and 1.4 to 11.4 μM for methyldopa [43]) at concentrations above those found to be therapeutically effective in our studies. Our demonstration that these widely prescribed safe drugs can target CR3 to prevent and cure gonococcal infection of primary human cervical cells is a major advance in tackling multidrug-resistant gonococci. This host-factor targeting approach is less likely to lead to the development of resistance and thus may represent a long-term solution to the growing problem of antibiotic-resistant N. gonorrhoeae. Additionally, targeting CR3 may exist as a viable option to treat other antibiotic-resistant human pathogens that use this receptor to initiate infection/disease.

## MATERIALS AND METHODS

### Bacteria and cell cultures.

Deidentified cervical tissues were obtained from the Cooperative Human Tissue Network (Columbus, OH, USA) and used to procured Pex cells as described previously (44). CHO-neo (vector control parent cell) and CHO-CR3 (CR3-expressing) cells (45) were a gift from R. Ingalls (Boston University, Boston, MA, USA) and from L. Schlesinger (Texas Biomedical Research Institute, San Antonio, TX, USA). CHO cells were maintained in Ham’s F12 medium (Gibco, Grand Island, NY, USA) supplemented with 5% fetal bovine serum plus 0.5 mg/ml G418 (both from Gibco).

N. gonorrhoeae strains used in this study included the laboratory strains 1291 and MS11 (46–48), a panel of low-passage-number clinical isolates (strains LT38097, UT38885, PID-26, and SK92-679), and a panel of multidrug-resistant strains (WHO-L, WHO-M, WHO-X [H041], WHO-Y [F89], and WHO-Z [A8806] [49]). Strains 1291 and LT38097 are male urethral isolates, strains MS11 and UT38885 were obtained from women with uncomplicated gonococcal cervicitis, strain PID-26 was obtained from a patient with pelvic inflammatory disease, and strain SK92-679 is a blood isolate from a patient with disseminated infection. Multidrug-resistant N. gonorrhoeae strains were obtained from Public Health England. Green fluorescent protein (GFP)-expressing gonococci were made by transforming the noted strains with the plasmid pCmGFP (GenBank accession number FJ172221) (44, 50). The MS11 *pglA* mutant was originally generated by the insertion of a kanamycin resistance cassette (16, 17, 51, 52) into *pglA* and is described elsewhere (33). For use, bacteria were harvested from overnight (37°C, 5% CO_2_), GC-IsoVitaleX agar plate cultures and enumerated spectrophotometrically, as previously described (44).

### Recombinant proteins.

Recombinant human and mouse I-domain proteins were made and purified as previously described (31). Human rCR3 (integrin α_M_β_2_) was purchased from R&D Systems (Minneapolis, MN, USA).

### Pilin purification.

Pilin was prepared based on previously described methods (53, 54). Bacteria were harvested following overnight growth on GC agar plates and added to 500 μl of 0.15 M ethanolamine (pH 10.7) to help dissociate the pili. Pili were then sheared by vortexing vigorously for 1 min, after which bacterial cells were removed by centrifugation (12,000 × *g*, 15 min). The supernatant, containing pili, was transferred to a new tube and incubated at 56°C for 1 h. This crude pilus preparation was separated on a 4% to 12% polyacrylamide gradient gel. Pilin was isolated by electroelution (100 mA, 30 min) into SDS buffer using a Mini Whole Gel Eluter (Bio-Rad; Gladesville, NSW, Australia). To verify pilin isolation, different mass fractions were collected, and 20 μl of each fraction was analyzed by Western blotting using an anti-pilin antibody (18).

### Surface plasmon resonance.

SPR analyses were performed using a Biacore S200 system (GE Healthcare Life Sciences, Parramatta, NSW, Australia). Samples were analyzed at 25°C in phosphate-buffered saline (PBS) at a flow rate of 10 μl/min and by using single-cycle kinetics. Human rI-domain, mouse rI-domain, and human rCR3 were immobilized onto separate cells of a Series S CM5 sensor chip using an NHS capture kit (both from GE Healthcare Life Sciences) for purified pilin experiments. For experiments in which pilin-glycan-like structures were examined, human rI-domain and rCR3 were immobilized onto separate cells of a CM5 chip. A blank immobilization was used as a control/reference on all chips. MS11 wild-type and MS11 *pglA* pilin were serially diluted from 2 μM to 0.125 μM in PBS. Glycans (Dextra Laboratories, Reading, UK) (Table 2), resembling the gonococcal Gal(α1-3)diNAcBac pilin-linked glycan, were serially diluted from 2 μM to 0.0078 μM in PBS. Affinity data points were taken at 15 s after injection to avoid an artefactual signal resulting from bulk transport. SPR sensorgrams were analyzed using Biacore Evaluation software (GE Healthcare Life Sciences).

### Peptide library construction and screening.

The human I-domain sequence (NP_000623.2, amino acids Gly127 to Ala325 of CD11b) was used to generate a peptide library (Mimotopes; Mulgrave, VIC, Australia). Each peptide comprised 15 amino acids, with a 5-amino-acid sliding window, to give a 10-amino-acid overlap down the sequence, for a total of 40 peptides (see Table S1 in the supplemental material). Competitive SPR (Biacore T200 system; GE Healthcare Life Sciences) was used to screen the blocking potential of the peptide library. To this end, 100 μg/ml of each peptide was mixed with 1.382 μg/ml (2 μM) α1-3 galactobiose and flowed over immobilized human rI-domain and rCR3 on a Series S CM5 chip. Flow cell 1 was a blank immobilization/reference cell. Blocking peptides were defined as those peptides in which the peptide plus glycan response was less than those for the peptide and glycan alone. SPR sensorgrams were analyzed as noted above.

### SPR analysis of the G2 peptide.

To assess the kinetics of the selected peptides, biotinylated versions of each peptide were synthesized. This enabled immobilization via streptavidin onto Series S CM5 sensor chips. Purified biotin-AhxG2 (G2 peptide with a six-carbon spacer linked to biotin) (Table S1), was incubated with streptavidin (Sigma, Castle Hill, NSW, Australia) for 2 h on ice at a 12:1 molar ratio of peptide to protein. Unreacted peptide was removed using a 10-kDa-molecular-weight-cutoff size exclusion column (EMD Millipore, Bayswater, VIC, Australia). Semi-native SDS-PAGE analysis was used to confirm peptide binding to streptavidin. To this end, streptavidin-peptide complexes were added to nonreducing NuPAGE sample buffer (Life Technologies, Scoresby, VIC, Australia). To ensure that peptide-streptavidin complexes were not denatured, heat was not applied before loading onto SDS-polyacrylamide 4% to 12% gradient gels (Life Technologies). A streptavidin-biotin complex was used as a reference control for the molecular weight shift that could be attributed to the peptide. Serially diluted glycans (1 μM to 0.0625 μM) were flowed over sensor chips. Other aspects of SPR analysis were as described above.

### Modeling the G2 peptide region.

All molecular modeling was performed using YASARA (35). A rectangular box with dimensions 31.89 Å by 18.19 Å by 24.13 Å (x, y, and z) was centered on the coordinates of the G2 peptide region (RIHFTFKEFQNNPNP), amino acids Arg 197 to Pro211 of CD11b or 181 to 196 as found in the X-ray crystal (Protein Data Bank ID 1MF7 [34]). The coordinates of galactose-α-OH were taken from YASARA carbohydrate builder and saved as a ligand Protein Data Bank structure. Molecular docking experiments were performed using Autodock VINA (55), as implemented in the YASARA software suit. Calculated ligand-receptor pairs were clustered using a root mean square deviation [RMSD] cutoff of 5.0 Å and ranked according to binding energy, with more positive energies indicating stronger binding and negative energies meaning no binding. The final pose of ligand bound with the protein was selected by giving priority to the binding energy conformation with the largest binding cluster of the total conformers. The results were viewed using YASARA. During the docking process, 999 different conformers were generated in clusters A1, B1, C1, and D1, which were populated at 70%, 10%, 15%, and 5%, respectively (Fig. S3).

### Fluorometric adherence assay.

Fluorometric adherence assays were performed essentially as described previously (36). In brief, N. gonorrhoeae MS11 *gfp* was used to challenge (1 h) Pex or CHO cells simultaneously with peptide or drug competitor. Infected (devoid of peptide or drug) and uninfected (with peptide, drug, or dimethyl sulfoxide [DMSO] vehicle) control cell assays were treated in parallel with competitive peptide/drug inhibition assays. Fluorescence (485 nm excitation, 528 nm emission) intensity, corresponding to bacterial adherence, was recorded using a Synergy HT multimode microplate reader (BioTek Instruments, Winooski, VT, USA). Blank wells, devoid of Pex or CHO cells, were inoculated with bacteria and served as a control for nonspecific binding. Each assay was performed in triplicates on 3 separate occasions. Peptides used are described in Table S1. A nonparametric analysis of variance (ANOVA) was used to determine the statistical significance of the calculated mean of bacterial adherence.

### Repurposed drug screen against human CR3 I-domain.

Human rI-domain was immobilized onto a CM5 sensor chip with a blank control flow cell as described above. A combination of two FDA-approved drug libraries, Microsource-CPOZ (2,400 drugs) and ML Drug (741 drugs), was purchased from Compounds Australia. Each drug was made up to 1 μM in 10% DMSO in a 384-well plate, with a new chip for every plate screened, just before use in the Biacore S200. A single-concentration injection screen (yes/no) binding assay was performed. Binding was determined based on the response unit shift (equal to the molecular weight-corrected response units of the positive-control glycan) of the stability of the binding phase of the dissociation cycle. Binding “hits” were rescreened across a concentration range of 1.6 nM to 1 μM to define the K_D_ of each interaction. For carbamazepine and methyldopa, saturation occurred between 1.6 nM and 8 nM, and so a new concentration range (10 nM to 0.625 nM) was tested. Any drug with a K_D_ of >1 μM was discarded from further analyses. For competitive SPR assays, N. gonorrhoeae pilin, drug, or drug plus pilin were flowed at 1 μM over human rI-domain, and the response units of each interaction were recorded. Any drug that could not compete with pilin for binding (a 2-fold reduction cutoff, 50% of the drug and pilin response units [RU] combined) to the I-domain was also omitted from further analyses. All SPR sensorgrams and result plots described above were analyzed with Biacore S200 evaluation software (GE Healthcare Life Sciences). A literature review was used to evaluate the remaining drugs for known long-term safety in humans and known therapeutic concentrations.

### Infection and treatment assays.

Infection studies were performed as previously described with modification using a multiplicity of infection of 100 (44). To establish infection before treatment, Pex cells were challenged with N. gonorrhoeae for 90 min. The infection medium was then removed, the cells were rinsed thrice, and fresh medium containing 0.1% DMSO (vehicle control), carbamazepine (100 pM to 100 μM), methyldopa (100 pM to 100 μM), or ceftriaxone (0.1 μg/ml or 0.5 μg/ml, positive control) was added. Infections then proceeded for an additional 24 h or 48 h, after which, the infection medium was removed. Pex cell monolayers were subsequently lysed, serial dilutions of the Pex cell lysates were plated, and viable gonococci were enumerated by counting CFU after 48 h of incubation (37°C, 5% CO_2_). For sequential infection assays, viable bacterial colonies that survived a treatment assay were harvested from the enumeration (CFU) plates and used to inoculate new Pex cell monolayers. For these sequential assays, each infection (the original infection or the infection with bacteria that had survived the original infection) proceeded for 24 h. Pex cells were then processed, and bacteria were enumerated, as described above. For all assays, the percentage of N. gonorrhoeae that survived carbamazepine, methyldopa, or ceftriaxone treatment was determined as a function of bacteria that survived DMSO treatment (set to 100%). All assays were performed in triplicates on 3 separate occasions. A nonparametric ANOVA was used to determine the statistical significance of bacterial survival.

### Well diffusion assays.

Well diffusion assays were performed essentially as described previously (56). Briefly, N. gonorrhoeae strains were spread uniformly across the surfaces of GC agar plates at a culture density of 10^7^ bacteria per ml. Wells were then punctured within the agar surface to which carbamazepine or methyldopa (100 pM to 100 μM), 0.2 μg/ml ceftriaxone, 10 μg/ml ciprofloxacin, or 1% DMSO (vehicle control) was added. Following an overnight incubation (37°C, 5% CO_2_), inhibition of N. gonorrhoeae growth was measured as the diameter (in millimeters) of the area of clearing surrounding (and inclusive of) each well on each agar plate, i.e., the zone of inhibition (ZOI). For agar plates in which a ZOI was not visible, data were recorded as the diameter of the well (6 mm). Assays were performed in triplicates on 3 separate occasions. Statistical significance of data obtained was determined using a Student's *t* test.
