# Supplementary material for: Repurposed Drugs That Block the Gonococcus-Complement Receptor 3 Interaction Can Prevent and Cure Gonococcal Infection of Primary Human Cervical Epithelial Cells
Source: mBio. 2020 Mar 3;11(2):e03046-19. doi: 10.1128/mBio.03046-19 (PMC7064771; doi:10.1128/mBio.03046-19)
Supplement: FIG S1 [file mBio.03046-19-sf001.pdf]

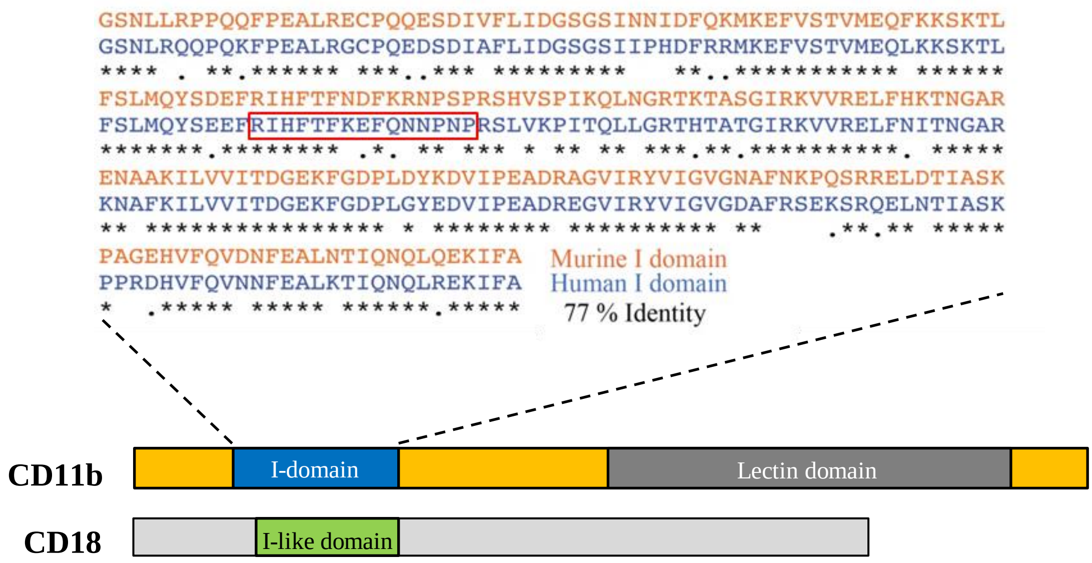
GSNLLRPPQQFPEALRECPQQESDIVFLIDGSGSINNIDFQKMKEFVSTVMEQFKKSKTL
GSNLRQQPQKFPEALRGCPQEDSDIAFLIDGSGSIIPHDFRRMKEFVSTVMEQLKKSKTL
FSLMQYSDEFRIHFTFNDFKRNPSPRSHVSPIKQLNGRTKTASGIRKVVRELFHKTNGAR
FSLMQYSEEFRIHFTFKEFQNNPNPRSLVKPITQLLGRTHTATGIRKVVRELFNITNGAR
ENAAKILVVITDGEKFGDPLDYKDVIPEADRAGVIRYVIGVGNAFNKPQSRRELDTIASK
KNAFKILVVITDGEKFGDPLGYEDVIPEADREGVIRYVIGVGDAFRSEKSRQELNTIASK
PAGEHVFQVDNFEALNTIQNQLQEKIFA
PPRDHVFQVNNFEALKTIQNQLREKIFA
Murine I domain
Human I domain
77 % Identity
CD11b
I-domain
Lectin domain
CD18
I-like domain
