# Supplementary material for: Repurposed Drugs That Block the Gonococcus-Complement Receptor 3 Interaction Can Prevent and Cure Gonococcal Infection of Primary Human Cervical Epithelial Cells
Source: mBio. 2020 Mar 3;11(2):e03046-19. doi: 10.1128/mBio.03046-19 (PMC7064771; doi:10.1128/mBio.03046-19)
Supplement: FIG S2 [file mBio.03046-19-sf002.pdf]

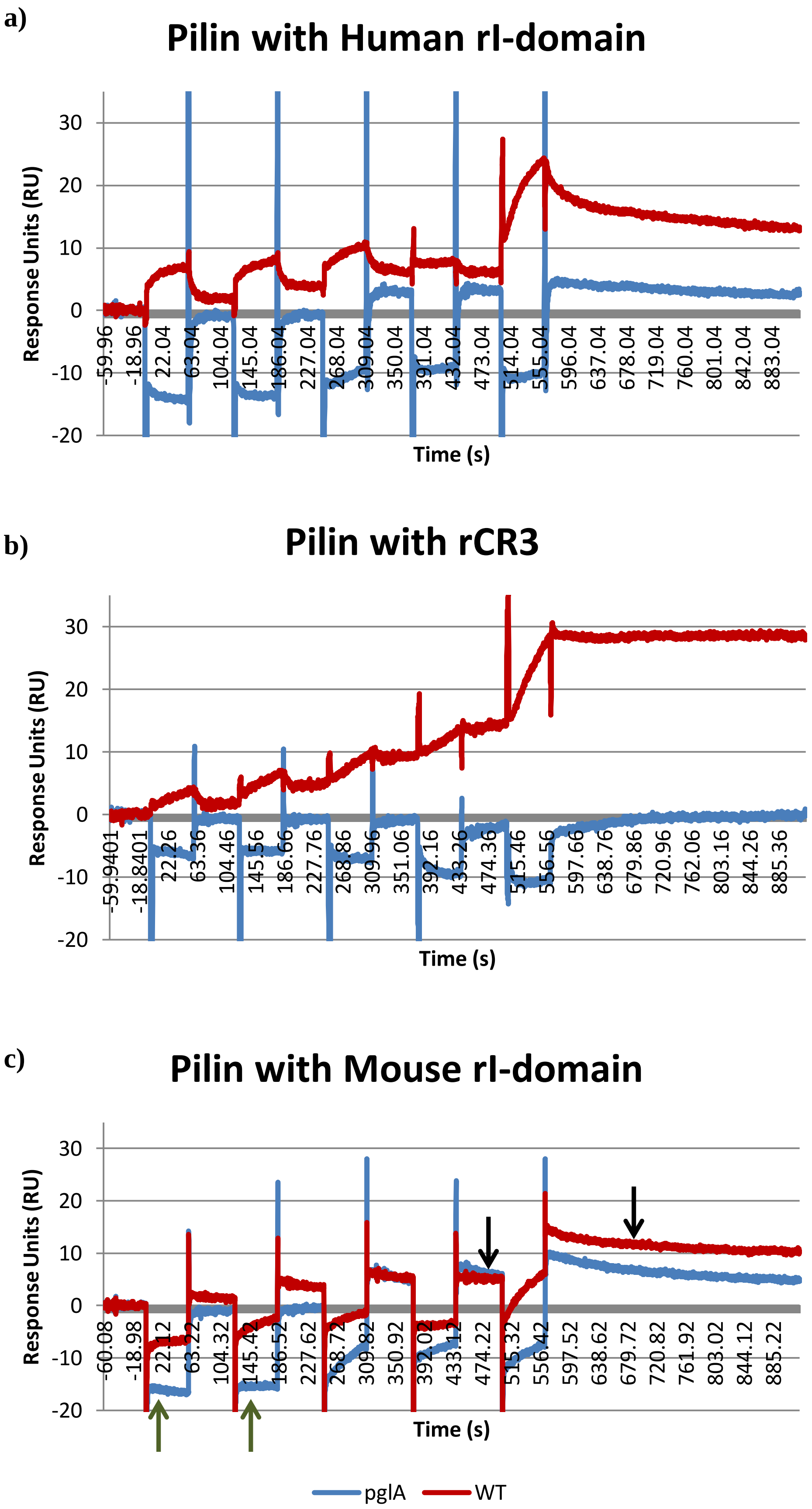
a)
Pilin with Human rI-domain
Response Units (RU)
Time (s)
b)
Pilin with rCR3
Response Units (RU)
Time (s)
c)
Pilin with Mouse rI-domain
Response Units (RU)
Time (s)
pglA
WT

d)

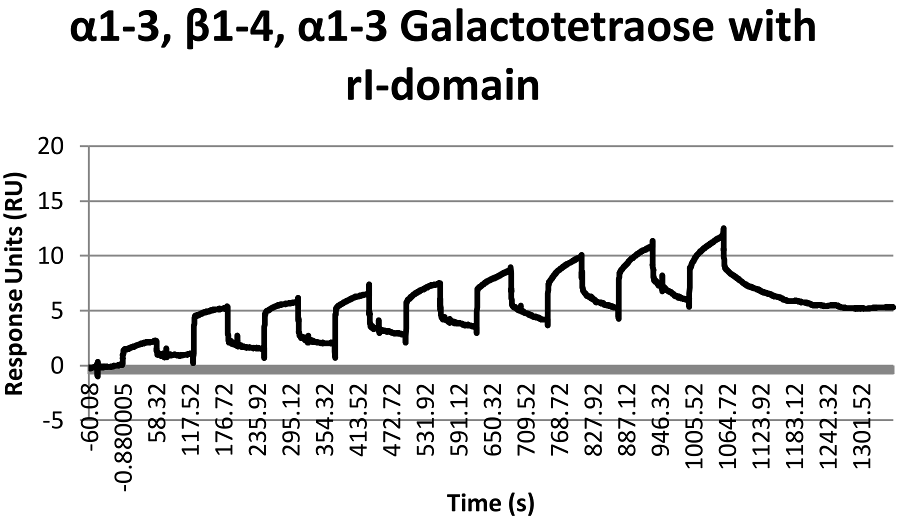
α1-3, β1-4, α1-3 Galactotetraose with rI-domain
Response Units (RU)
20
15
10
5
0
-5
-60.08
-0.880005
58.32
117.52
176.72
235.92
295.12
354.32
413.52
472.72
531.92
591.12
650.32
709.52
768.72
827.92
887.12
946.32
1005.52
1064.72
1123.92
1183.12
1242.32
1301.52
Time (s)

e)

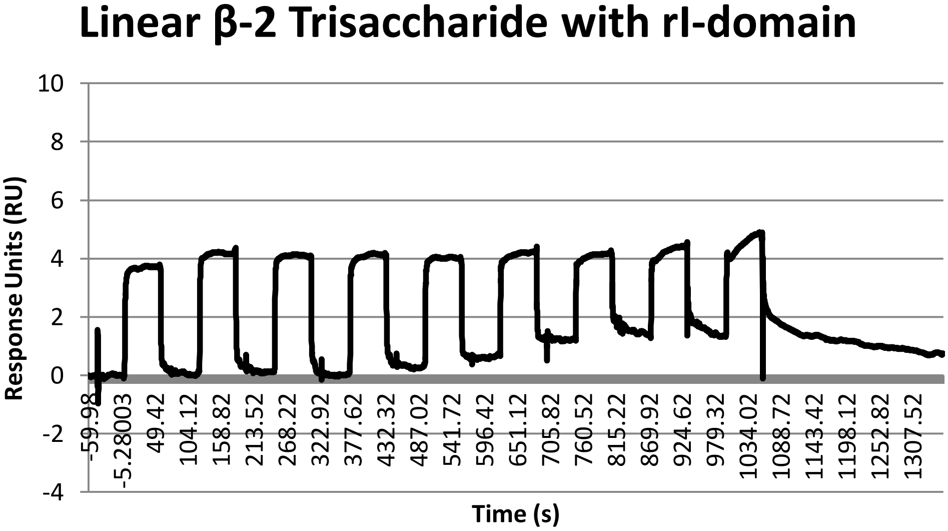
Linear β-2 Trisaccharide with rI-domain
Response Units (RU)
10
8
6
4
2
0
-2
-4
-59.98
-5.28003
49.42
104.12
158.82
213.52
268.22
322.92
377.62
432.32
487.02
541.72
596.42
651.12
705.82
760.52
815.22
869.92
924.62
979.32
1034.02
1088.72
1143.42
1198.12
1252.82
1307.52
Time (s)

f)

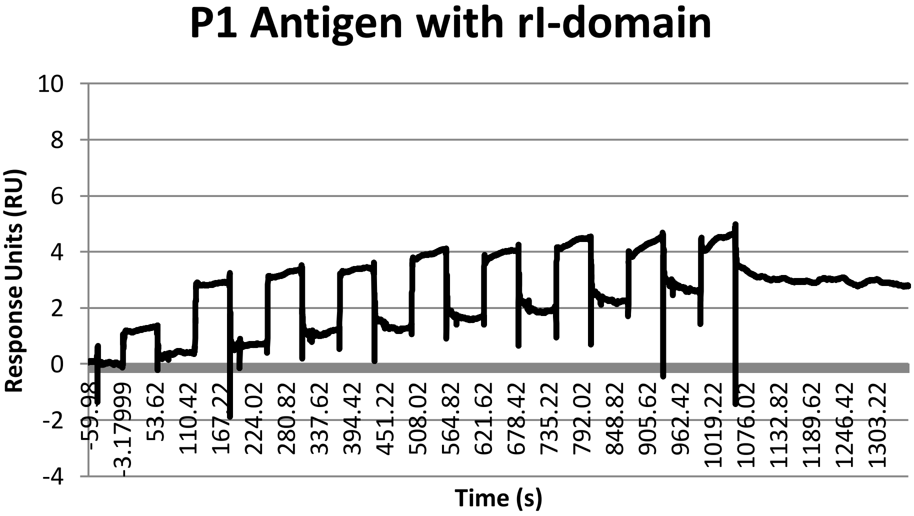
P1 Antigen with rI-domain
Response Units (RU)
10
8
6
4
2
0
-2
-4
-59.98
-3.17999
53.62
110.42
167.22
224.02
280.82
337.62
394.42
451.22
508.02
564.82
621.62
678.42
735.22
792.02
848.82
905.62
962.42
1019.22
1076.02
1132.82
1189.62
1246.42
1303.22
Time (s)

g)

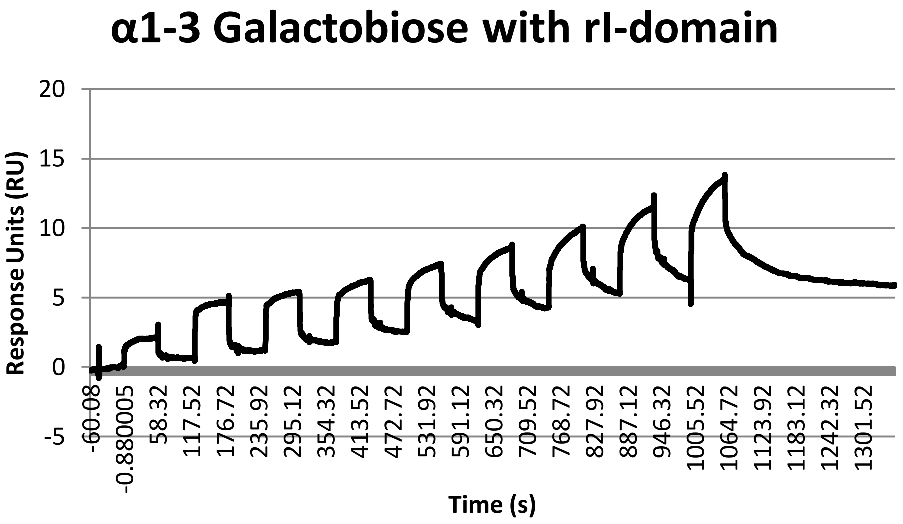
α1-3 Galactobiose with rI-domain
Response Units (RU)
20
15
10
5
0
-5
-60.08
-0.880005
58.32
117.52
176.72
235.92
295.12
354.32
413.52
472.72
531.92
591.12
650.32
709.52
768.72
827.92
887.12
946.32
1005.52
1064.72
1123.92
1183.12
1242.32
1301.52
Time (s)

h)

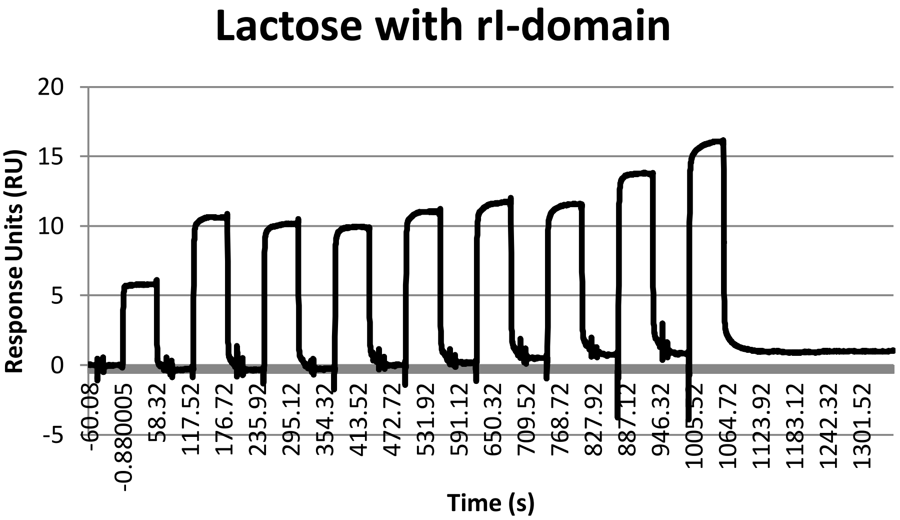
Lactose with rI-domain
Response Units (RU)
20
15
10
5
0
-5
-60.08
-0.880005
58.32
117.52
176.72
235.92
295.12
354.32
413.52
472.72
531.92
591.12
650.32
709.52
768.72
827.92
887.12
946.32
1005.52
1064.72
1123.92
1183.12
1242.32
1301.52
Time (s)

i)

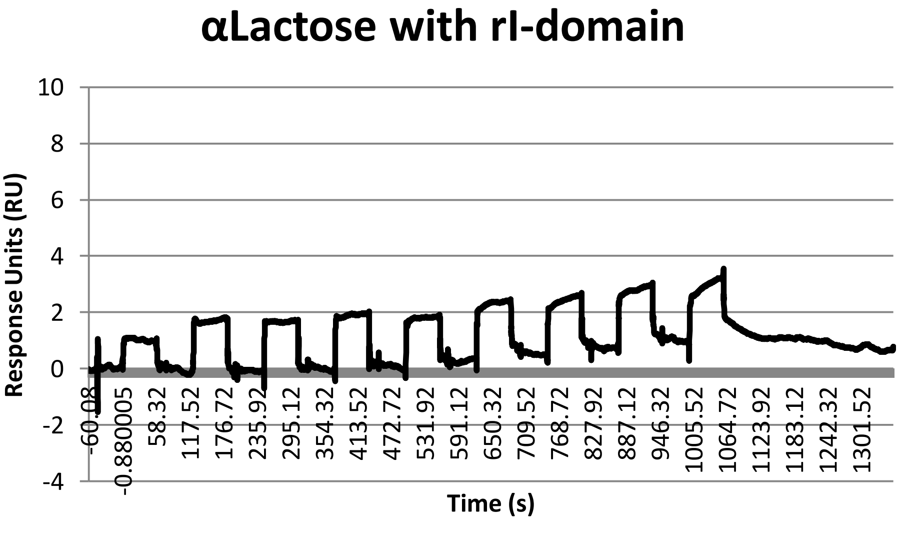
αLactose with rI-domain
Response Units (RU)
10
8
6
4
2
0
-2
-4
-60.08
-0.880005
58.32
117.52
176.72
235.92
295.12
354.32
413.52
472.72
531.92
591.12
650.32
709.52
768.72
827.92
887.12
946.32
1005.52
1064.72
1123.92
1183.12
1242.32
1301.52
Time (s)

j)

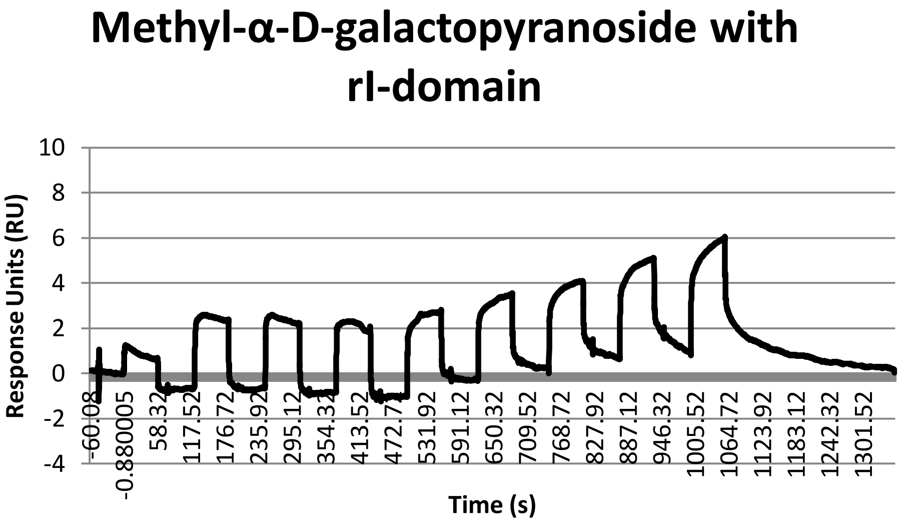
Methyl-α-D-galactopyranoside with rI-domain
Response Units (RU)
10
8
6
4
2
0
-2
-4
-60.08
-0.880005
58.32
117.52
176.72
235.92
295.12
354.32
413.52
472.7
531.92
591.12
650.32
709.52
768.72
827.92
887.12
946.32
1005.52
1064.72
1123.92
1183.12
1242.32
1301.52
Time (s)

k)

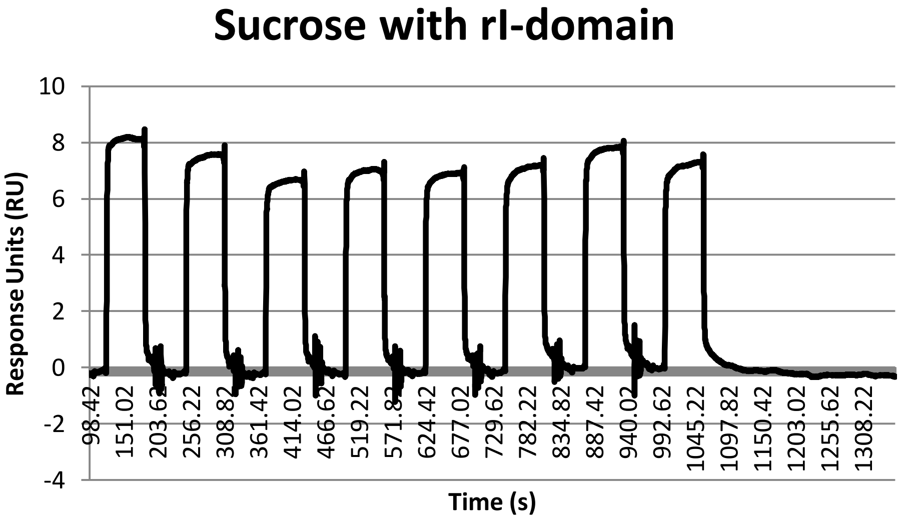
Sucrose with rI-domain
Response Units (RU)
10
8
6
4
2
0
-2
-4
98.42
151.02
203.62
256.22
308.82
361.42
414.02
466.62
519.22
624.42
677.02
729.62
782.22
834.82
887.42
940.02
992.62
1045.22
1097.82
1150.42
1203.02
1255.62
1308.22
Time (s)

l)

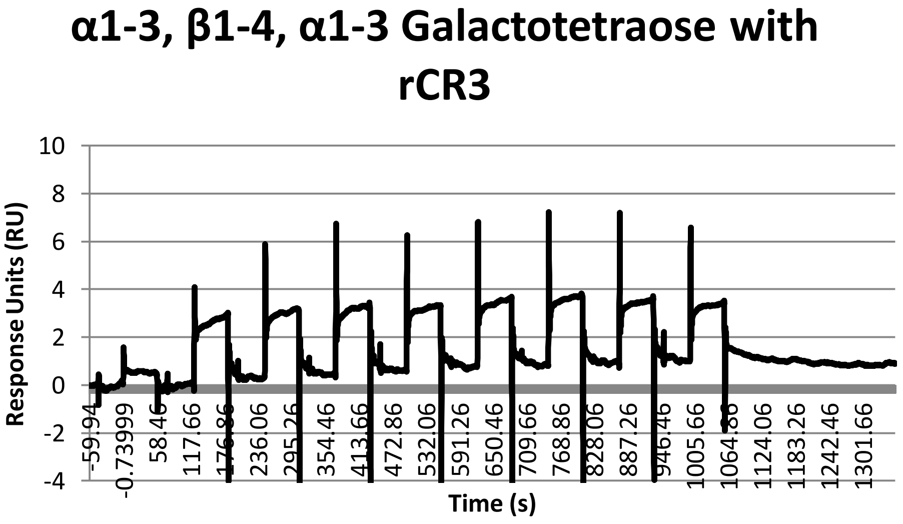
α1-3, β1-4, α1-3 Galactotetraose with rCR3
Response Units (RU)
10
8
6
4
2
0
-2
-4
-59.94
-0.73999
58.46
117.66
236.06
354.46
472.86
532.06
591.26
650.46
709.66
768.86
887.26
1005.66
1124.06
1183.26
1242.46
1301.66
Time (s)

m)

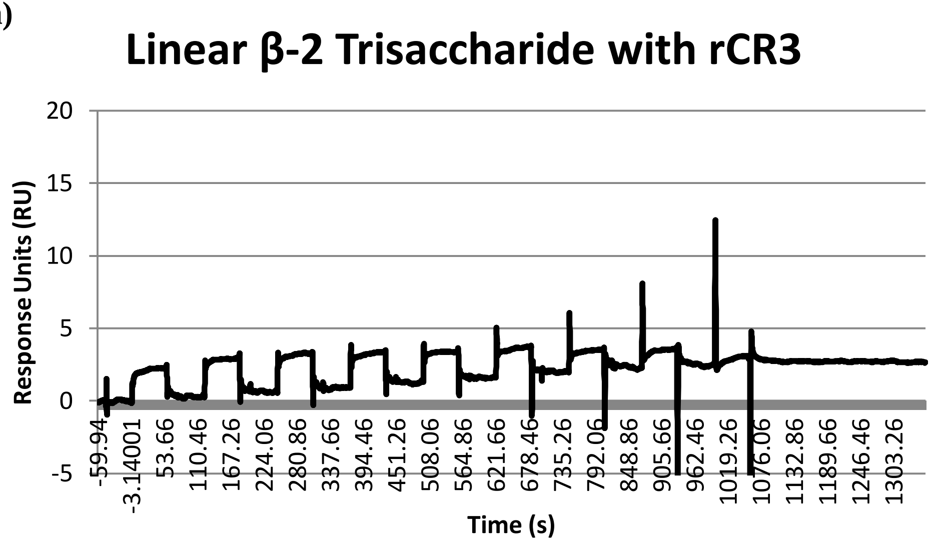
Linear β-2 Trisaccharide with rCR3
Response Units (RU)
20
15
10
5
0
-5
-59.94
-3.14001
53.66
110.46
167.26
224.06
280.86
337.66
394.46
451.26
508.06
564.86
621.66
678.46
735.26
792.06
848.86
905.66
962.46
1019.26
1076.06
1132.86
1189.66
1246.46
1303.26
Time (s)

n)

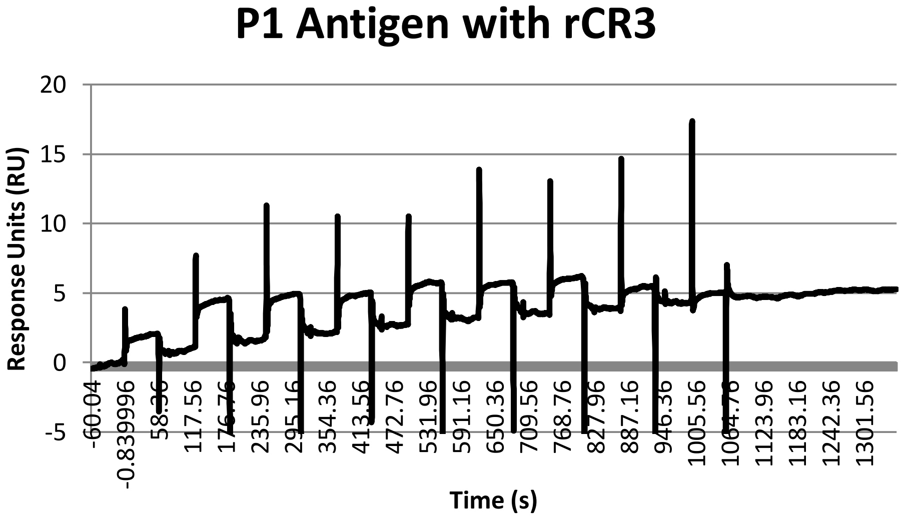
P1 Antigen with rCR3
Response Units (RU)
20
15
10
5
0
-5
-60.04
-0.839996
58.36
117.56
176.76
235.96
295.16
354.36
413.56
472.76
531.96
591.16
650.36
709.56
768.76
827.96
887.16
946.36
1005.56
1064.76
1123.96
1183.16
1242.36
1301.56
Time (s)

o)

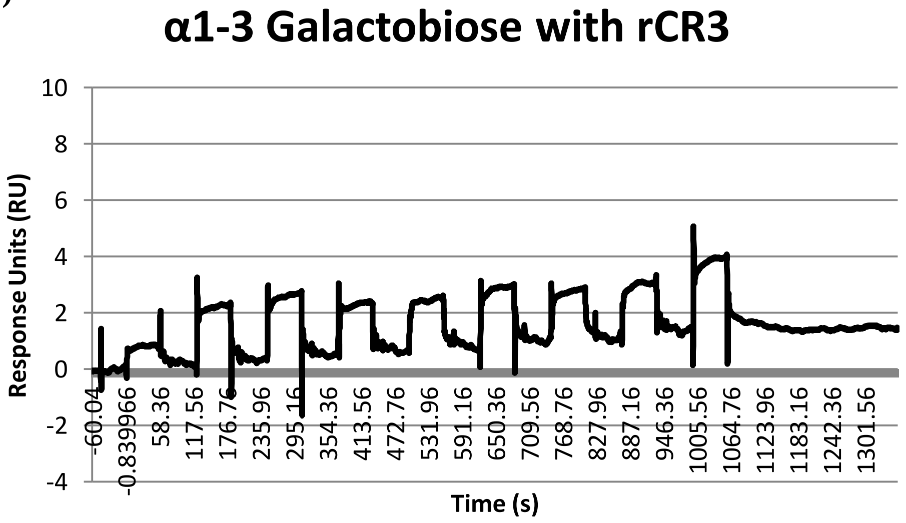
α1-3 Galactobiose with rCR3
Response Units (RU)
10
8
6
4
2
0
-2
-4
-60.04
-0.839966
58.36
117.56
176.76
235.96
295.16
354.36
413.56
472.76
531.96
591.16
650.36
709.56
768.76
827.96
887.16
946.36
1005.56
1064.76
1123.96
1183.16
1242.36
1301.56
Time (s)

p)

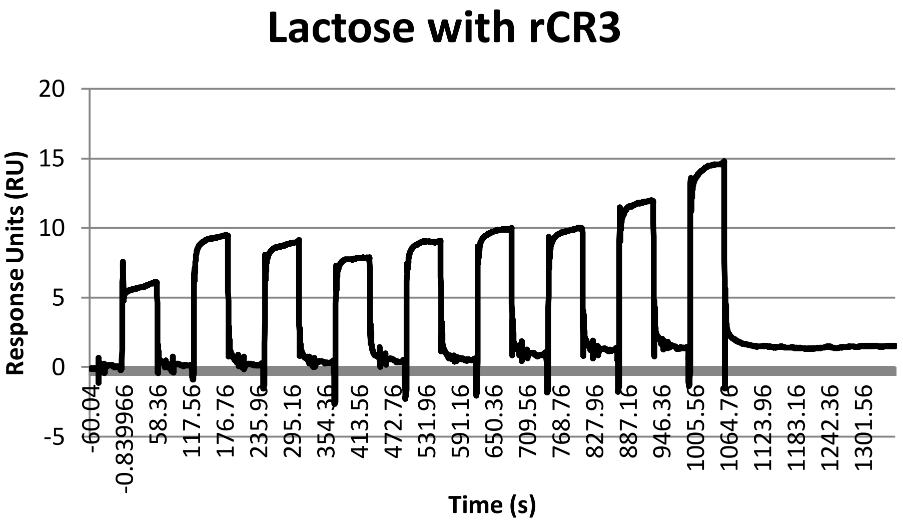
Lactose with rCR3
Response Units (RU)
20
15
10
5
0
-5
-60.04
-0.839966
58.36
117.56
176.76
235.96
295.16
354.36
413.56
472.76
531.96
591.16
650.36
709.56
768.76
827.96
887.16
946.36
1005.56
1064.76
1123.96
1183.16
1242.36
1301.56
Time (s)

q)

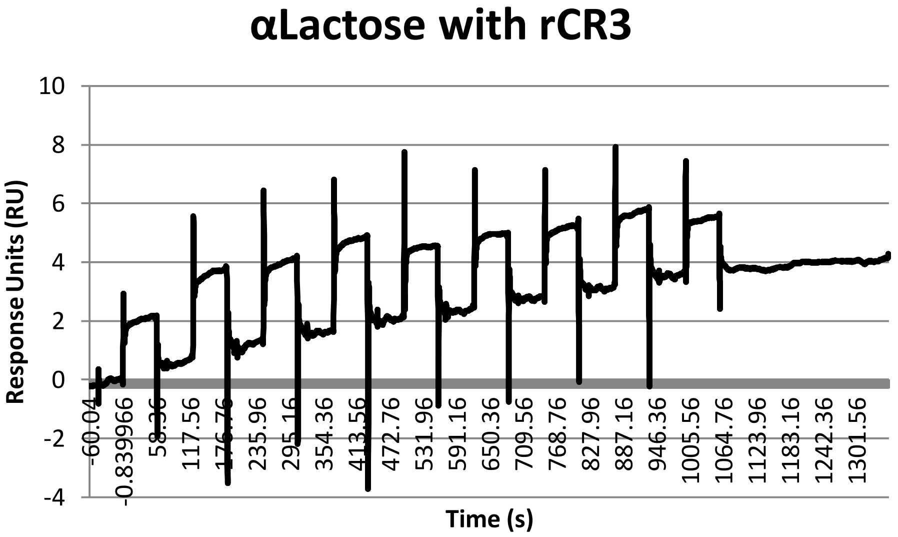
αLactose with rCR3
Response Units (RU)
10
8
6
4
2
0
-2
-4
-60.04
-0.839966
58.36
117.56
176.76
235.96
295.16
354.36
413.56
472.76
531.96
591.16
650.36
709.56
768.76
827.96
887.16
946.36
1005.56
1064.76
1123.96
1183.16
1242.36
1301.56
Time (s)

r)

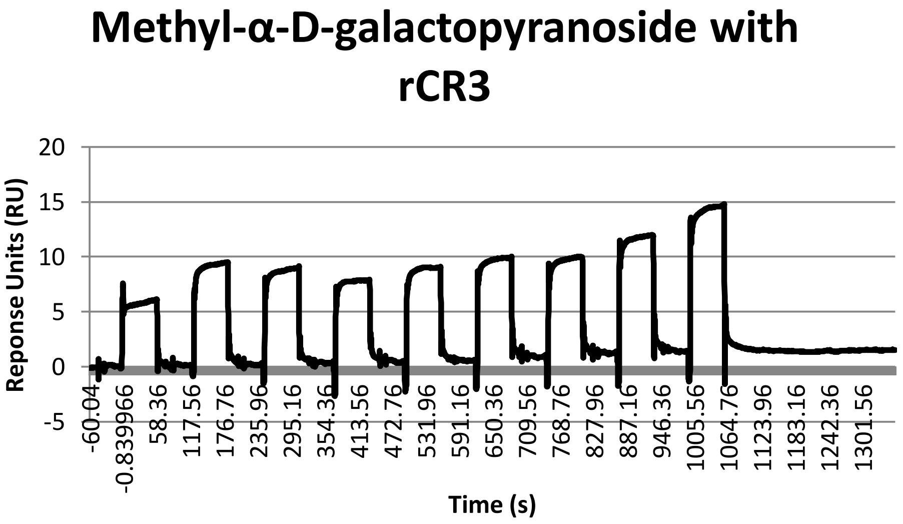
Methyl-α-D-galactopyranoside with rCR3
Reponse Units (RU)
20
15
10
5
0
-5
-60.04
-0.839966
58.36
117.56
176.76
235.96
295.16
354.36
413.56
472.76
531.96
591.16
650.36
709.56
768.76
827.96
887.16
946.36
1005.56
1064.76
1123.96
1183.16
1242.36
1301.56
Time (s)

s)

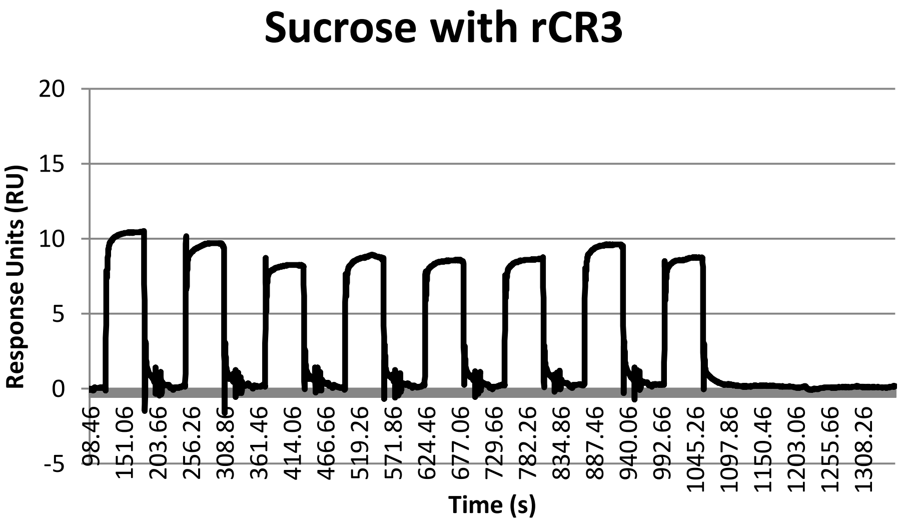
Sucrose with rCR3
Response Units (RU)
20
15
10
5
0
-5
98.46
151.06
203.66
256.26
308.86
361.46
414.06
466.66
519.26
571.86
624.46
677.06
729.66
782.26
834.86
887.46
940.06
992.66
1045.26
1097.86
1150.46
1203.06
1255.66
1308.26
Time (s)

t)

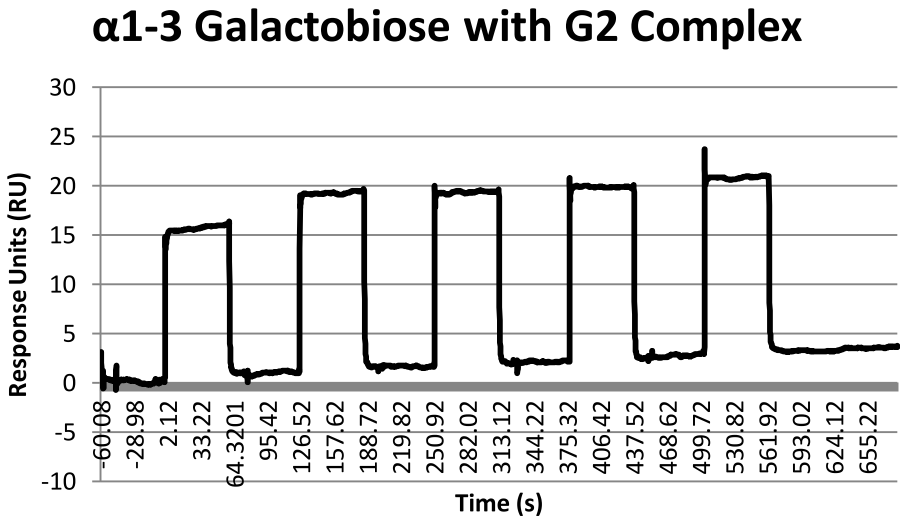
α1-3 Galactobiose with G2 Complex
Response Units (RU)
30
25
20
15
10
5
0
-5
-10
-60.08
-28.98
2.12
33.22
64.3201
95.42
126.52
157.62
188.72
219.82
250.92
282.02
313.12
344.22
375.32
406.42
437.52
468.62
499.72
530.82
561.92
593.02
624.12
655.22
Time (s)

u)

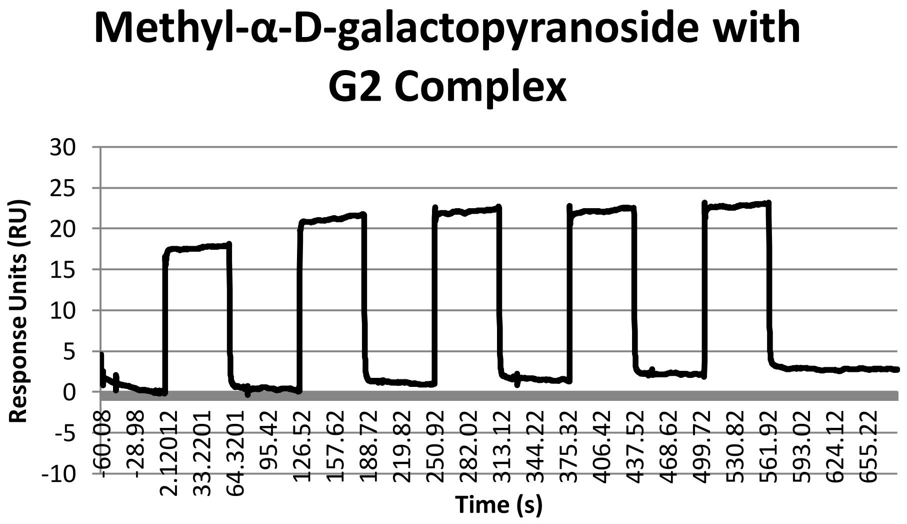
Methyl-α-D-galactopyranoside with G2 Complex
Response Units (RU)
30
25
20
15
10
5
0
-5
-10
-60.08
-28.98
2.12012
33.2201
64.3201
95.42
126.52
157.62
188.72
219.82
250.92
282.02
313.12
344.22
375.32
406.42
437.52
468.62
499.72
530.82
561.92
593.02
624.12
655.22
Time (s)

v)

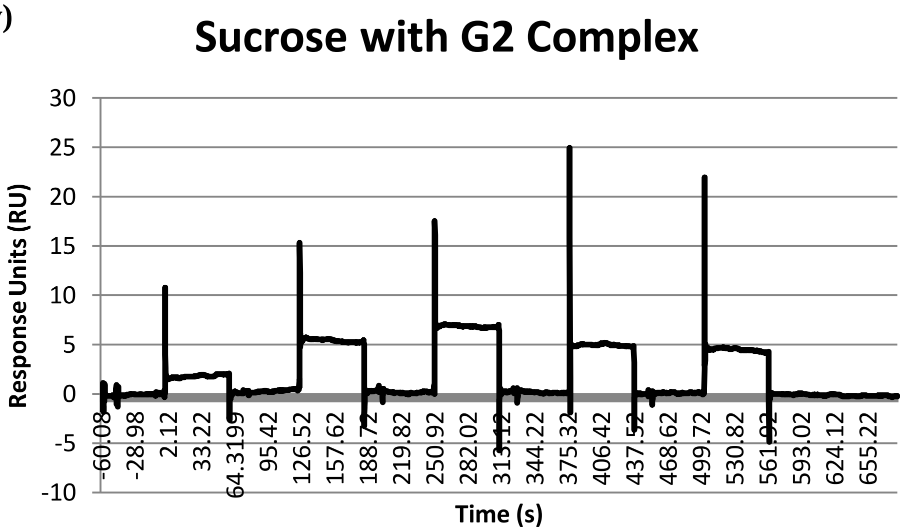

Sucrose with G2 Complex
30
25
20
15
10
5
0
-5
-10
Response Units (RU)
-60.08
-28.98
2.12
33.22
64.3199
95.42
126.52
157.62
188.72
219.82
250.92
282.02
313.12
344.22
375.32
406.42
437.52
468.62
499.72
530.82
561.92
593.02
624.12
655.22
Time (s)
