# Supplementary figures and images for: Repurposed Drugs That Block the Gonococcus-Complement Receptor 3 Interaction Can Prevent and Cure Gonococcal Infection of Primary Human Cervical Epithelial Cells
Source: mBio. 2020 Mar 3;11(2):e03046-19. doi: 10.1128/mBio.03046-19 (PMC7064771; doi:10.1128/mBio.03046-19)

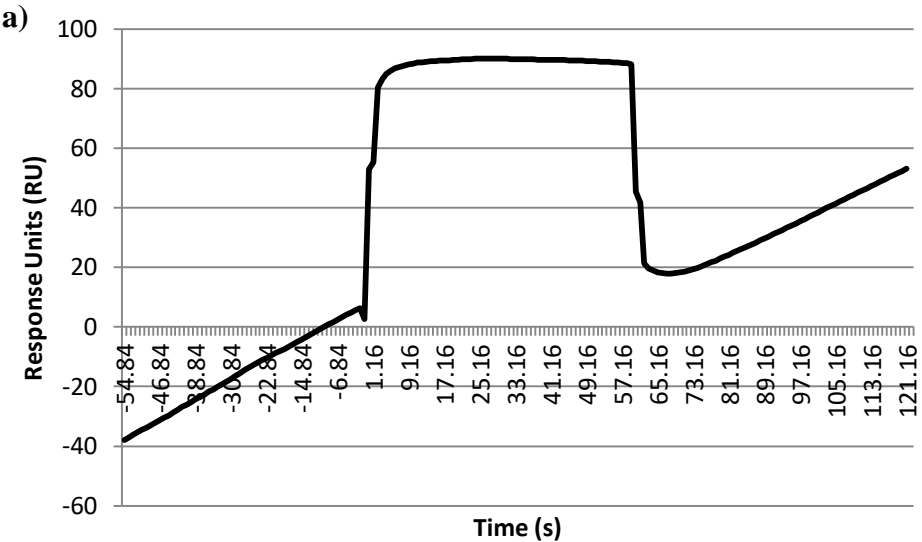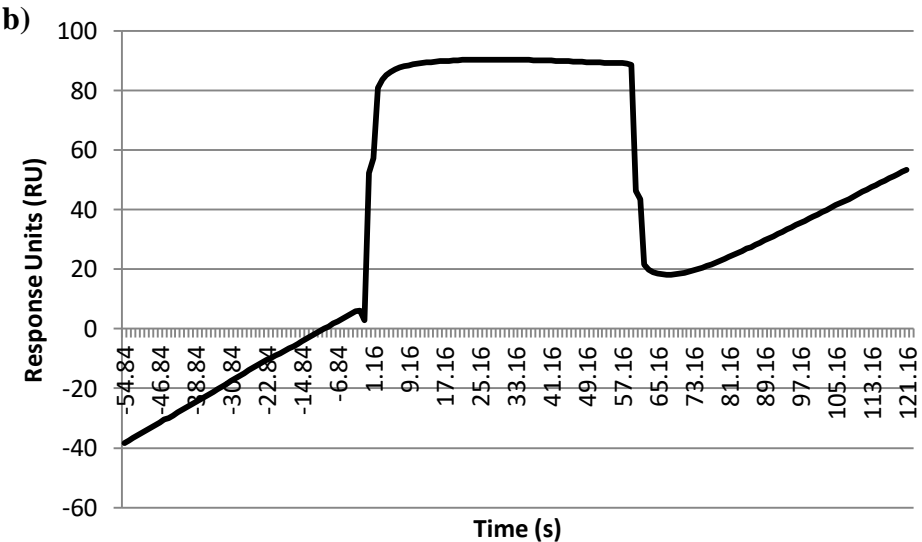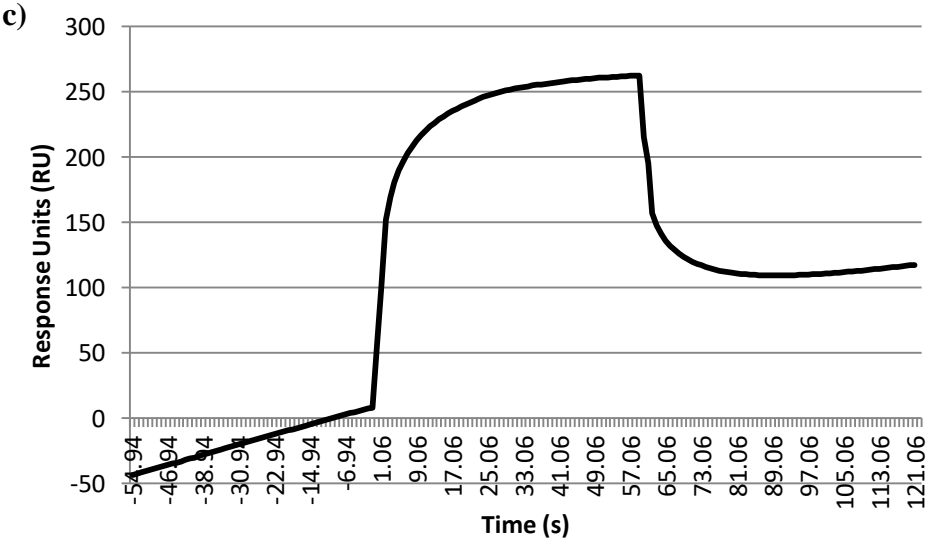

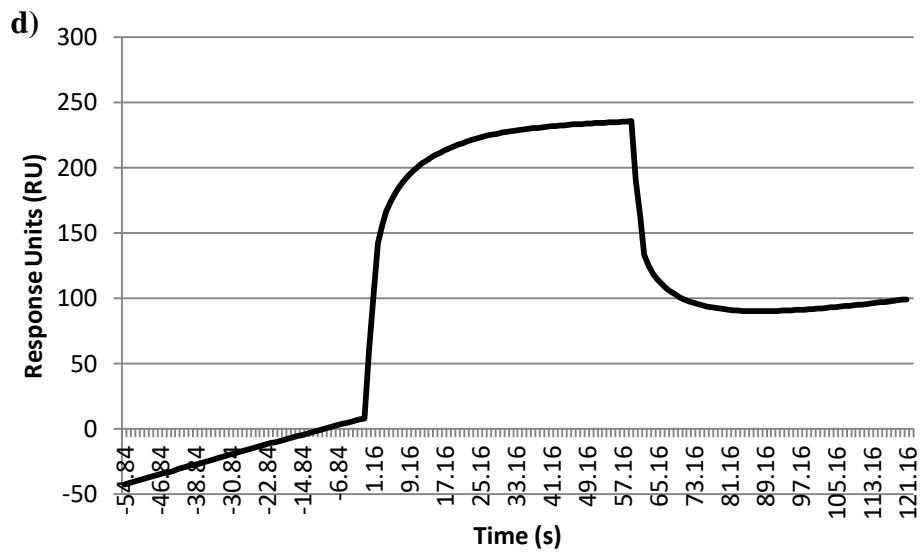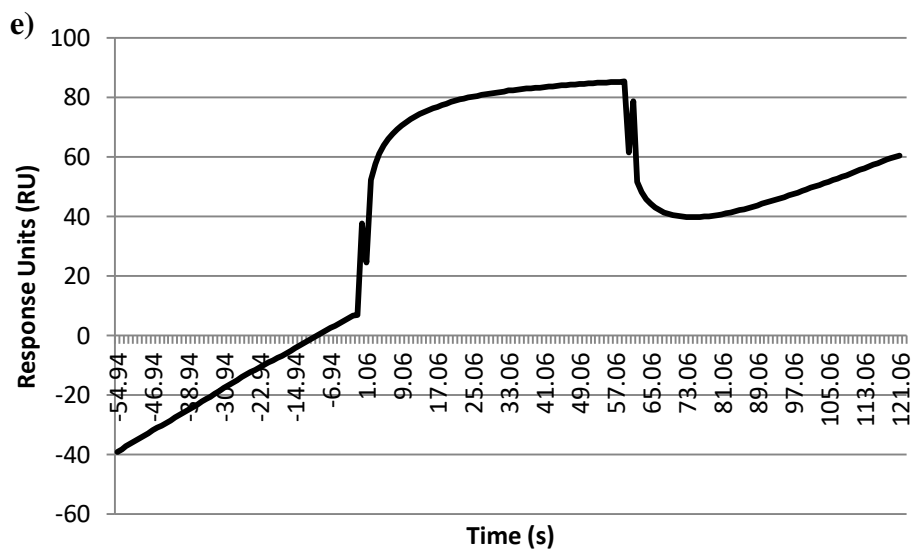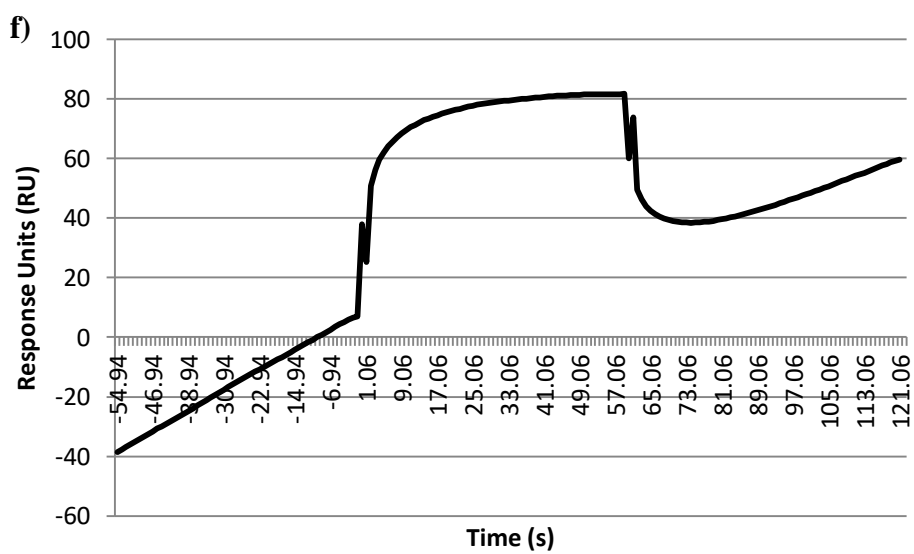

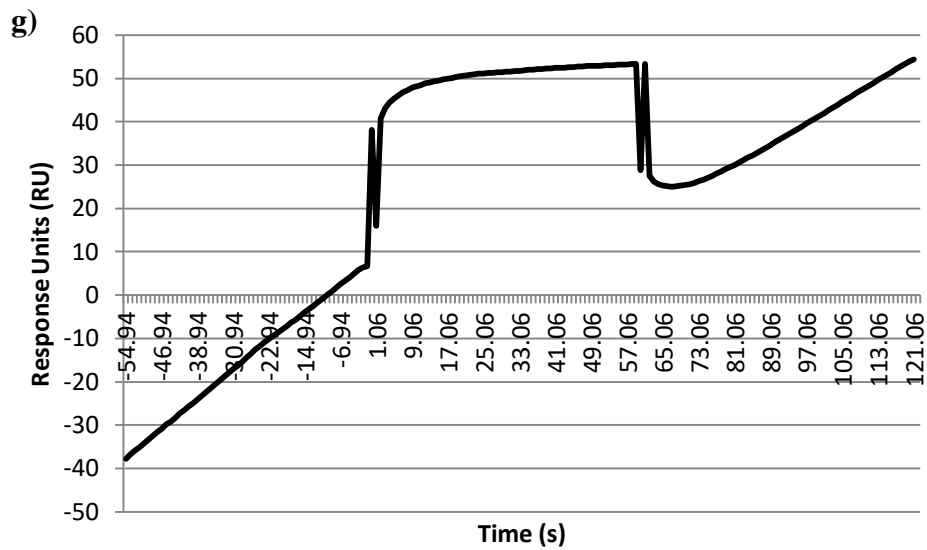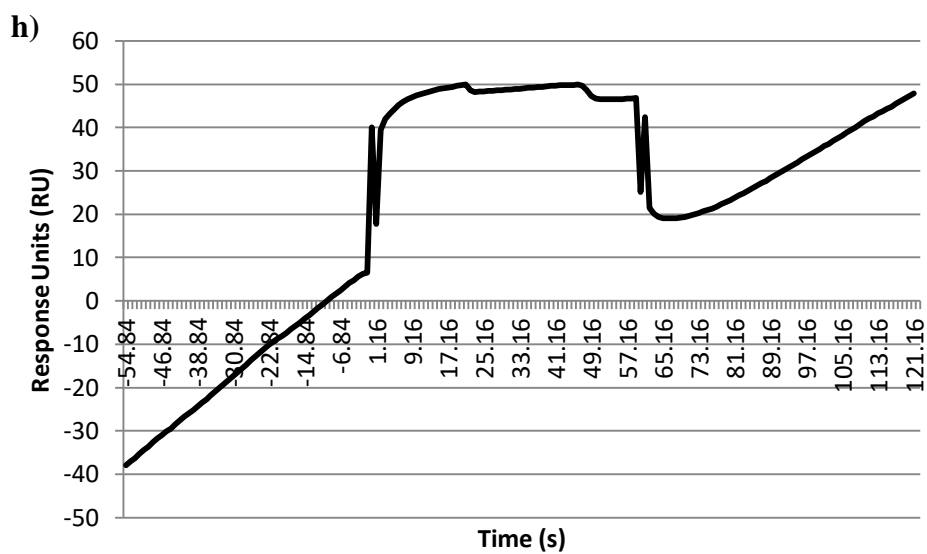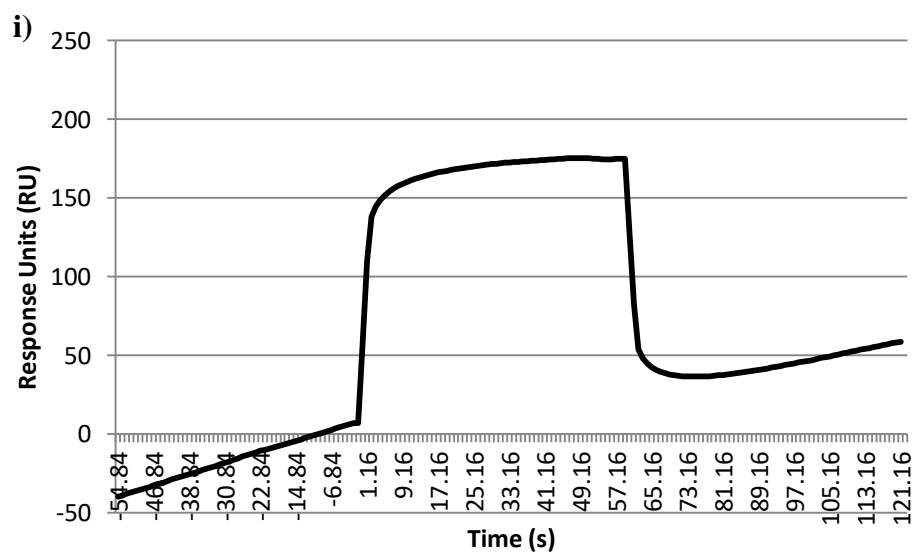

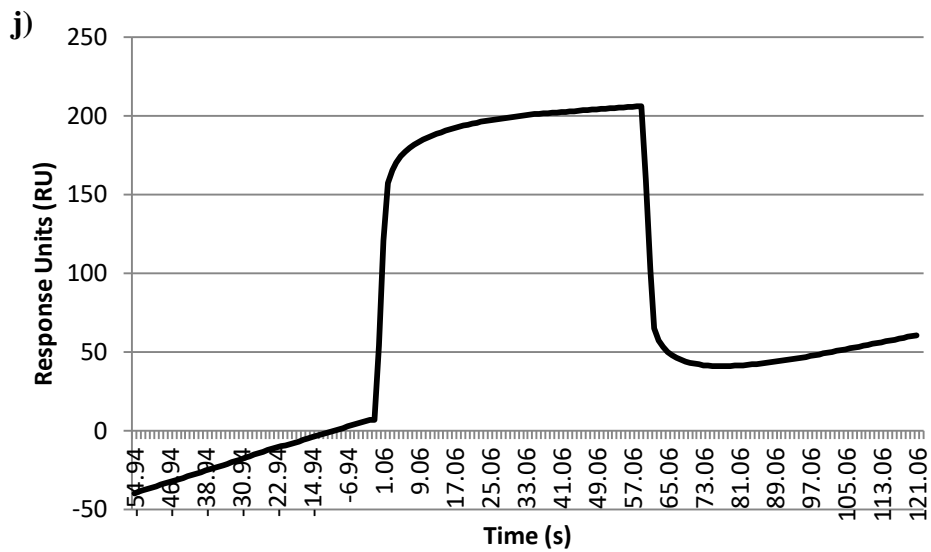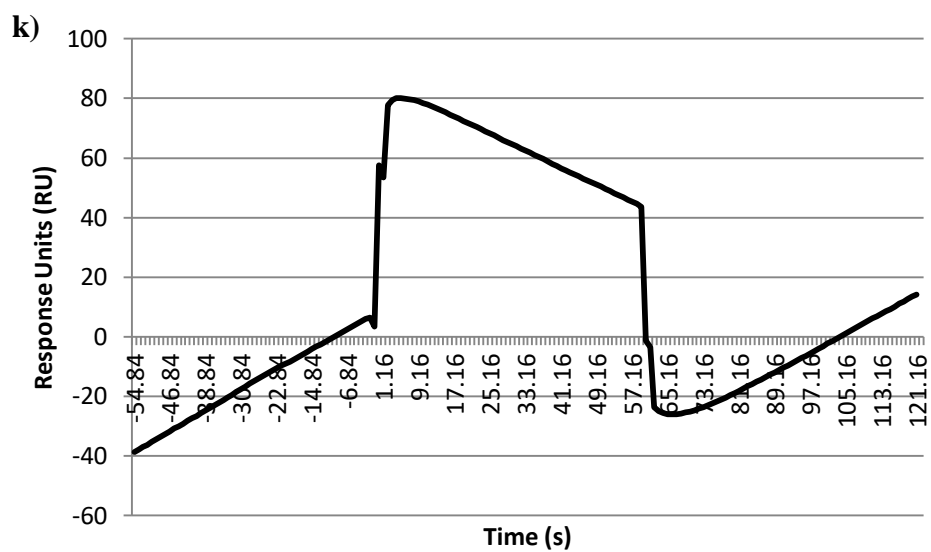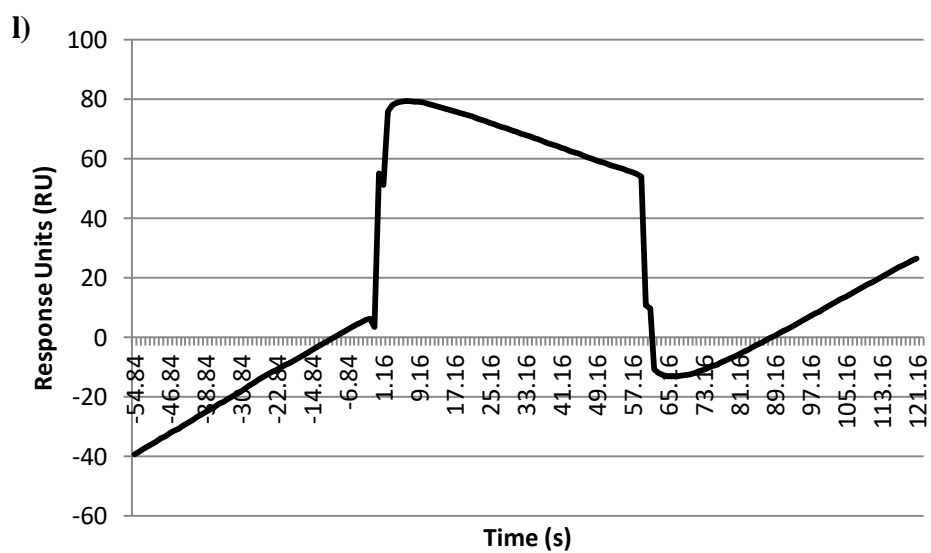

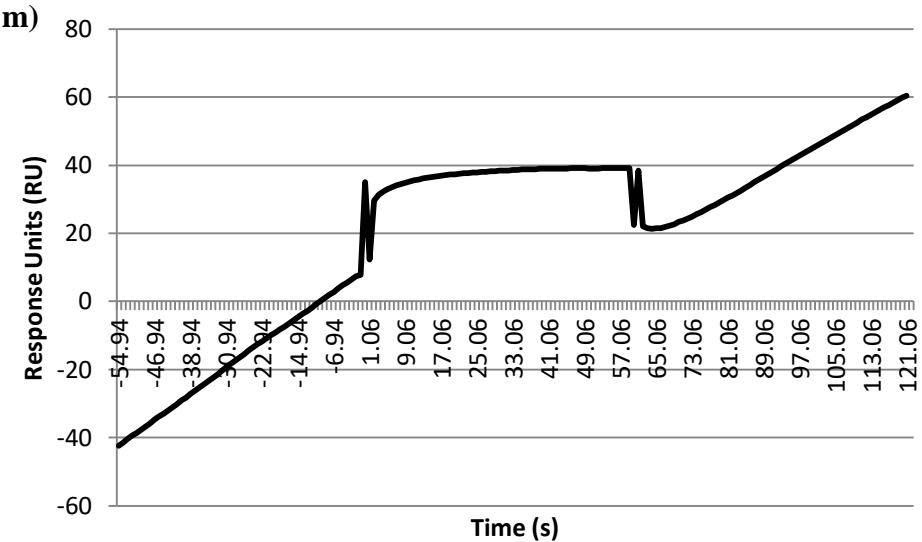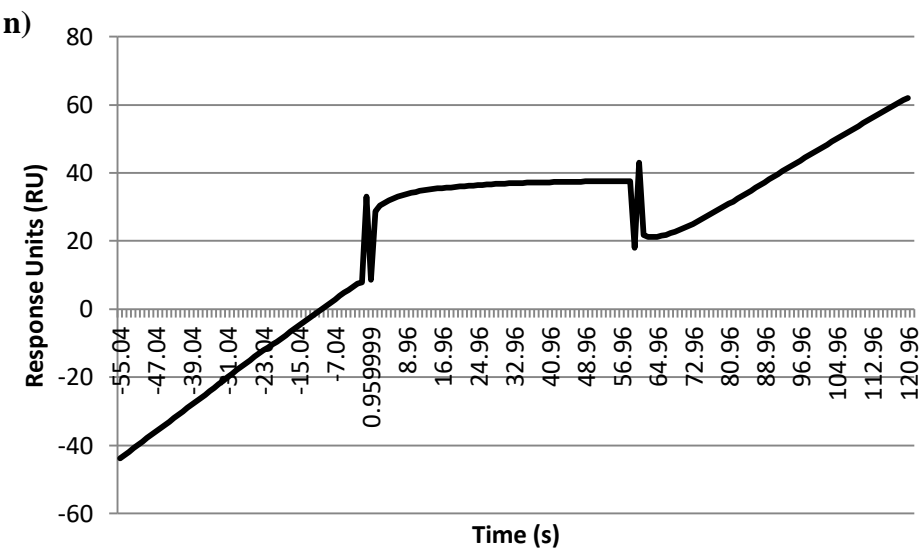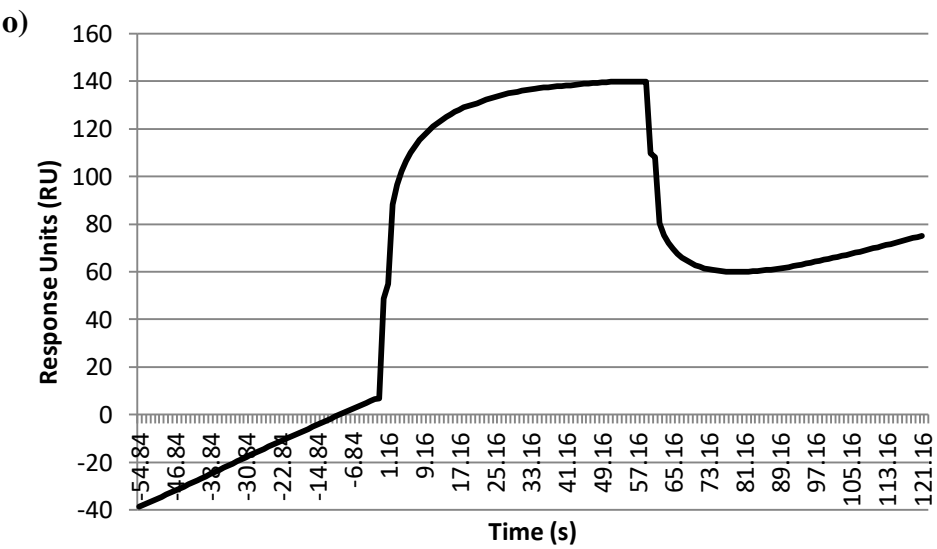

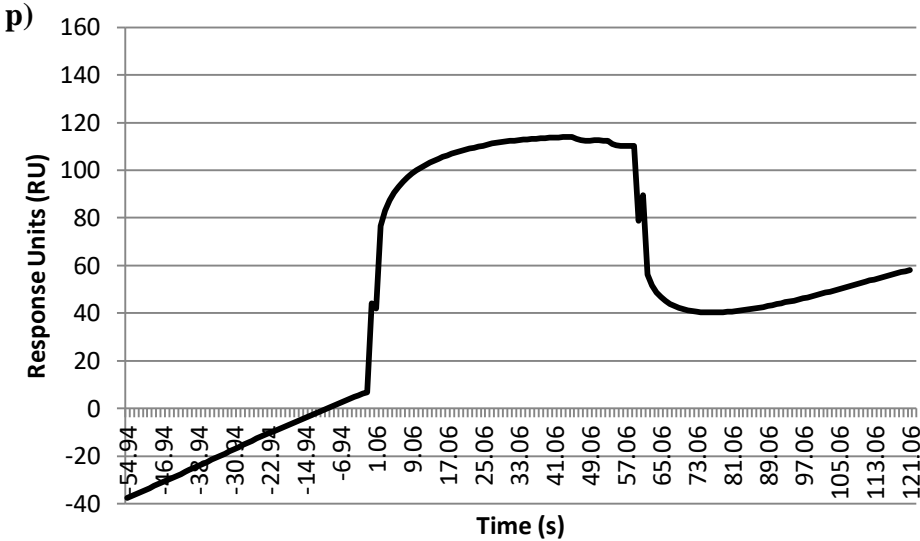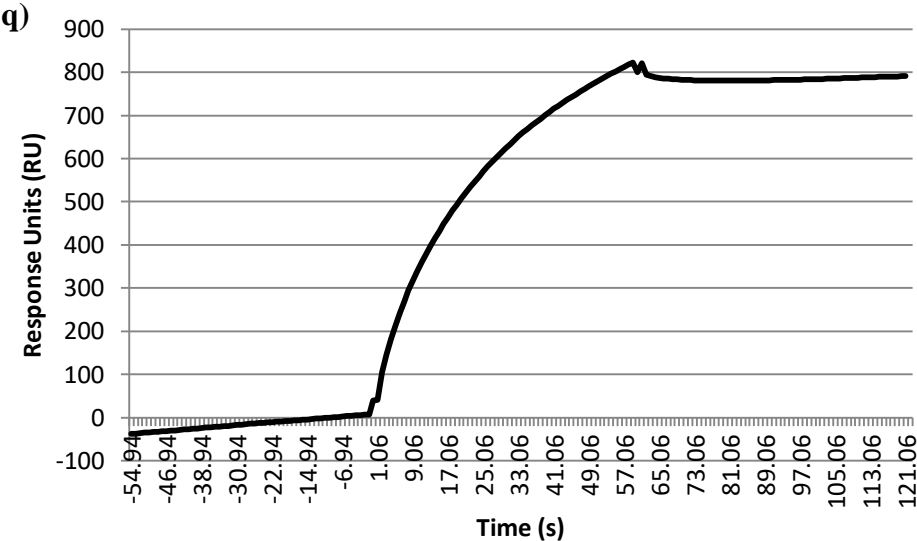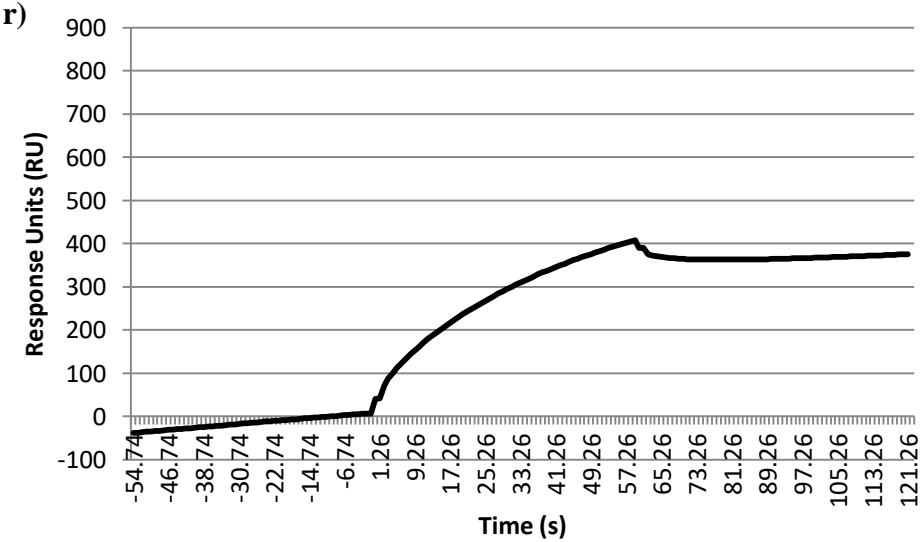

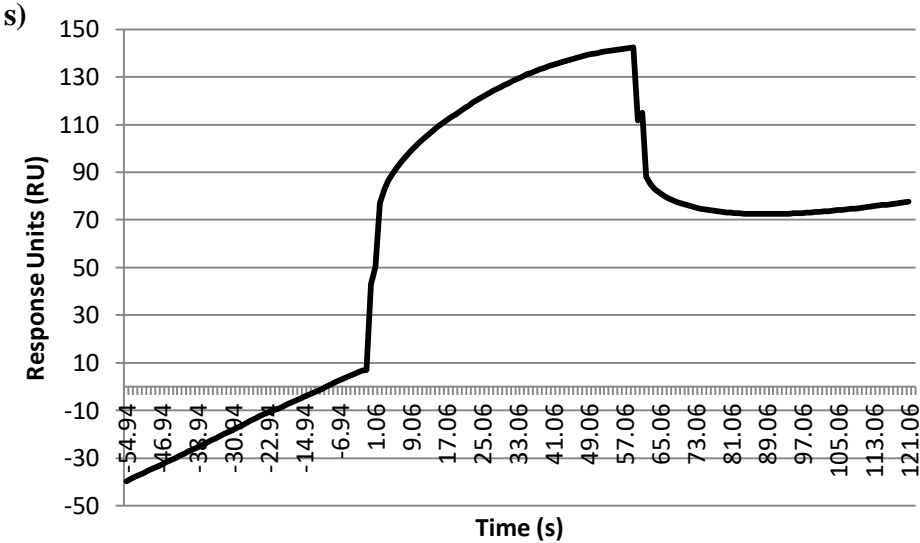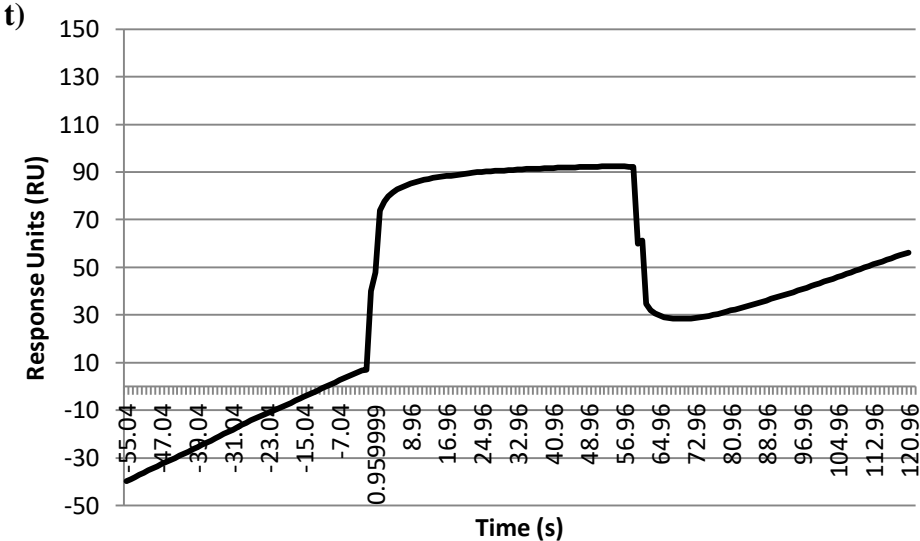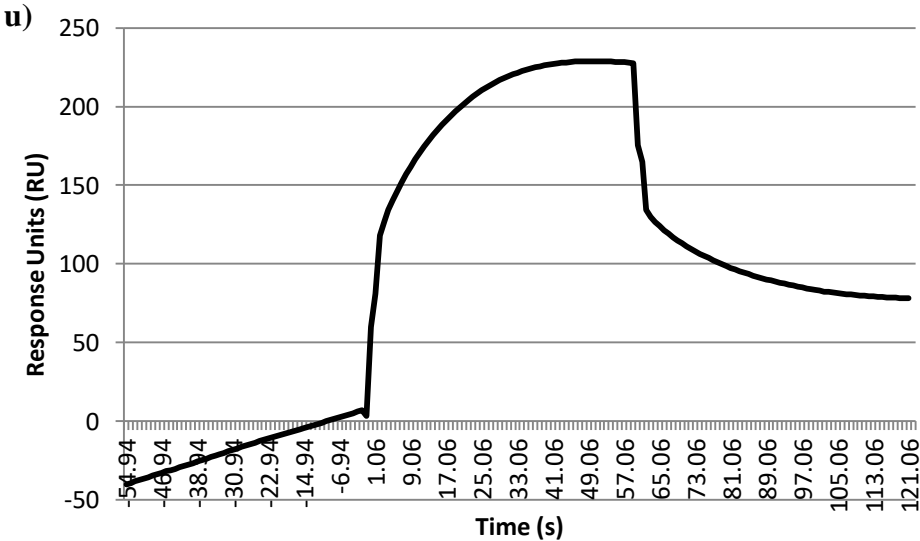

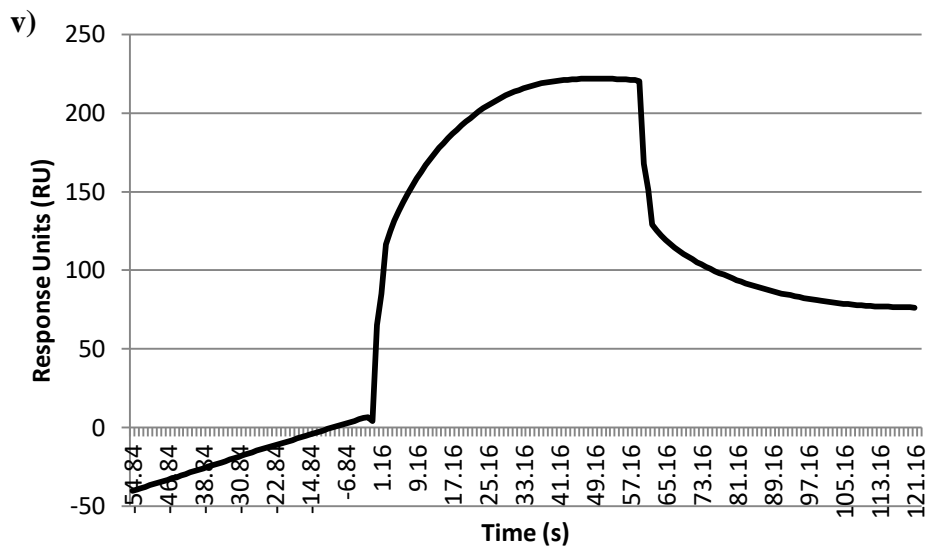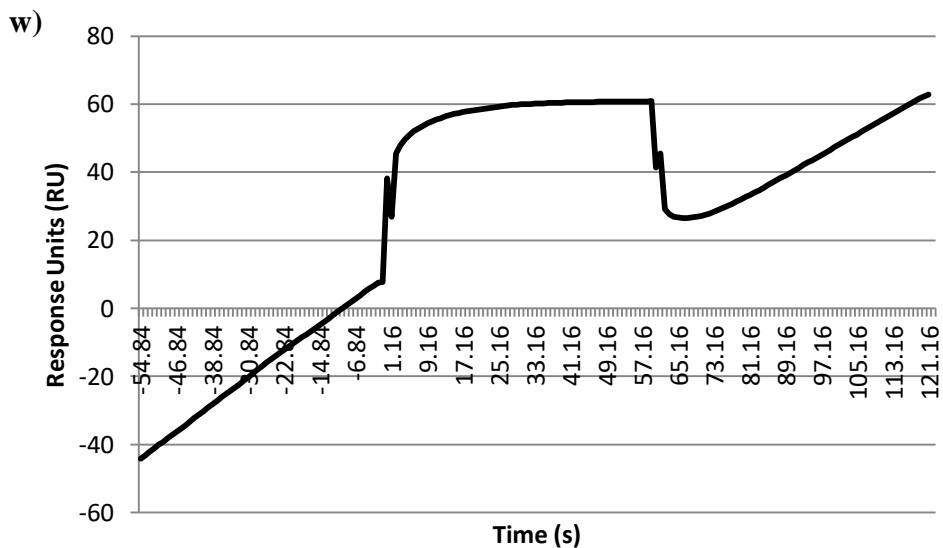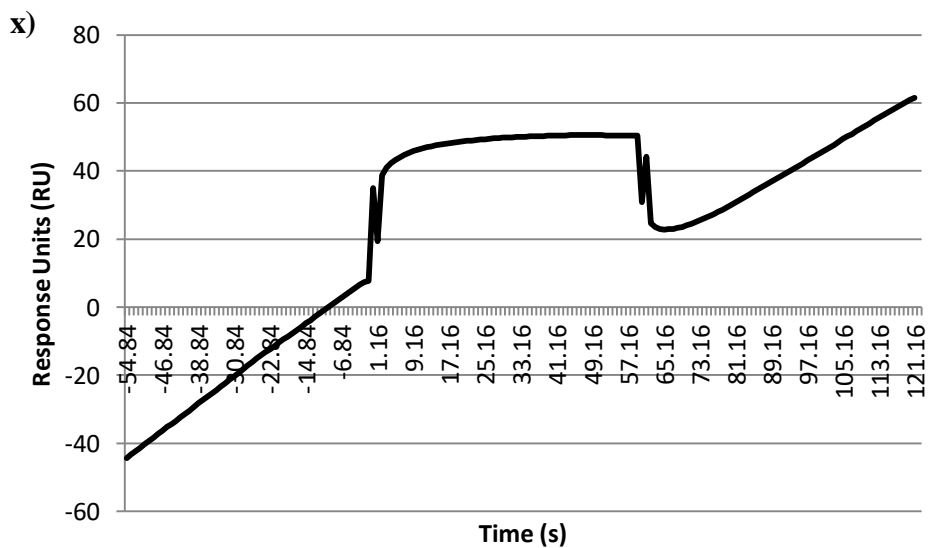

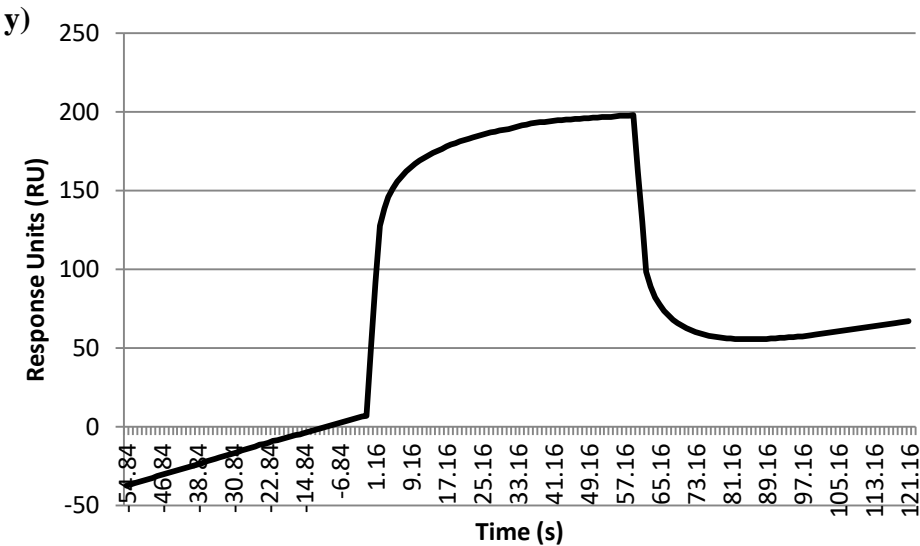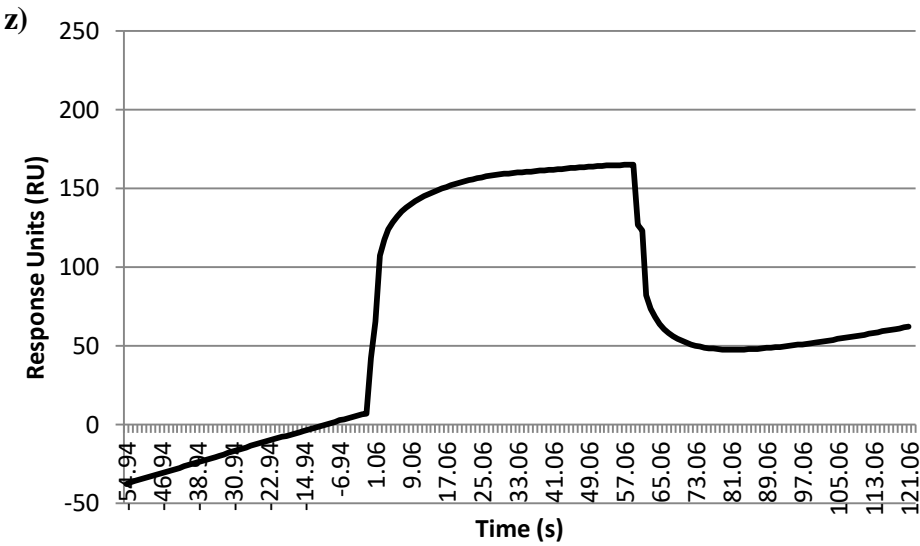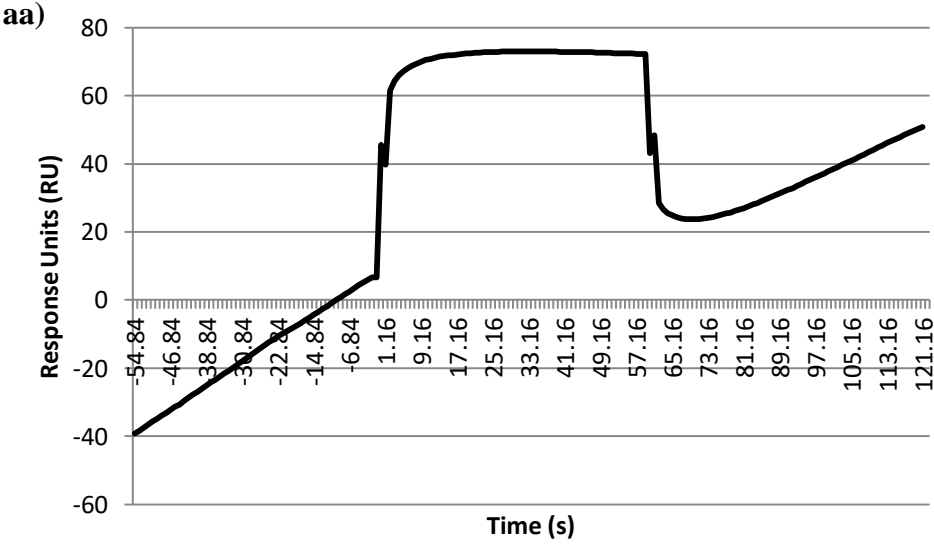

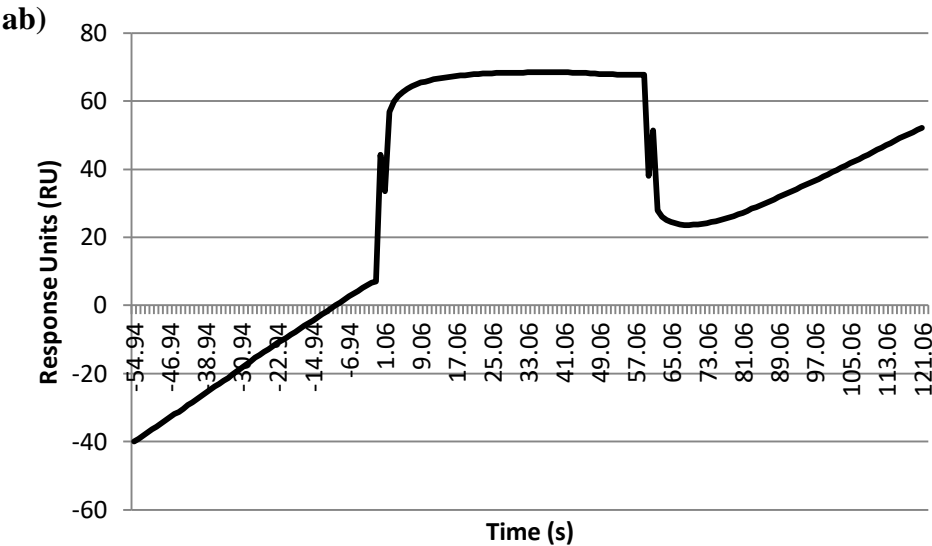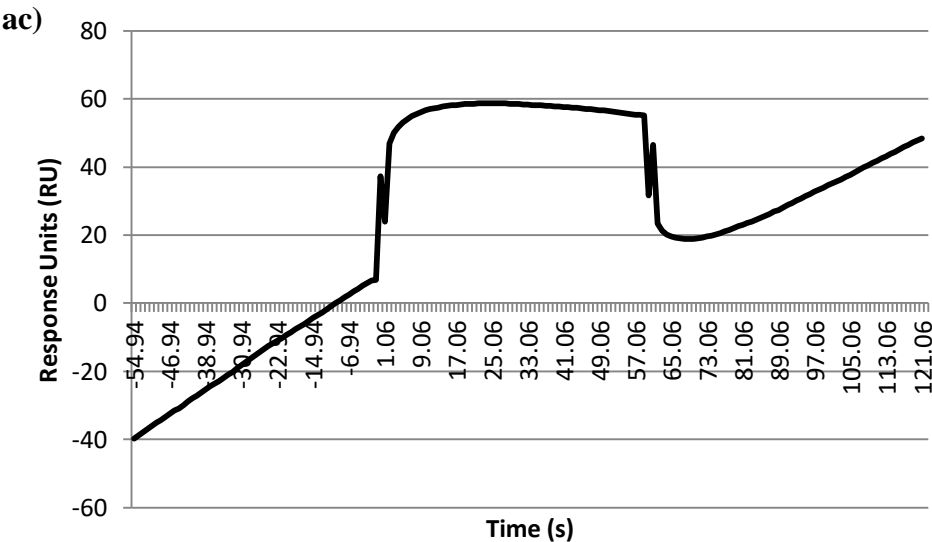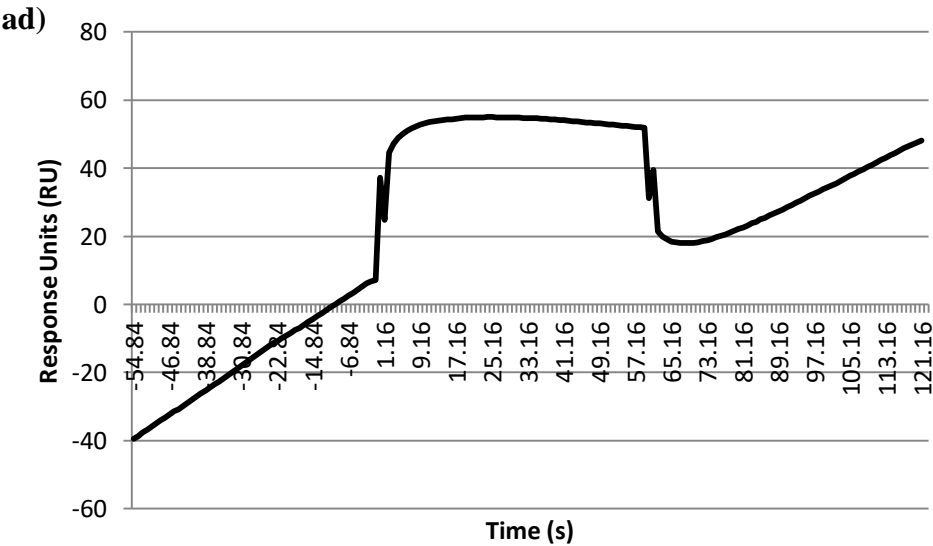

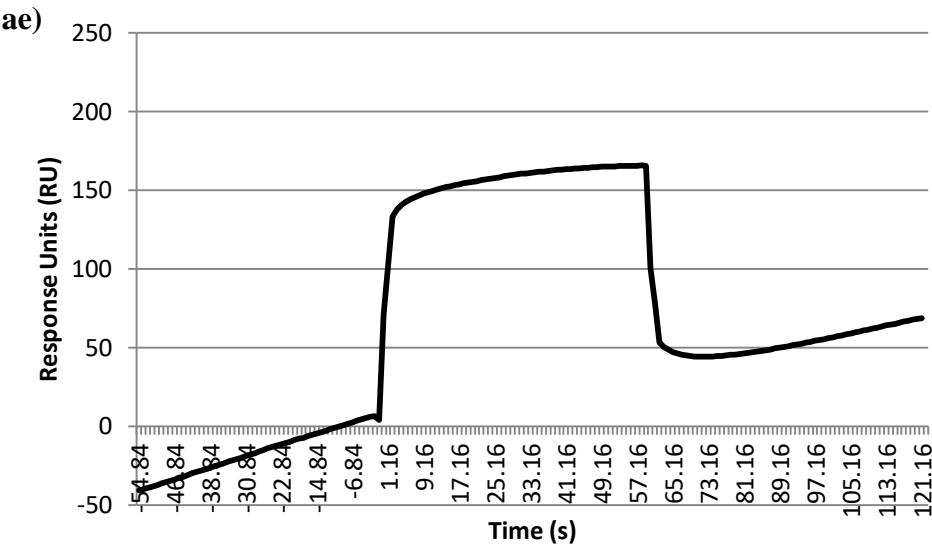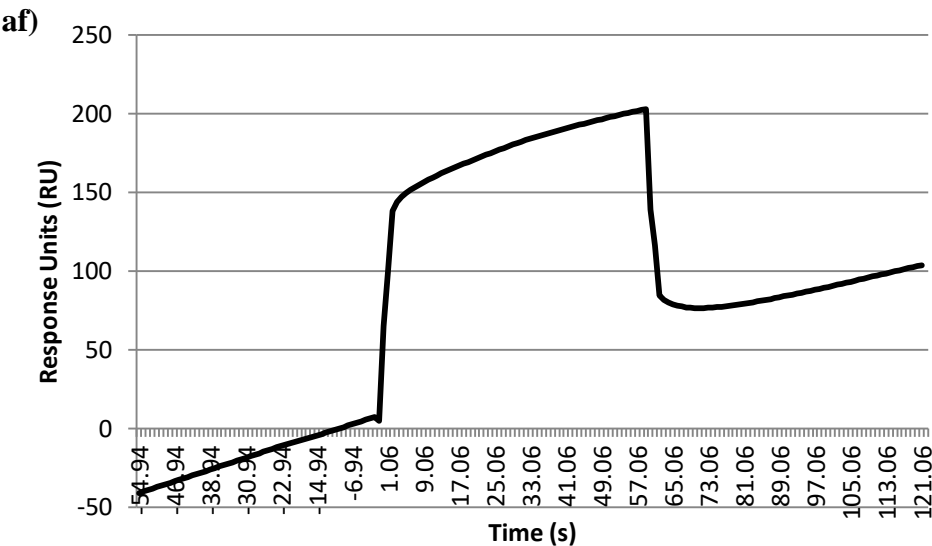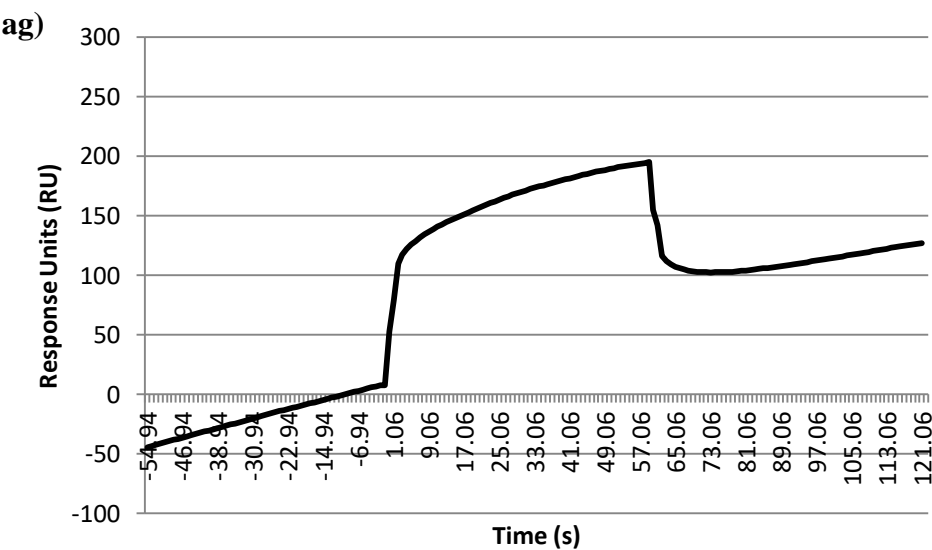

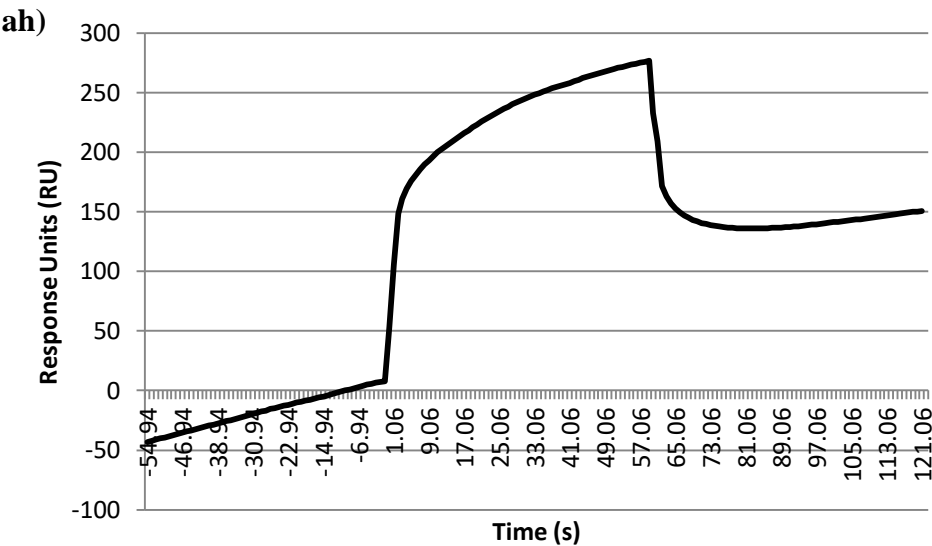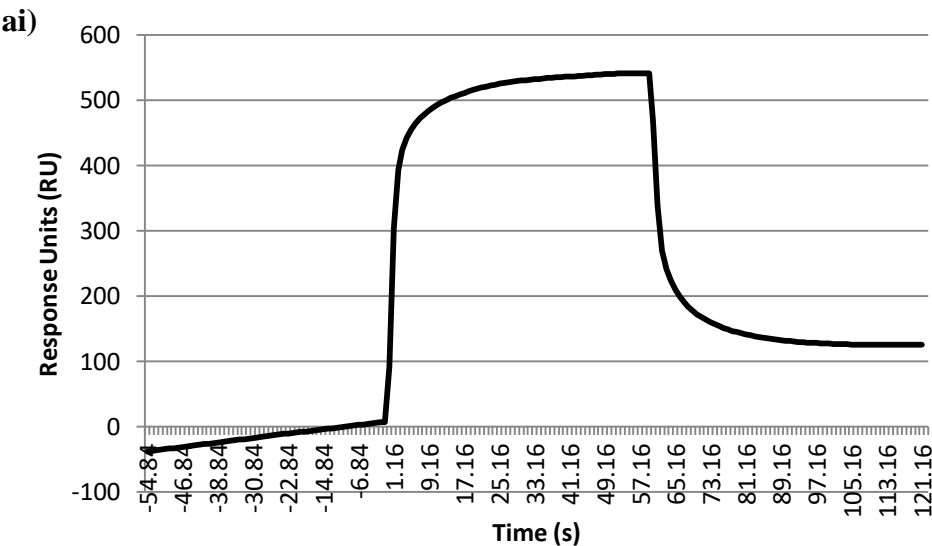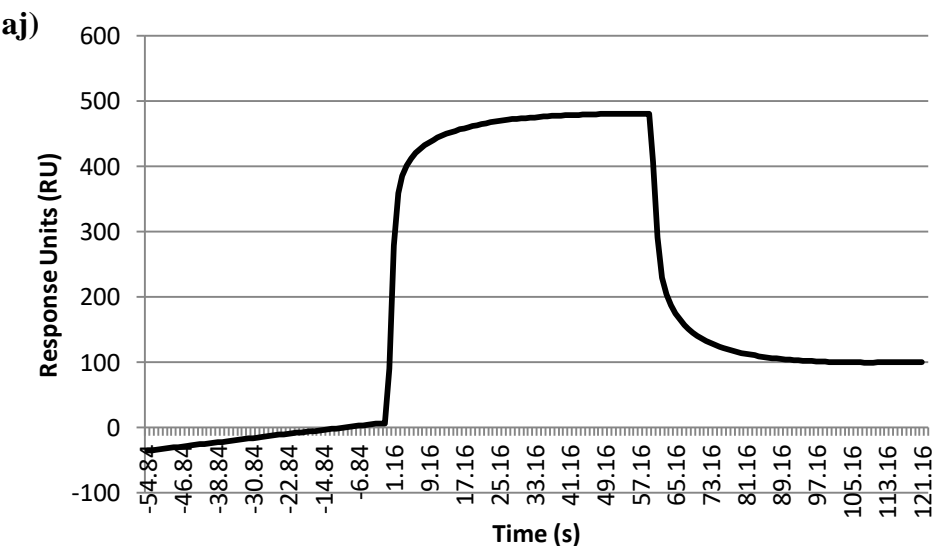

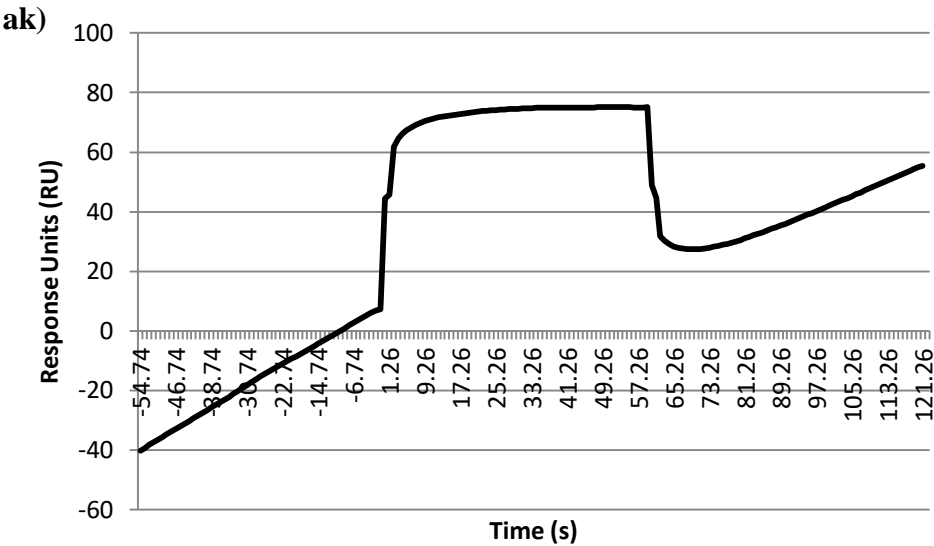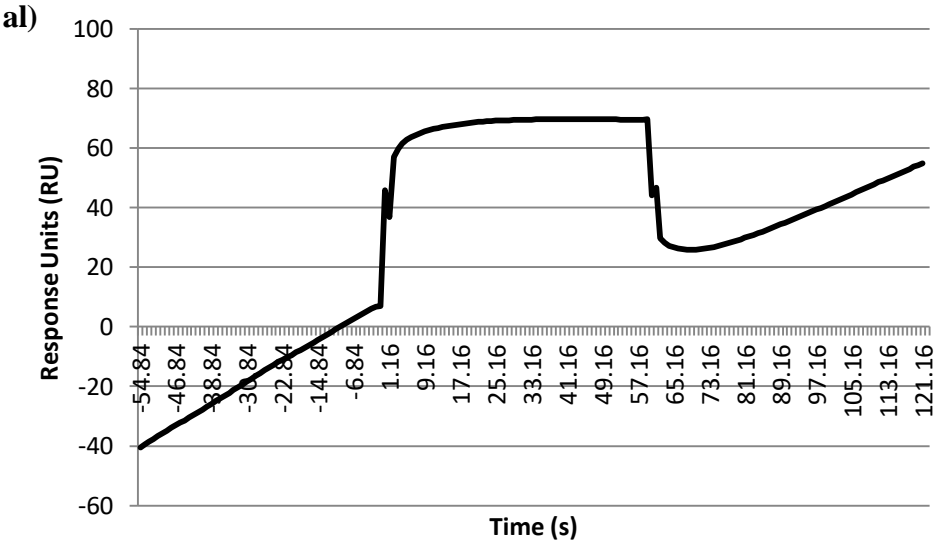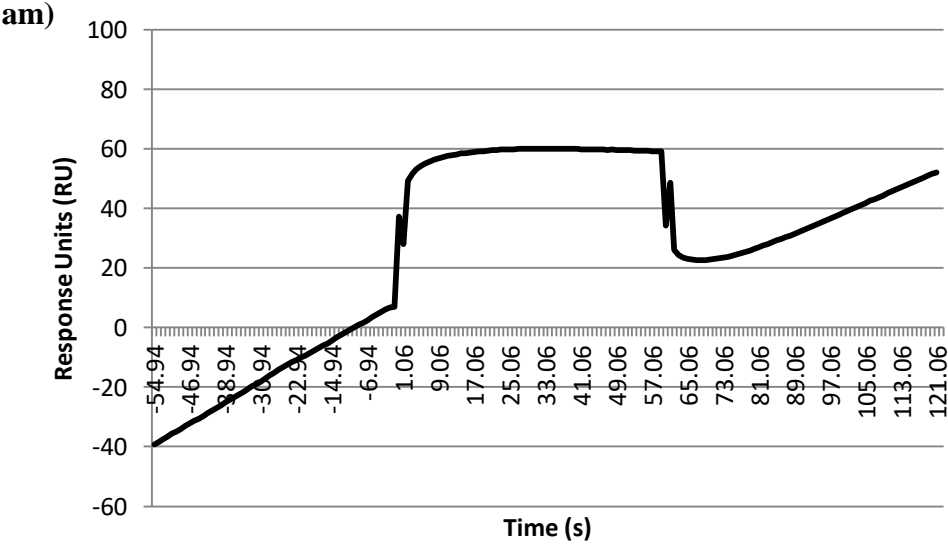

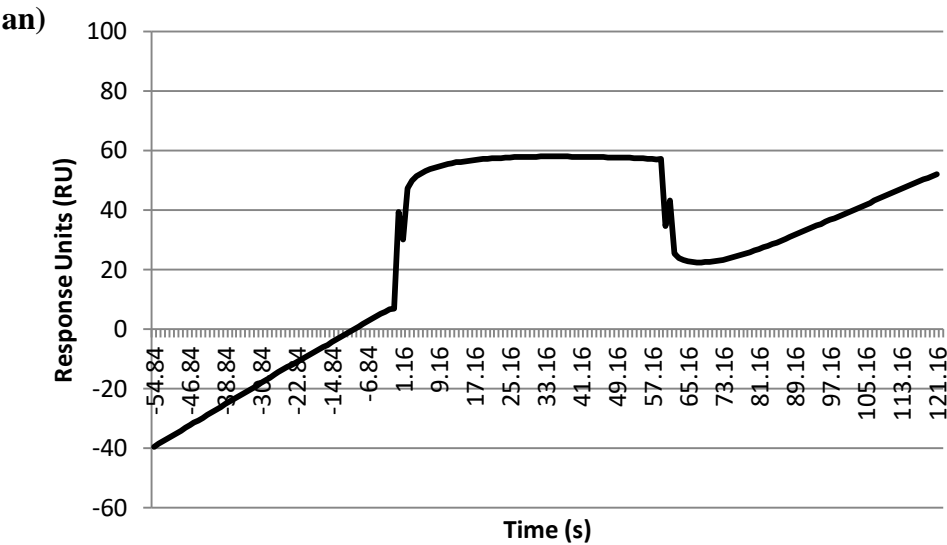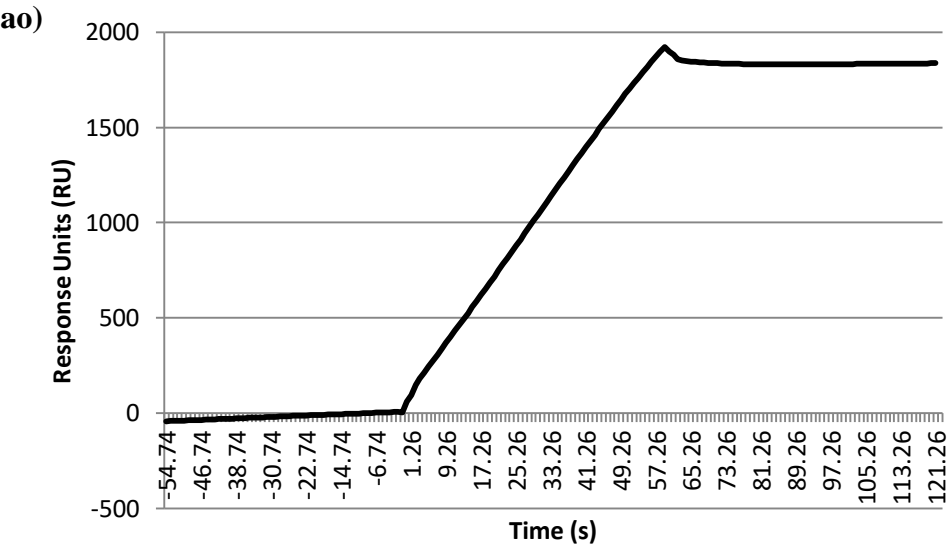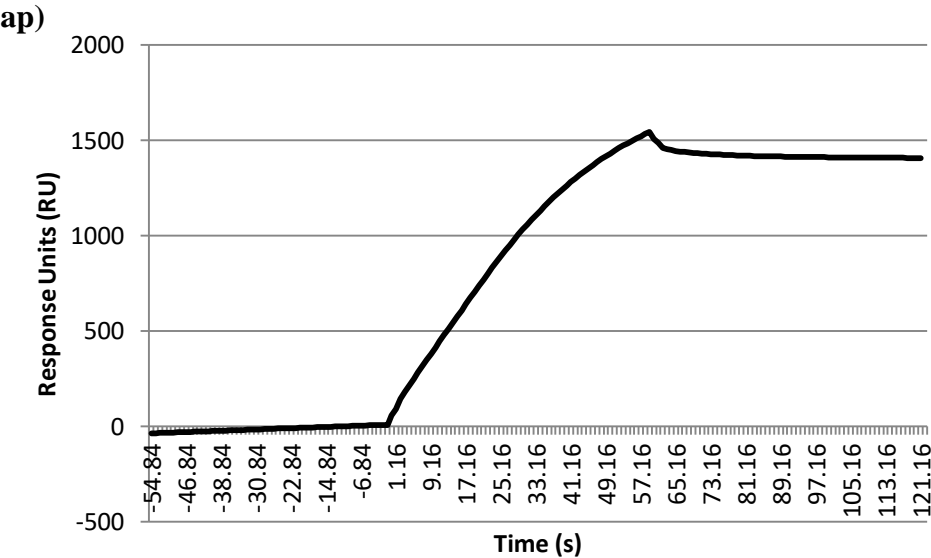

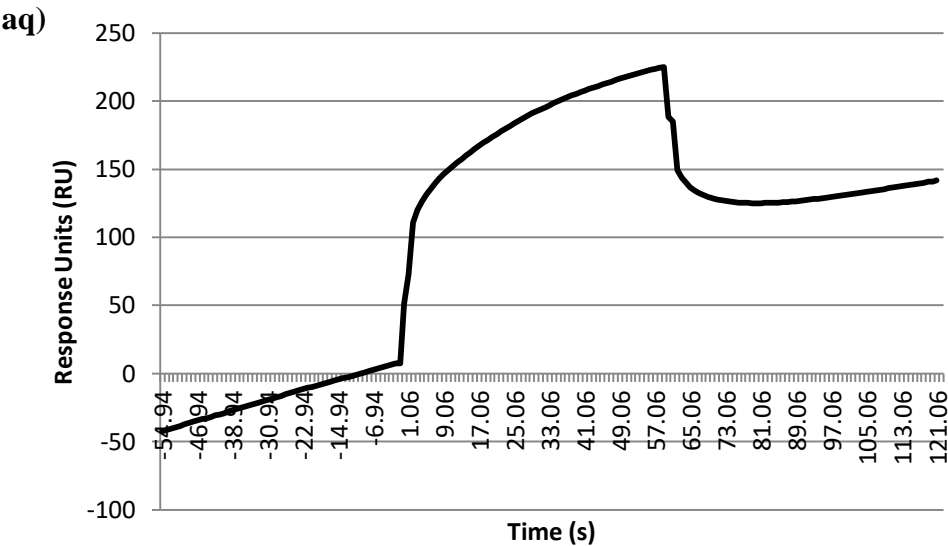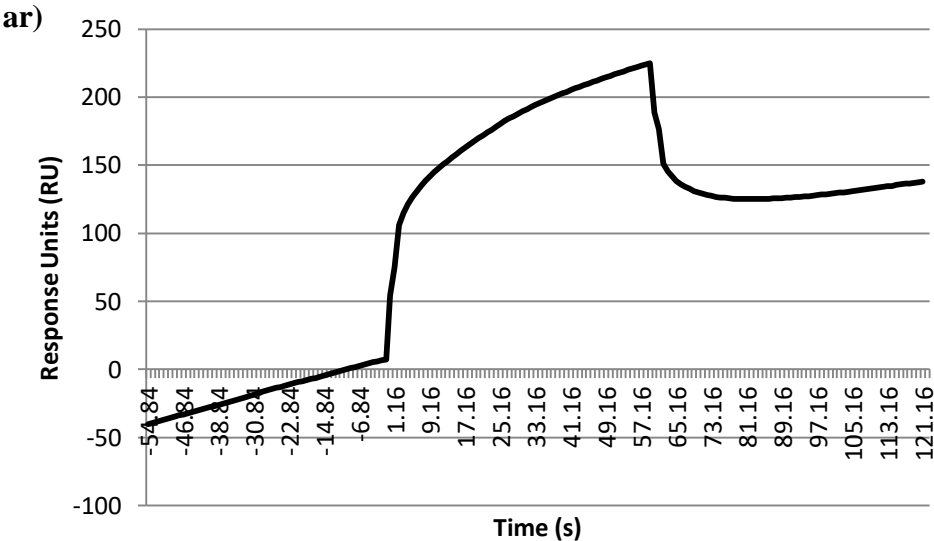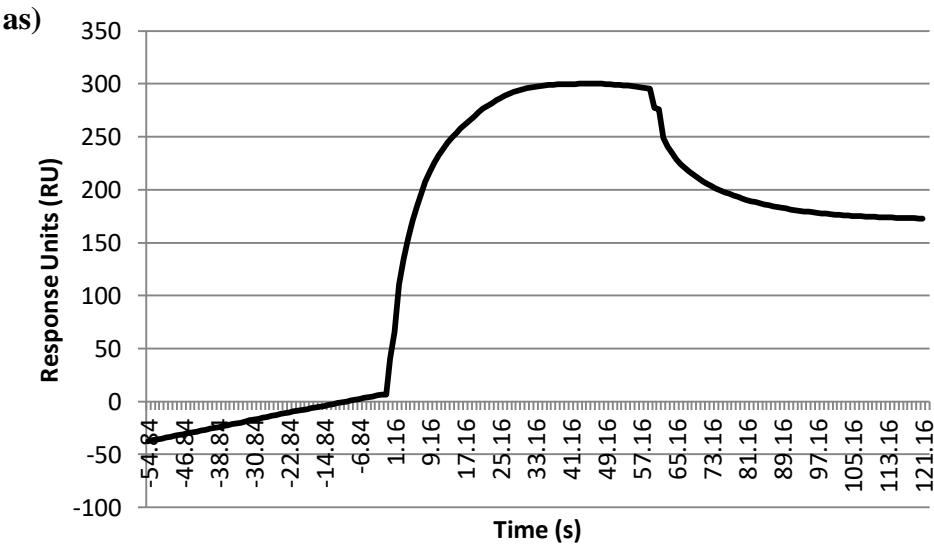

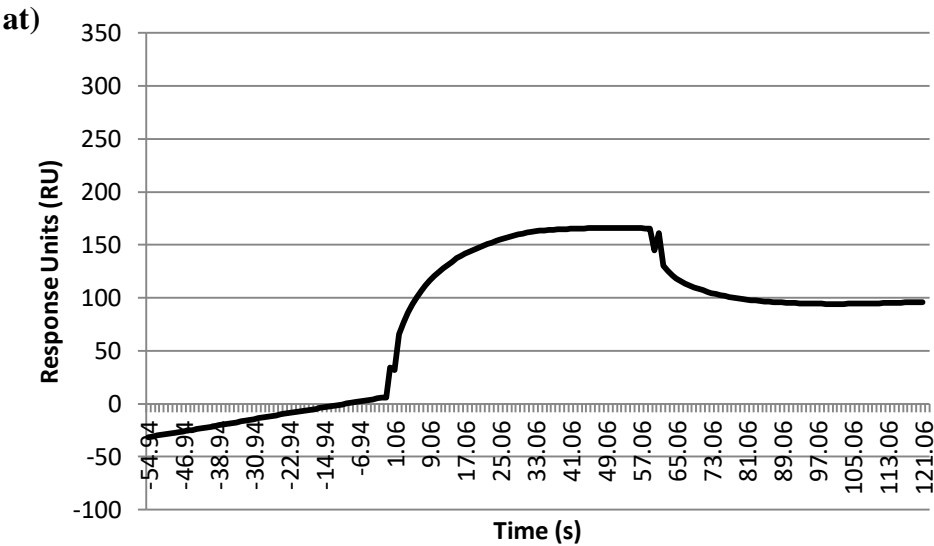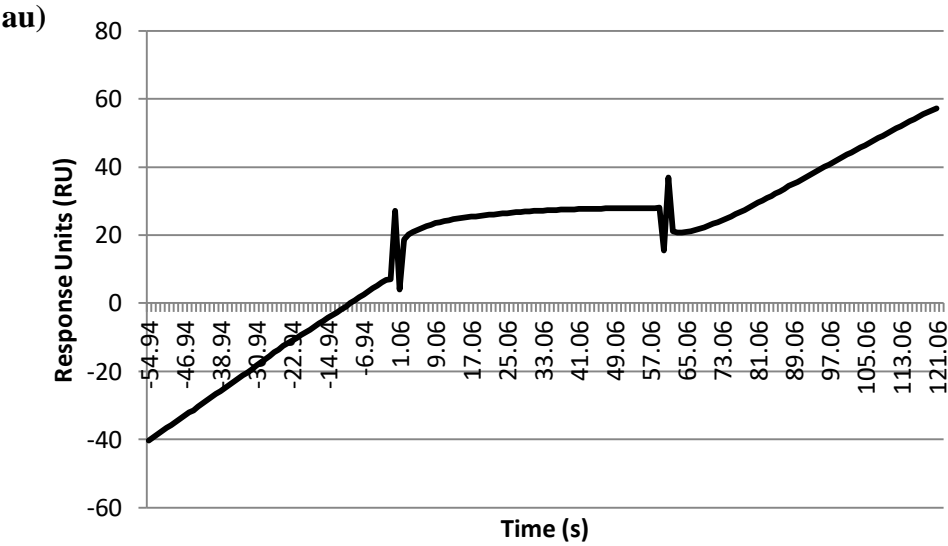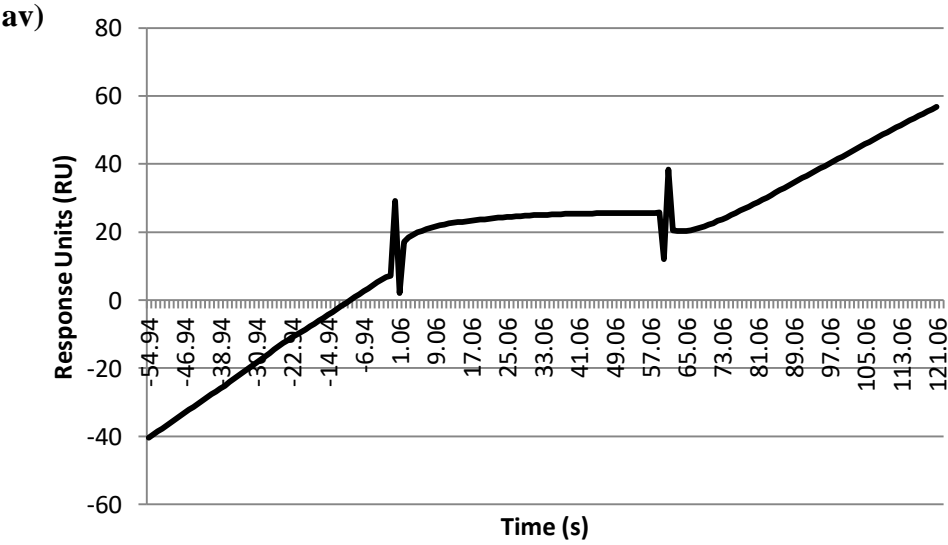

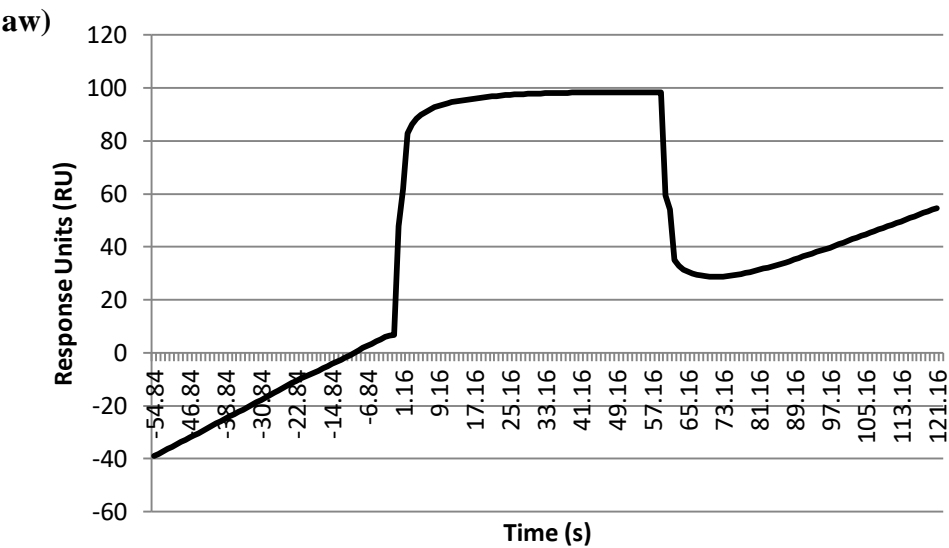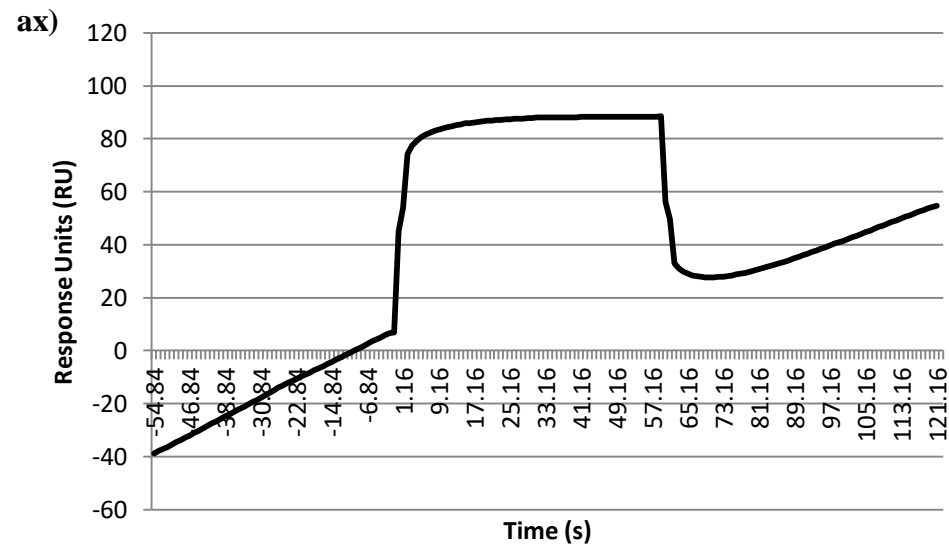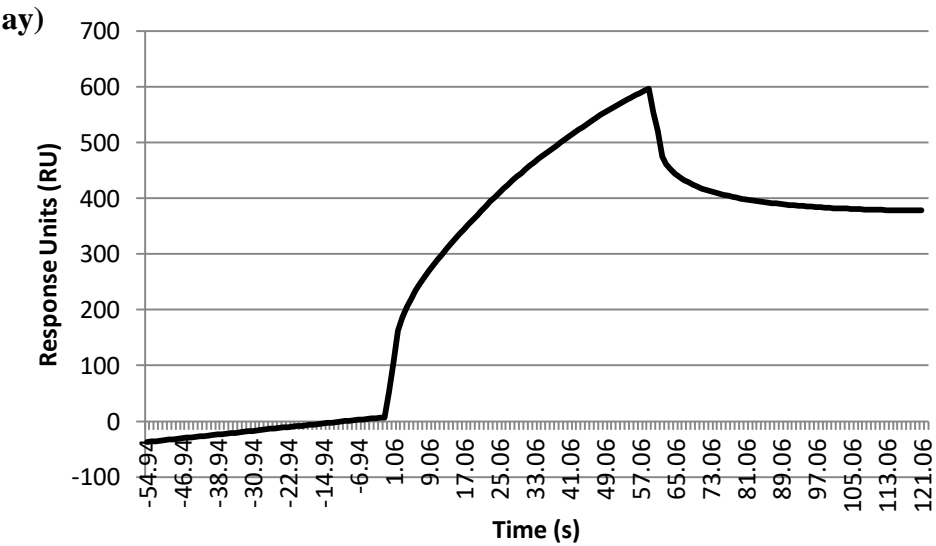

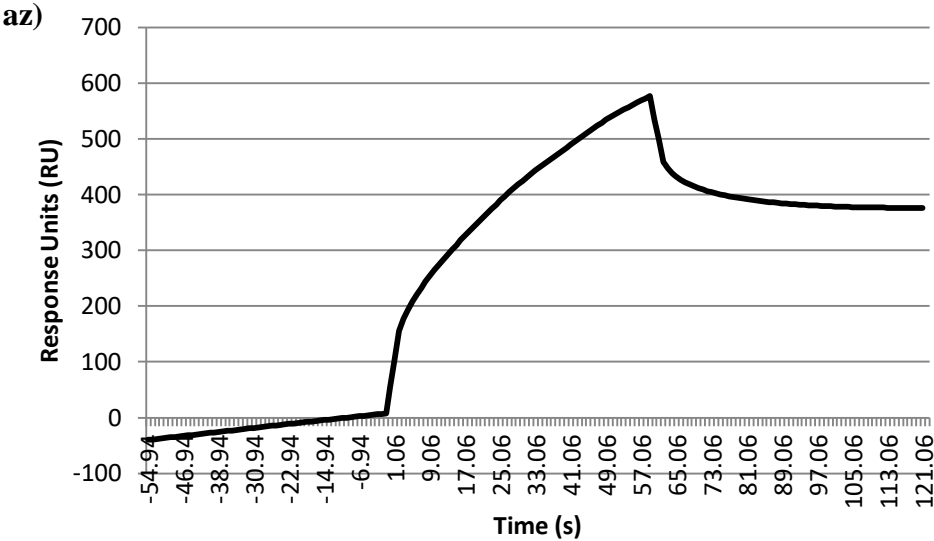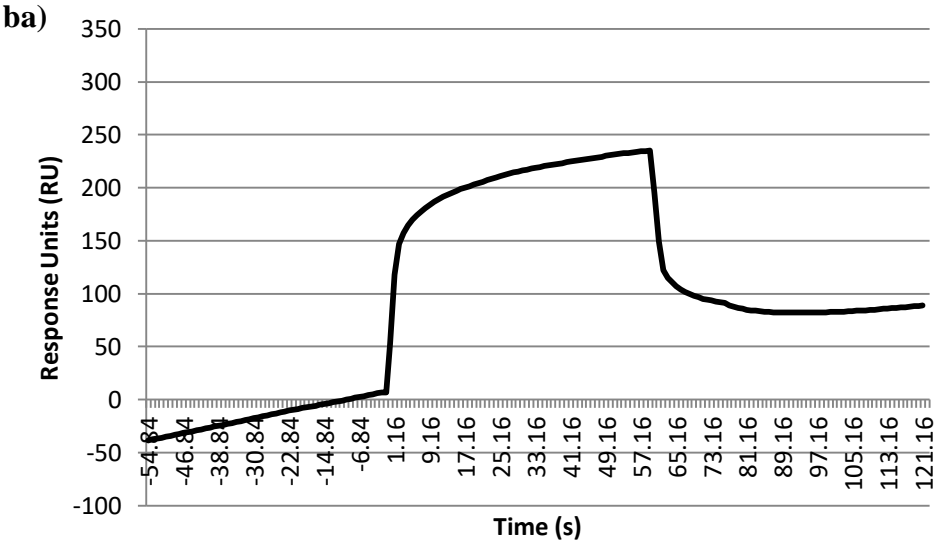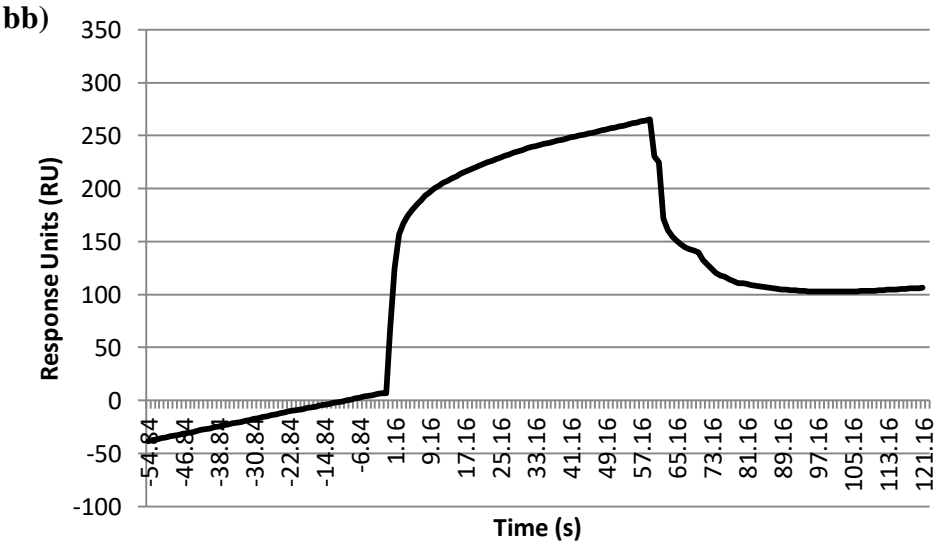

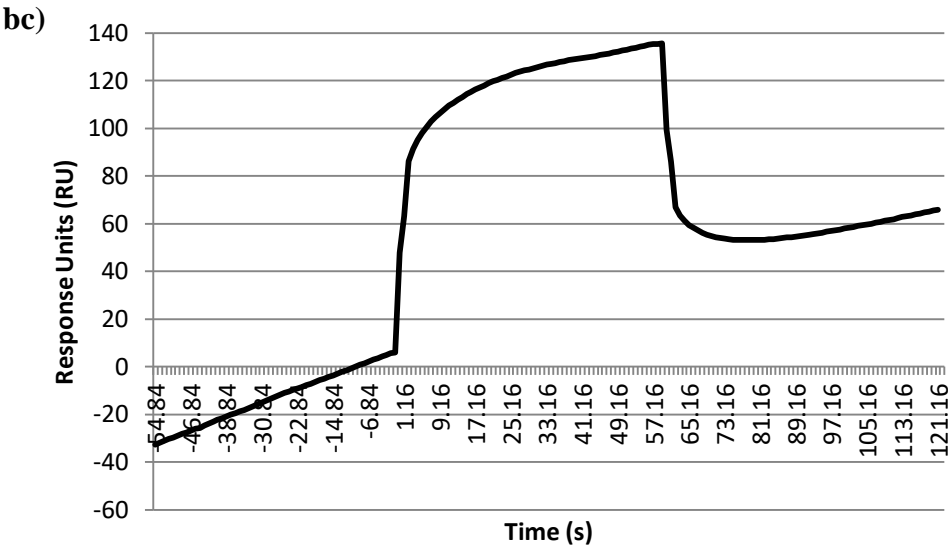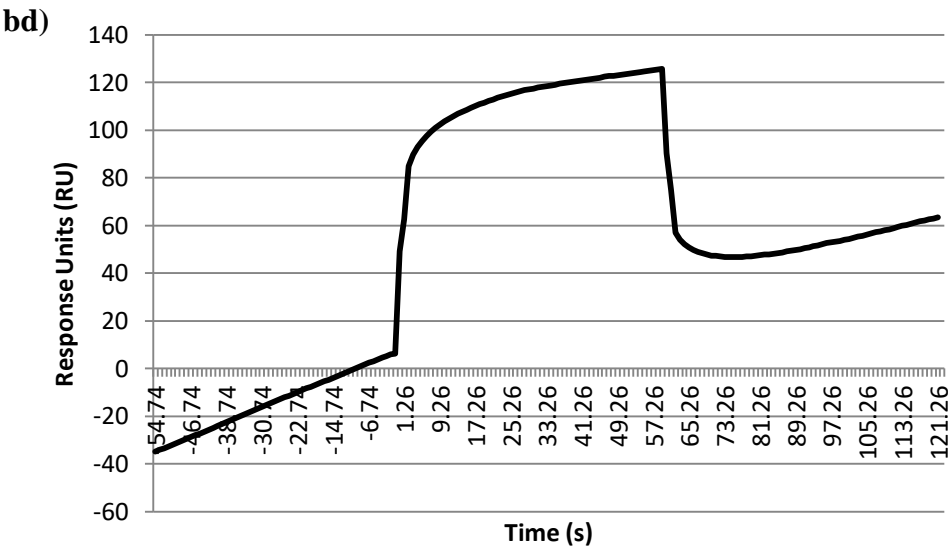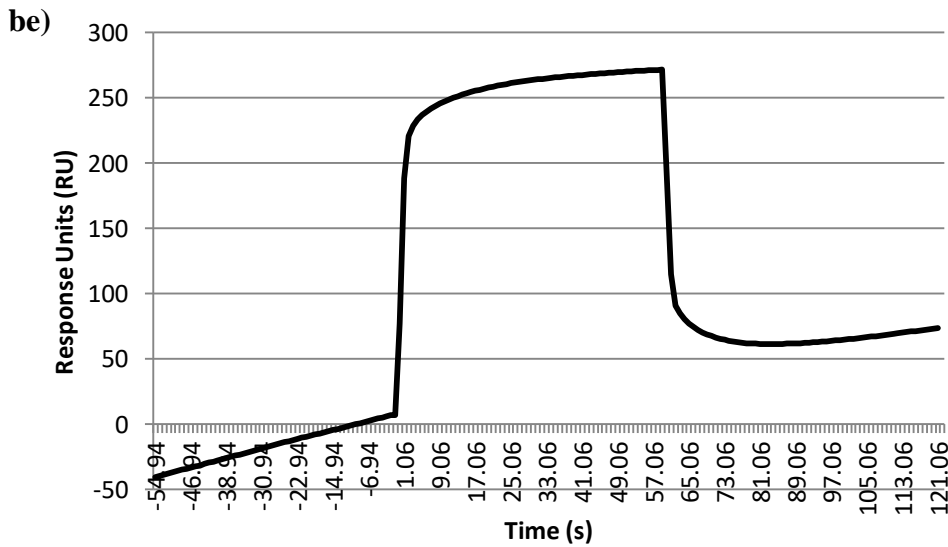

bf)

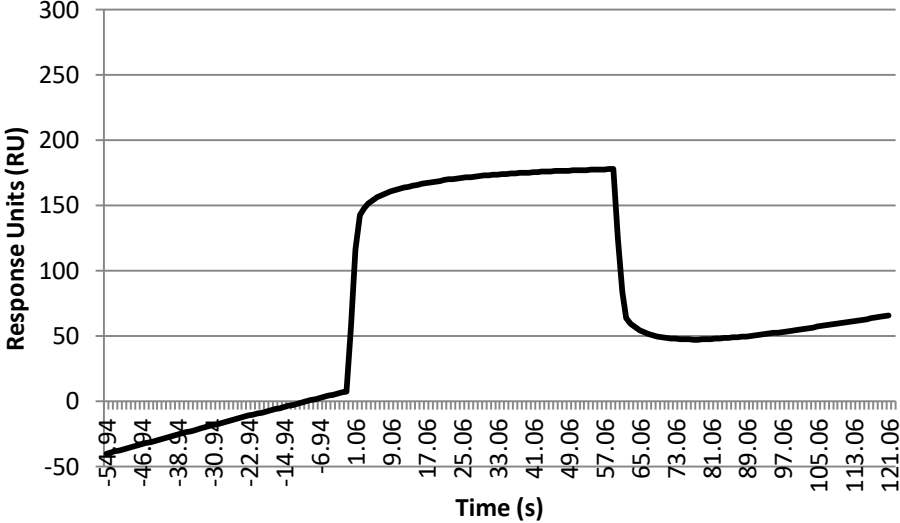

bg)

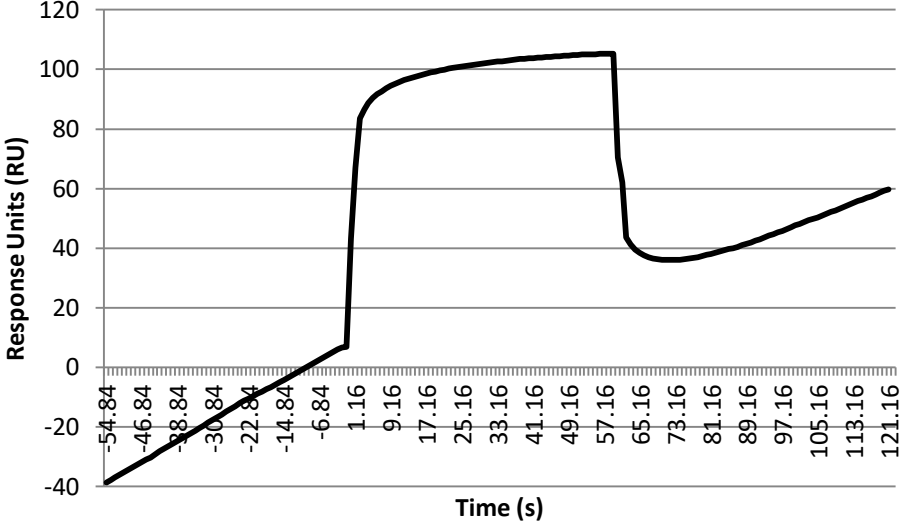

bh)

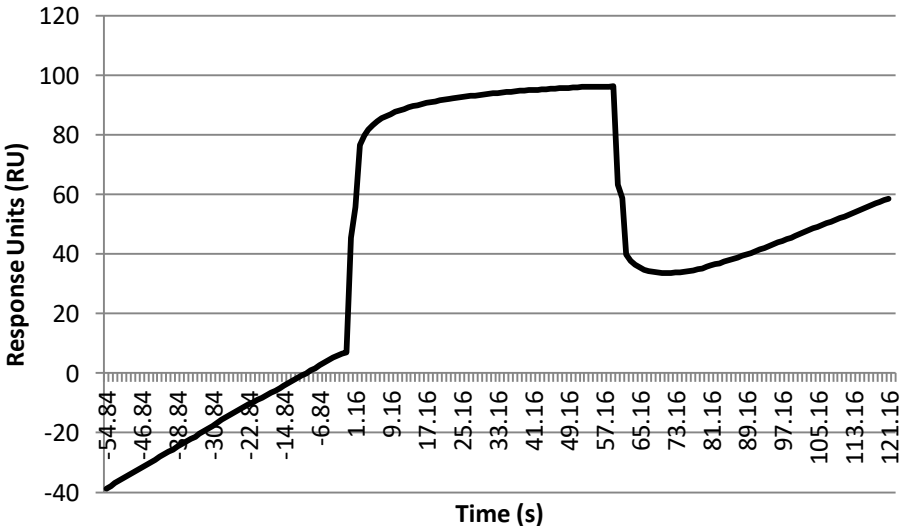

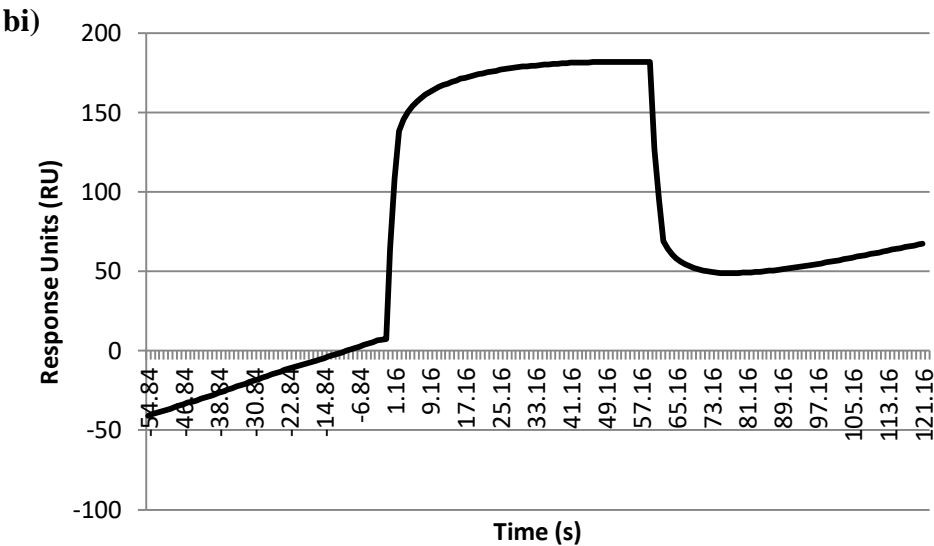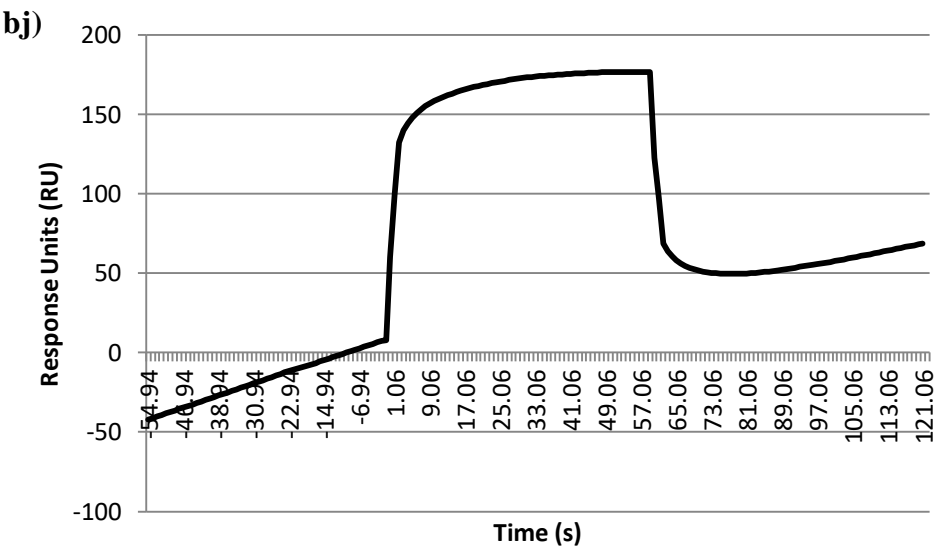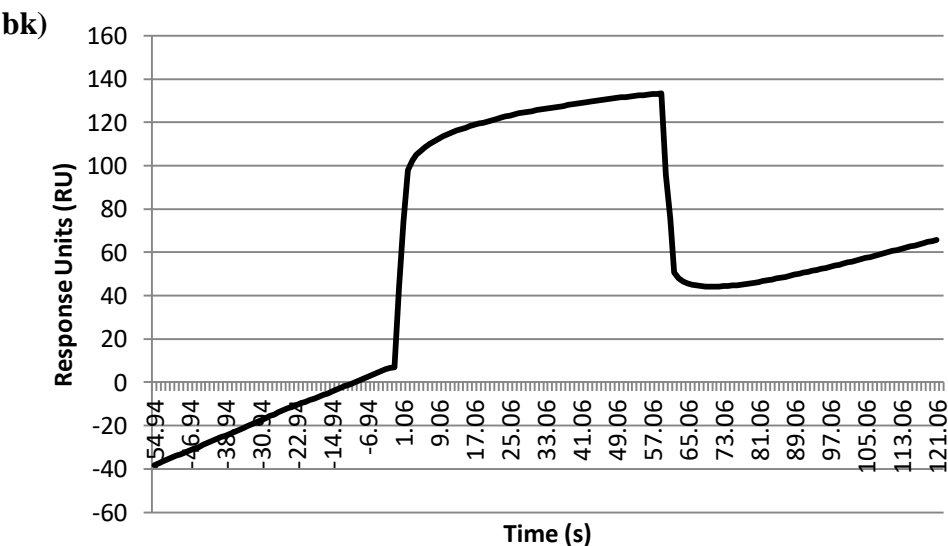

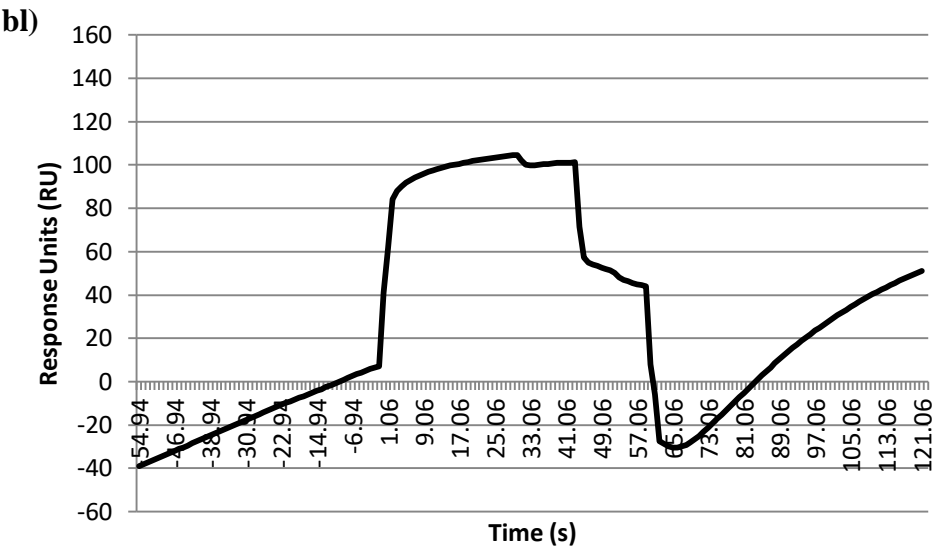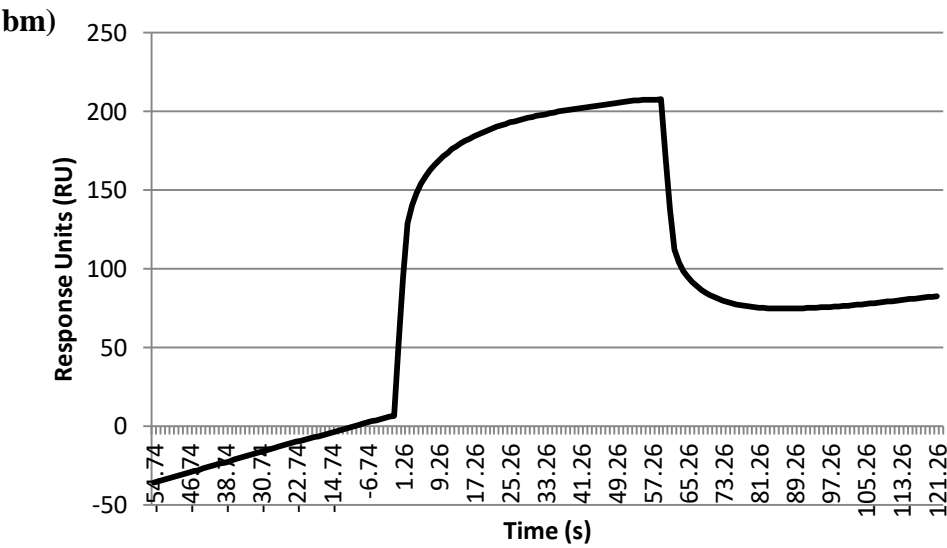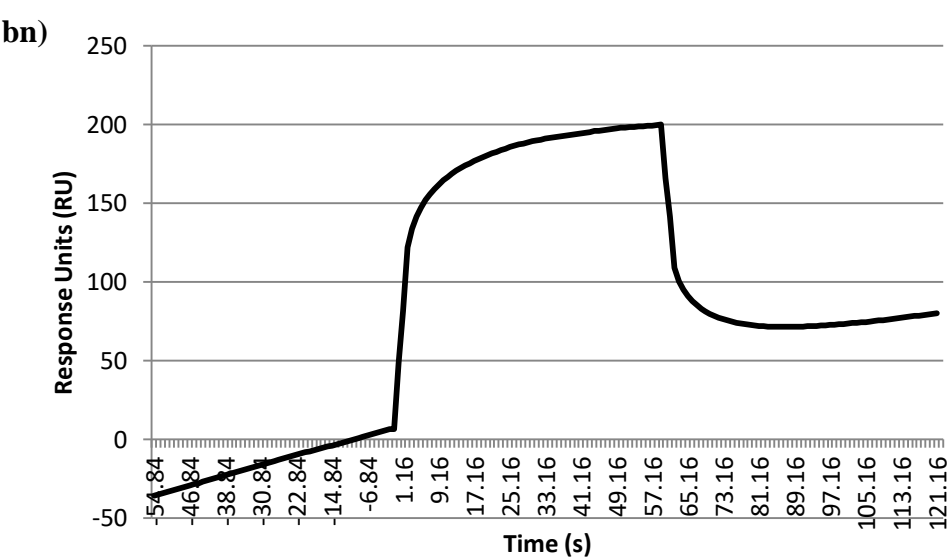

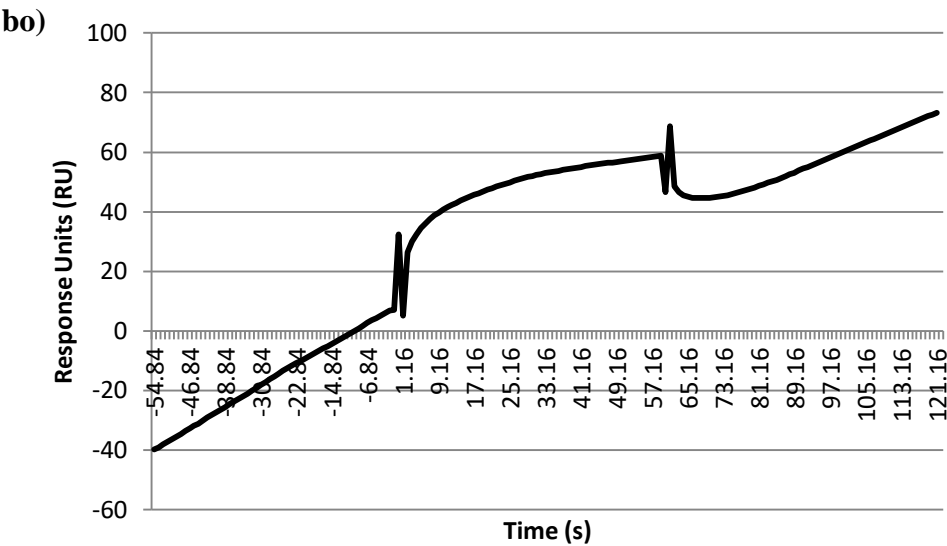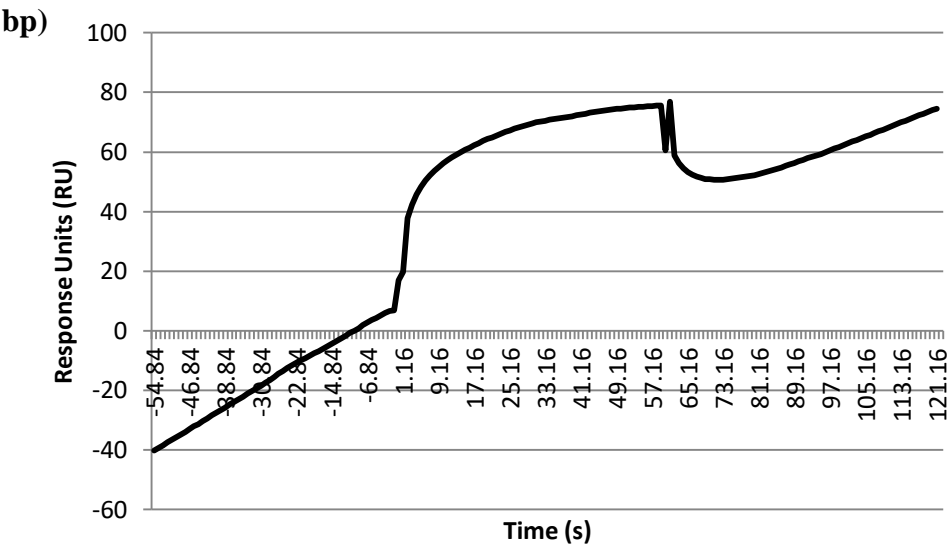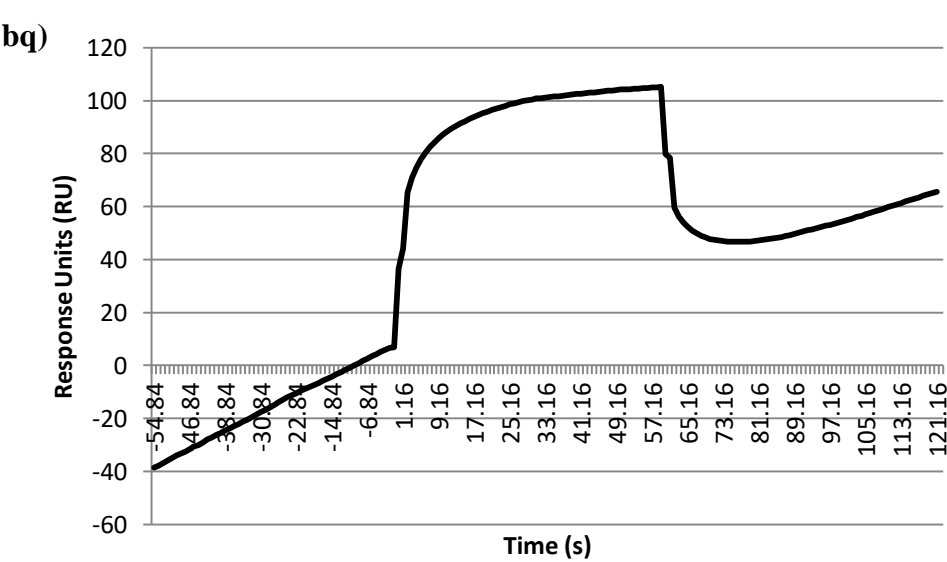

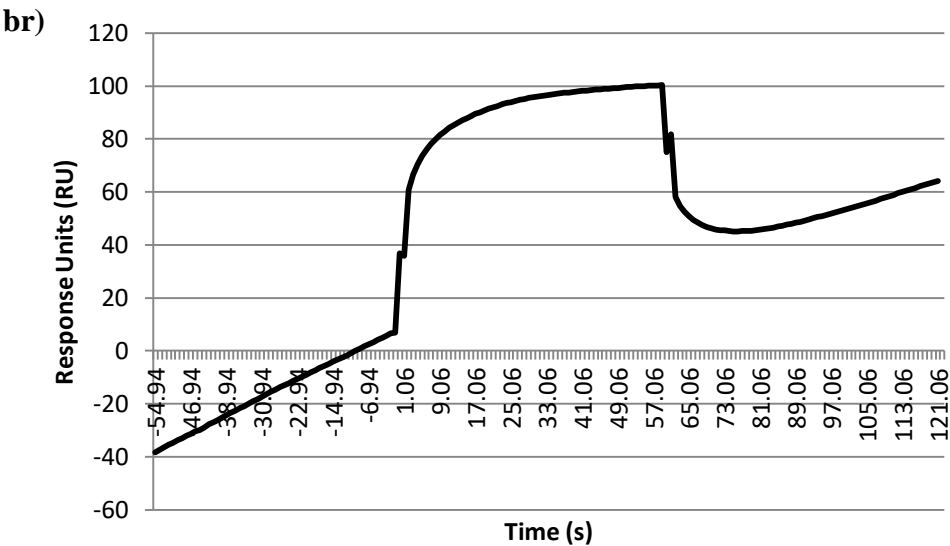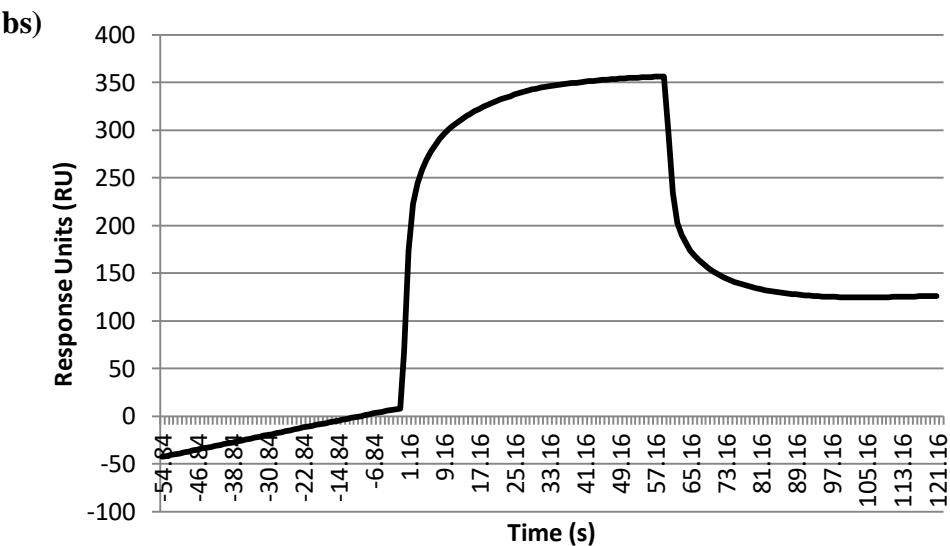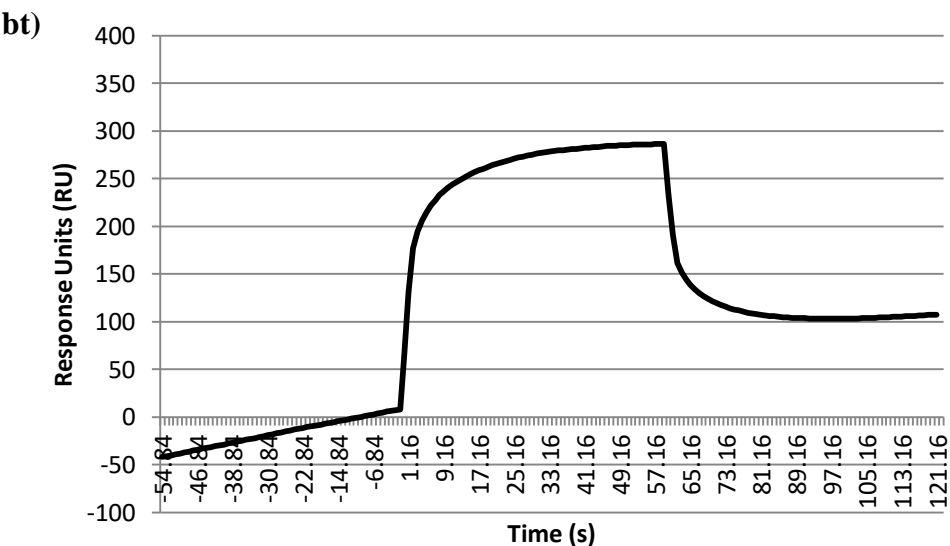

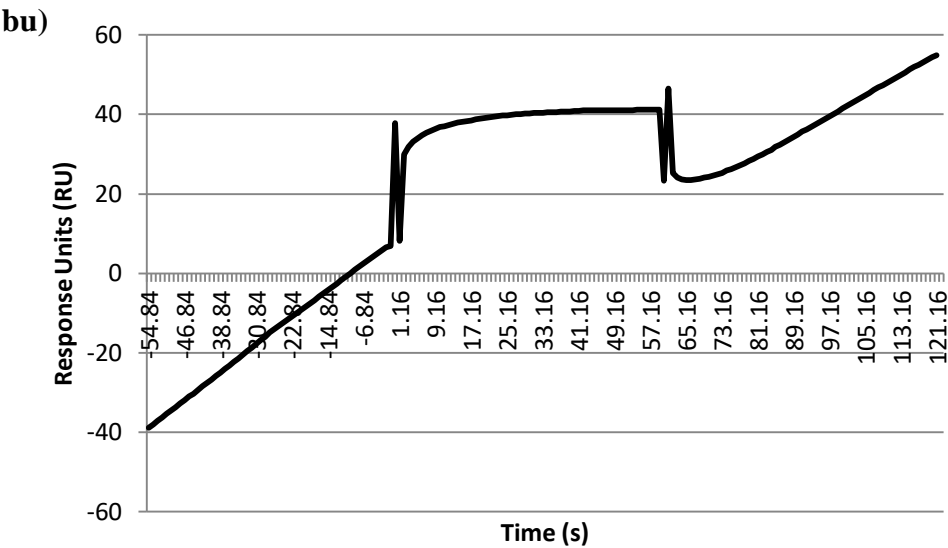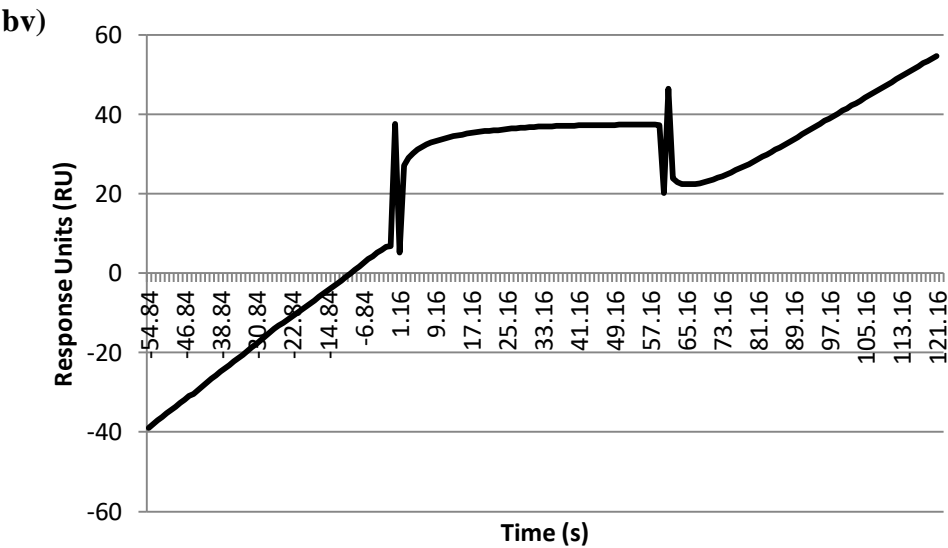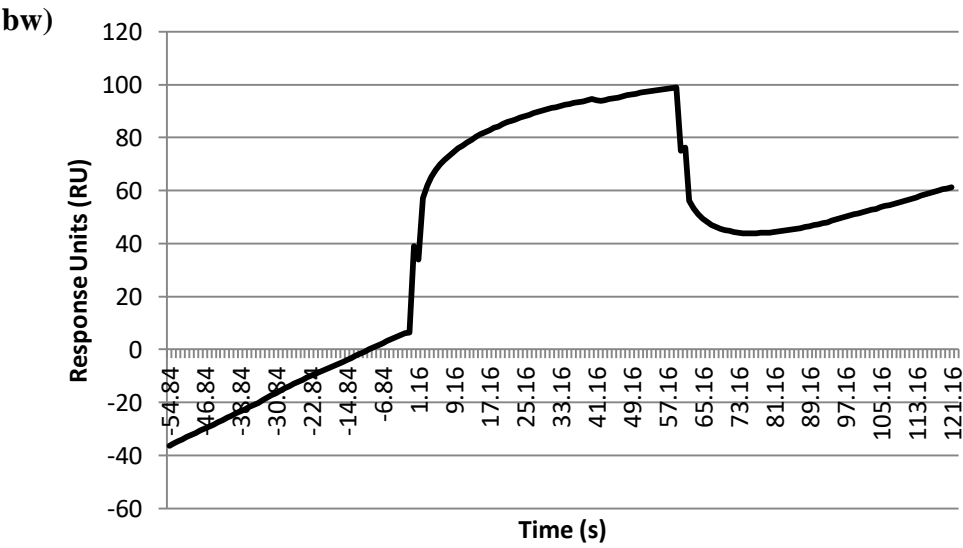

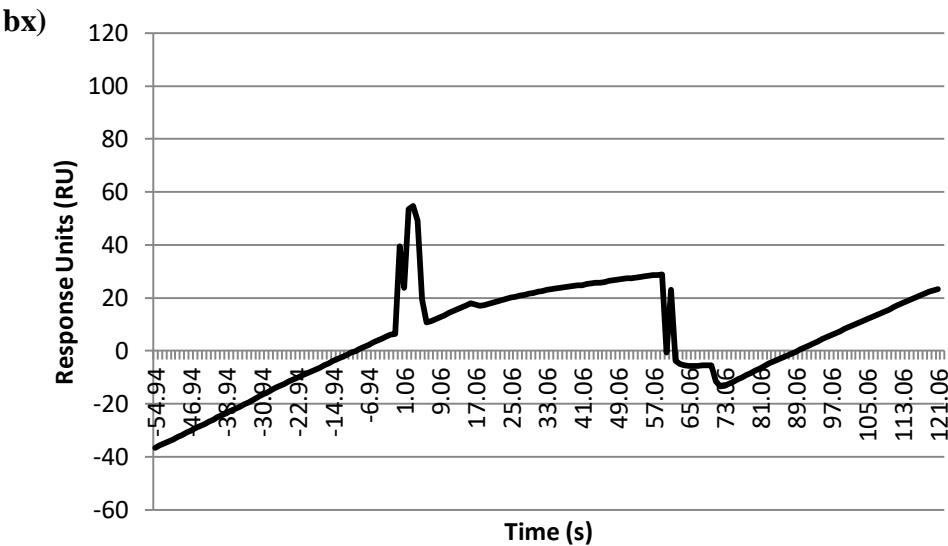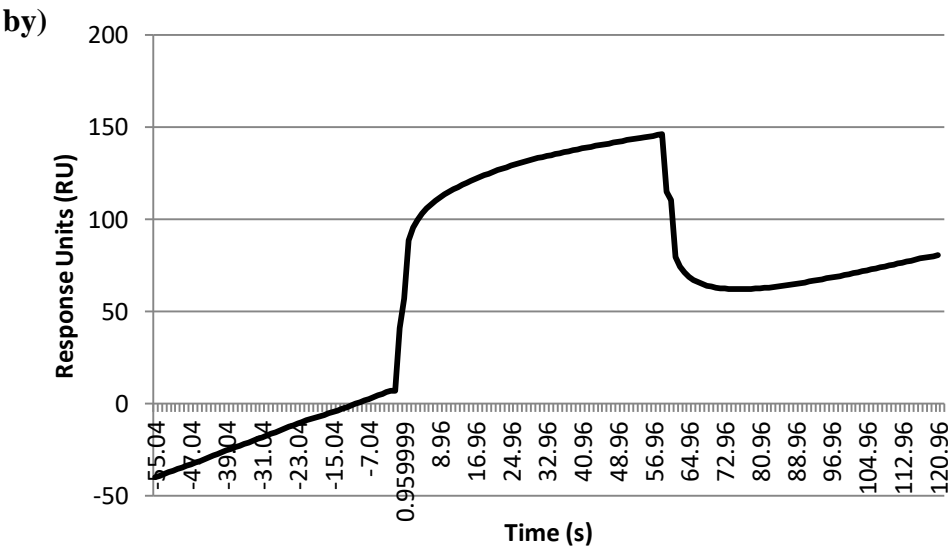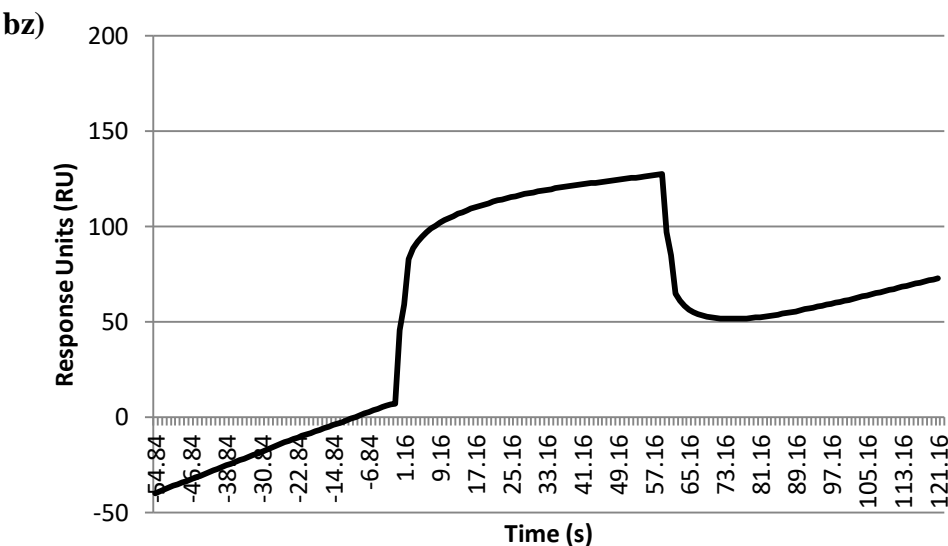

ca)

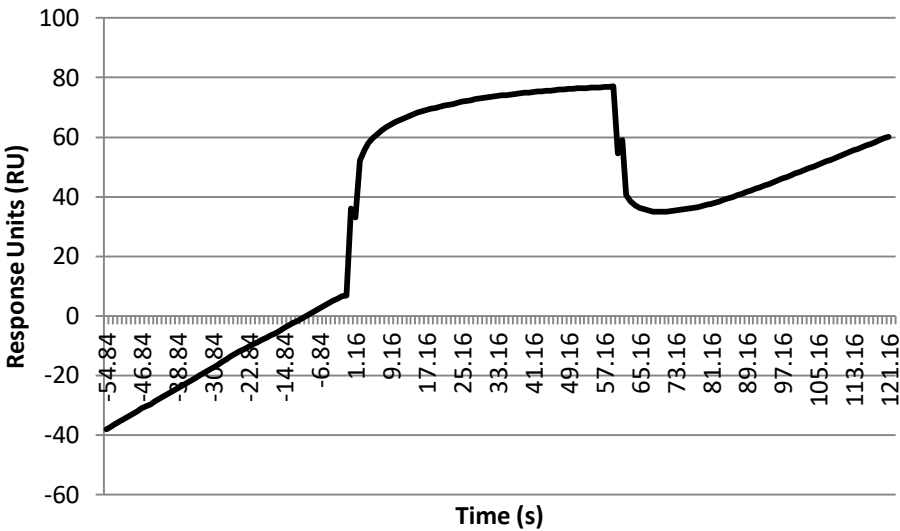

cb)

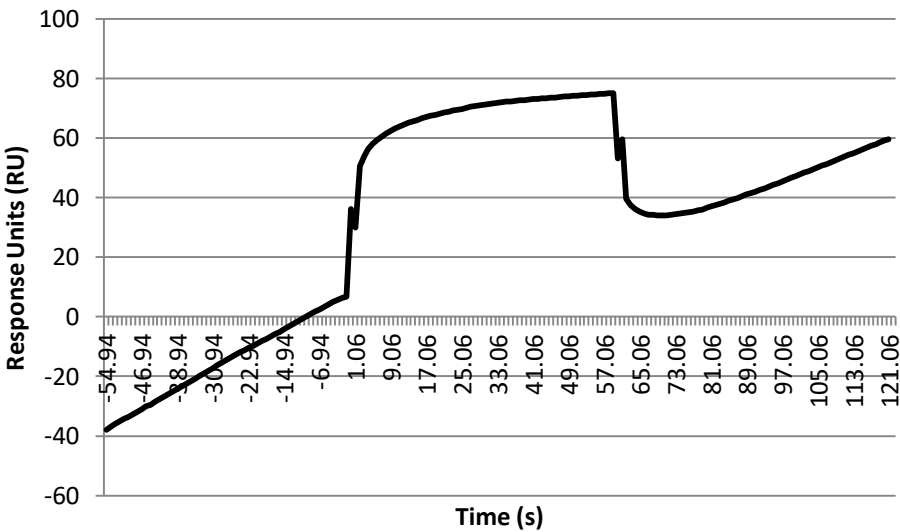

Supplement: FIG S3 [file mBio.03046-19-sf003.pdf]

a)

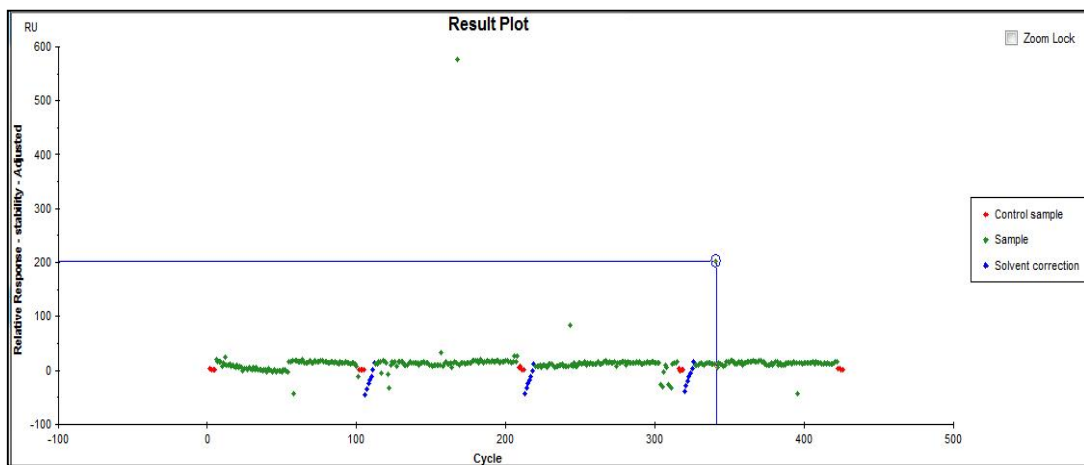

b)

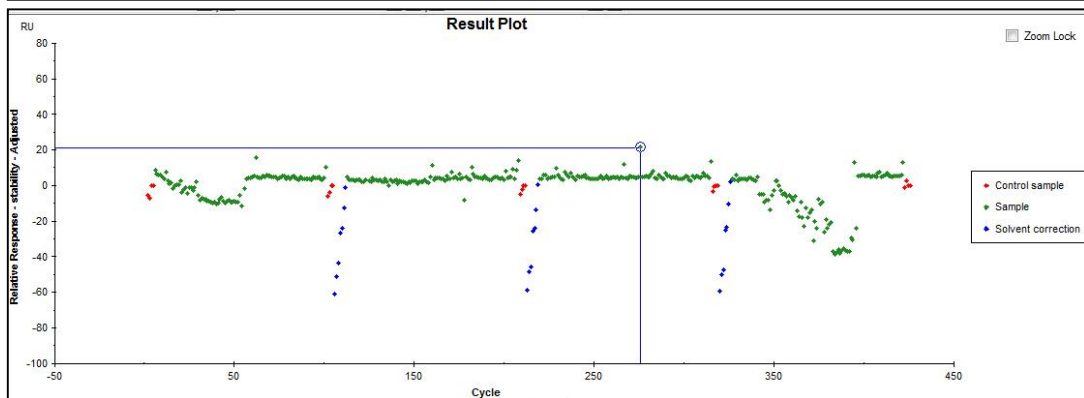

c)

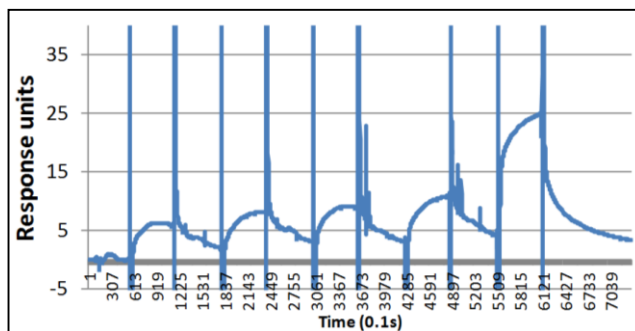

d)

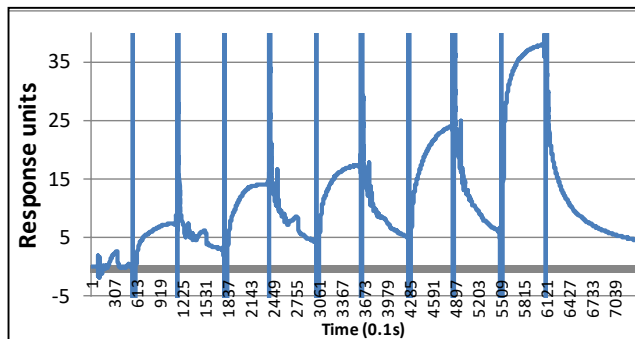

Supplement: FIG S5 [file mBio.03046-19-sf005.pdf]

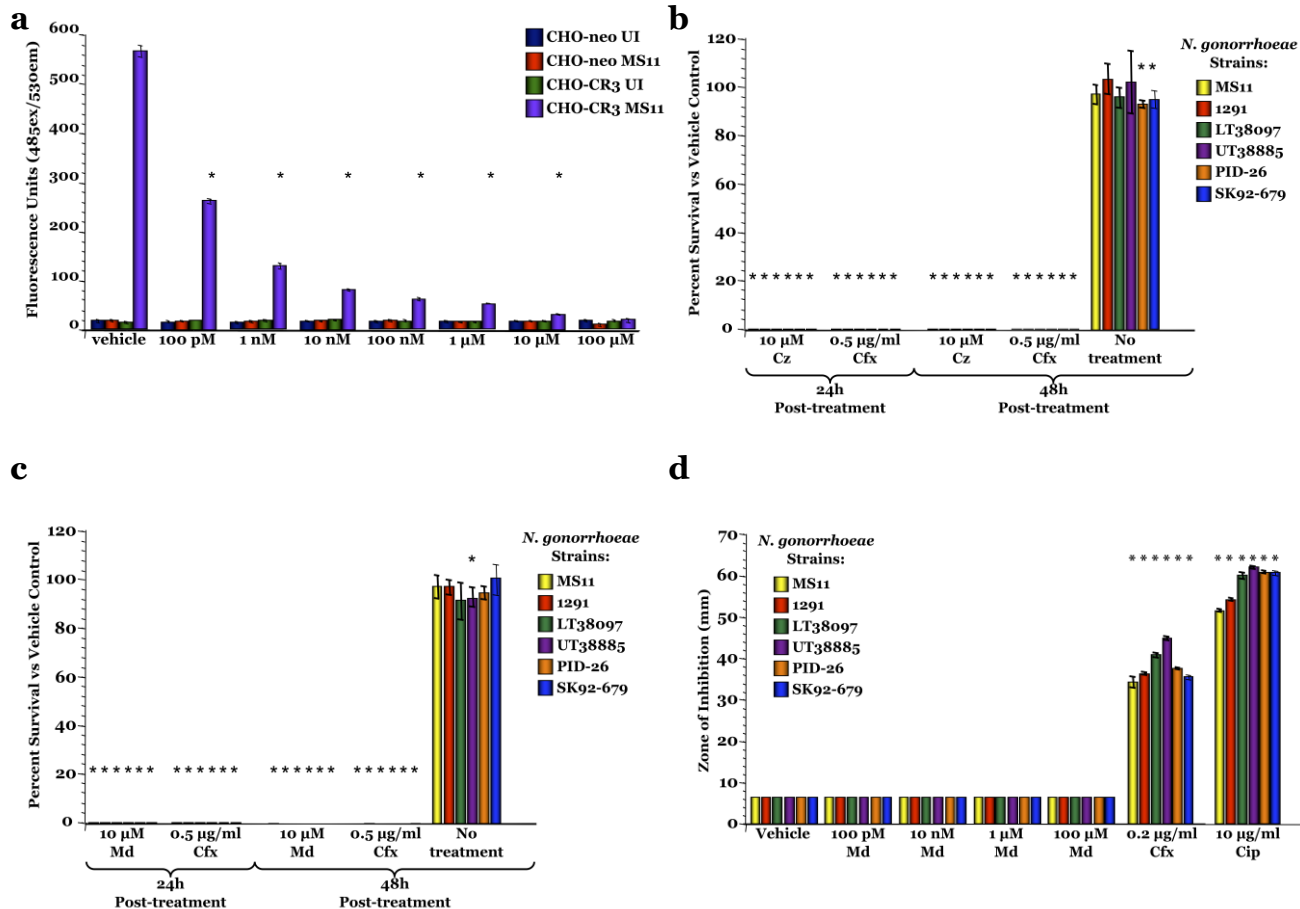

Supplement: FIG S6 [file mBio.03046-19-sf006.pdf]
