# Supplementary material for: Repurposed Drugs That Block the Gonococcus-Complement Receptor 3 Interaction Can Prevent and Cure Gonococcal Infection of Primary Human Cervical Epithelial Cells
Source: mBio. 2020 Mar 3;11(2):e03046-19. doi: 10.1128/mBio.03046-19 (PMC7064771; doi:10.1128/mBio.03046-19)
Supplement: TABLE S1 [file mBio.03046-19-st001.docx]

**Table S1. Peptide List.**

| **Peptide** | **Peptide Sequence** |
| --- | --- |
| **I-Domain Peptide Library** | |
| A1 | H-GSNLRQQPQKFPEAL-OH |
| B1 | H-QQPQKFPEALRGCPQ-OH |
| C1 | H-FPEALRGCPQEDSDI-OH |
| D1 | H-RGCPQEDSDIAFLID-OH |
| E1 | H-EDSDIAFLIDGSGSI-OH |
| F1 | H-AFLIDGSGSIIPHDF-OH |
| G1 | H-GSGSIIPHDFRRMKE-OH |
| H1 | H-IPHDFRRMKEFVSTV-OH |
| A2 | H-RRMKEFVSTVMEQLK-OH |
| B2 | H-FVSTVMEQLKKSKTL-OH |
| C2 | H-MEQLKKSKTLFSLMQ-OH |
| D2 | H-KSKTLFSLMQYSEEF-OH |
| E2 | H-FSLMQYSEEFRIHFT-OH |
| F2 | H-YSEEFRIHFTFKEFQ-OH |
| G2 | H-RIHFTFKEFQNNPNP-OH |
| H2 | H-FKEFQNNPNPRSLVK-OH |
| A3 | H-NNPNPRSLVKPITQL-OH |
| B3 | H-RSLVKPITQLLGRTH-OH |
| C3 | H-PITQLLGRTHTATGI-OH |
| D3 | H-LGRTHTATGIRKVVR-OH |
| E3 | H-TATGIRKVVRELFNI-OH |
| F3 | H-RKVVRELFNITNGAR-OH |
| G3 | H-ELFNITNGARKNAFK-OH |
| H3 | H-TNGARKNAFKILVVI-OH |
| A4 | H-KNAFKILVVITDGEK-OH |
| B4 | H-ILVVITDGEKFGDPL-OH |
| C4 | H-TDGEKFGDPLGYEDV-OH |
| D4 | H-FGDPLGYEDVIPEAD-OH |
| E4 | H-GYEDVIPEADREGVI-OH |
| F4 | H-IPEADREGVIRYVIG-OH |
| G4 | H-REGVIRYVIGVGDAF-OH |
| H4 | H-RYVIGVGDAFRSEKS-OH |
| A5 | H-VGDAFRSEKSRQELN-OH |
| B5 | H-RSEKSRQELNTIASK-OH |
| C5 | H-RQELNTIASKPPRDH-OH |
| D5 | H-TIASKPPRDHVFQVN-OH |
| E5 | H-PPRDHVFQVNNFEAL-OH |
| F5 | H-VFQVNNFEALKTIQN-OH |
| G5 | H-NFEALKTIQNQLREK-OH |
| H5 | H-ALKTIQNQLREKIFA-OH |
| **Control Peptides** | |
| P1 | H-CNNPSSTVPHNVDFH-OH |
| P2 | H-CATFTAPGRTSTFSF-OH |
| **Biotinylated Peptide** | |
| Biotin-AhxG2 | Biotin-Ahx-RIHFTFKEFQNNPNP-OH |
