# Supplementary material for: Repurposed Drugs That Block the Gonococcus-Complement Receptor 3 Interaction Can Prevent and Cure Gonococcal Infection of Primary Human Cervical Epithelial Cells
Source: mBio. 2020 Mar 3;11(2):e03046-19. doi: 10.1128/mBio.03046-19 (PMC7064771; doi:10.1128/mBio.03046-19)
Supplement: FIG S4 [file mBio.03046-19-sf004.pdf]

a)

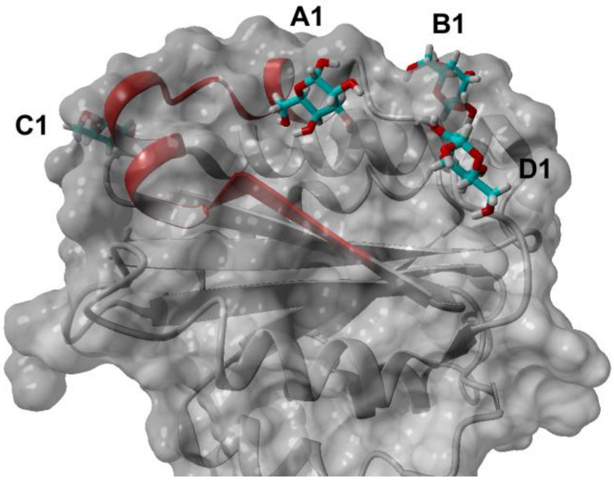

b)

Cluster	Bind. energy [kcal/mol]	Contacting receptor residues	Populated Conformers [%]
**A1**	3.955	ARG 181, HIS 183, PHE 184, LEU 198, PRO 201, ILE 202, THR 203	70.0
**B1**	3.5740	PRO 147, PHE 150, ARG 151, LYS 154, LYS 200, PRO 201, ILE 202, THR 203, GLN 204	10.0
**C1**	3.6320	LYS 165, LYS 166, SER 167, THR 169, LEU 170, PHE 189, GLN 190, PRO 193	15.0
**D1**	3.5210	GLU 179, ARG 181, THR 203, GLN 204, LEU 205, LEU A 206	5.0
